# Interim results from an ongoing, open-label, single-arm trial of odevixibat in progressive familial intrahepatic cholestasis

**DOI:** 10.1016/j.jhepr.2023.100782

**Published:** 2023-04-29

**Authors:** Richard J. Thompson, Reha Artan, Ulrich Baumann, Pier Luigi Calvo, Piotr Czubkowski, Buket Dalgic, Lorenzo D’Antiga, Angelo Di Giorgio, Özlem Durmaz, Emmanuel Gonzalès, Tassos Grammatikopoulos, Girish Gupte, Winita Hardikar, Roderick H.J. Houwen, Binita M. Kamath, Saul J. Karpen, Florence Lacaille, Alain Lachaux, Elke Lainka, Kathleen M. Loomes, Cara L. Mack, Jan P. Mattsson, Patrick McKiernan, Quanhong Ni, Hasan Özen, Sanjay R. Rajwal, Bertrand Roquelaure, Eyal Shteyer, Etienne Sokal, Ronald J. Sokol, Nisreen Soufi, Ekkehard Sturm, Mary Elizabeth Tessier, Wendy L. van der Woerd, Henkjan J. Verkade, Jennifer M. Vittorio, Terese Wallefors, Natalie Warholic, Qifeng Yu, Patrick Horn, Lise Kjems

**Affiliations:** 1 Institute of Liver Studies, King’s College London, London, UK; 2 Department of Pediatric Gastroenterology, Akdeniz University, Antalya, Turkey; 3 Pediatric Gastroenterology and Hepatology, Hannover Medical School, Hannover, Germany; 4 Pediatric Gastroenterology Unit, Regina Margherita Children’s Hospital, Azienda Ospedaliera-Città della Salute e della Scienza di Torino, Turin, Italy; 5 Department of Gastroenterology, Hepatology, Nutritional Disorders, and Pediatrics, The Children’s Memorial Health Institute, Warsaw, Poland; 6 Department of Pediatric Gastroenterology, Gazi University Faculty of Medicine, Ankara, Turkey; 7 Pediatric Hepatology, Gastroenterology, and Transplantation, Azienda Ospedaliera Papa Giovanni XXIII, Bergamo, Italy; 8 Istanbul University, Istanbul Faculty of Medicine, Istanbul, Turkey; 9 Hépatologie et Transplantation Hépatique Pédiatriques, Centre de Référence de l’Atrésie des Voies Biliaires et des Cholestases Génétiques, FSMR FILFOIE, ERN RARE LIVER, Hôpital Bicêtre, AP-HP, Université Paris-Saclay, Hépatinov, Inserm U 1193, Paris, France; 10 Pediatric Liver, GI, and Nutrition Center and MowatLabs, King’s College Hospital NHS Trust, London, UK; 11 Liver Unit and Small Bowel Transplantation, Birmingham Women’s and Children’s NHS Foundation Trust, Birmingham, UK; 12 Department of Gastroenterology, Royal Children's Hospital, Melbourne, Australia; 13 Department of Pediatric Gastroenterology at the Wilhelmina Children’s Hospital and University Medical Center, Utrecht, The Netherlands; 14 Division of Gastroenterology, Hepatology, and Nutrition, Hospital for Sick Children and the University of Toronto, Toronto, ON, Canada; 15 Pediatrics Department, Emory University School of Medicine, Children’s Healthcare of Atlanta, Atlanta, GA, USA; 16 Pediatric Gastroenterology-Hepatology-Nutrition Unit, Hôpital Universitaire Necker-Enfants Malades, Paris, France; 17 Hospices Civils de Lyon, Hôpital Femme-Mère-Enfant, Service D’hépatogastoentérologie et Nutrition Pédiatrique, Lyon, France; 18 Department of Pediatric Gastroenterology, Hepatology, and Liver Transplantation, University Children’s Hospital, Essen, Germany; 19 Department of Pediatrics, Division of Gastroenterology, Hepatology and Nutrition, The Children’s Hospital of Philadelphia, Philadelphia, PA, USA; 20 Pediatric Gastroenterology, Hepatology, & Nutrition, Children’s Hospital of Wisconsin, Medical College of Wisconsin, Milwaukee, WI, USA; 21 Albireo Pharma, Inc., Boston, MA, USA; 22 Division of Pediatric Gastroenterology, Hepatology, and Nutrition, Hacettepe University Faculty of Medicine, Ankara, Turkey; 23 Children’s Liver Unit, Leeds Teaching Hospitals NHS Trust, Leeds Children’s Hospital, Leeds, UK; 24 CHU, Hospital de la Timone, Marseille, France; 25 Faculty of Medicine, Hebrew University of Jerusalem, Juliet Keidan Department of Pediatric Gastroenterology, Shaare Zedek Medical Center, Jerusalem, Israel; 26 Université Catholique de Louvain, Cliniques St Luc, Brussels, Belgium; 27 University of Colorado School of Medicine, Children’s Hospital Colorado, Aurora, CO, USA; 28 Pediatrics Department, Children's Hospital Los Angeles, Los Angeles, CA, USA; 29 Pediatric Gastroenterology and Hepatology, University Children’s Hospital Tübingen, Tübingen, Germany; 30 Department of Pediatrics, Section of Pediatric Gastroenterology, Hepatology, and Nutrition, Baylor College of Medicine/Texas Children’s Hospital, Houston, TX, USA; 31 Department of Pediatrics, University of Groningen, Beatrix Children’s Hospital/University Medical Center Groningen, Groningen, The Netherlands; 32 Department of Surgery, Center for Liver Disease and Transplantation, Columbia University Medical Center, New York, NY, USA

**Keywords:** Liver diseases, Bile acids and salts, Clinical trial, Enterohepatic circulation

## Abstract

**Background & Aims:**

PEDFIC 2, an ongoing, open-label, 72-week study, evaluates odevixibat, an ileal bile acid transporter inhibitor, in patients with progressive familial intrahepatic cholestasis.

**Methods:**

PEDFIC 2 enrolled and dosed 69 patients across two cohorts; all received odevixibat 120 μg/kg per day. Cohort 1 comprised children from PEDFIC 1, and cohort 2 comprised new patients (any age). We report data through 15 July 2020, with Week 24 of PEDFIC 2 the main time point analysed. This represents up to 48 weeks of cumulative exposure for patients treated with odevixibat from the 24-week PEDFIC 1 study (cohort 1A) and up to 24 weeks of treatment for those who initiated odevixibat in PEDFIC 2 (patients who received placebo in PEDFIC 1 [cohort 1B] or cohort 2 patients). Primary endpoints for this prespecified interim analysis were change from baseline to Weeks 22–24 in serum bile acids (sBAs) and proportion of positive pruritus assessments (≥1-point drop from PEDFIC 2 baseline in pruritus on a 0–4 scale or score ≤1) over the 24-week period. Safety monitoring included evaluating treatment-emergent adverse events (TEAEs).

**Results:**

In cohort 1A, mean change from PEDFIC 1 baseline to Weeks 22–24 of PEDFIC 2 in sBAs was -201 μmol/L (*p* <0.0001). For cohort 1B and cohort 2, mean changes from odevixibat initiation to weeks 22–24 in sBAs were -144 and -104 μmol/L, respectively. The proportion of positive pruritus assessments in the first 24-week period of PEDFIC 2 was 33%, 56%, and 62% in cohorts 1A, 1B, and 2, respectively. Most TEAEs were mild or moderate. No drug-related serious TEAEs occurred.

**Conclusions:**

Odevixibat in patients with progressive familial intrahepatic cholestasis was generally well tolerated and associated with sustained reductions in sBAs and pruritus.

**Clinical Trials Registration:**

This study is registered at ClinicalTrials.gov (NCT03659916).

**Impact and Implications:**

Disrupted bile flow is a hallmark feature of patients with progressive familial intrahepatic cholestasis and can result in build-up of bile constituents in the liver with spill over into the bloodstream; other effects that patients can experience include extremely itchy skin, and because not enough bile reaches the gut, patients can have problems digesting food, which may lead to poor growth. Odevixibat is an orally administered medication that shunts bile acids away from the liver. The current study, called PEDFIC 2, suggested that odevixibat can improve the problematic signs and symptoms of progressive familial intrahepatic cholestasis and was generally safe for patients.

## Highlights

• PEDFIC 2 enrolled patients from PEDFIC 1 or new patients with PFIC; all received odevixibat 120 μg/kg per day.
• Odevixibat was associated with mean improvements in serum bile acids, pruritus, growth, hepatic parameters, and sleep.
• Treatment effects were durable up to Week 24 of PEDFIC 2.
• Odevixibat was generally well tolerated.

## Graphical abstract

**Figure:**
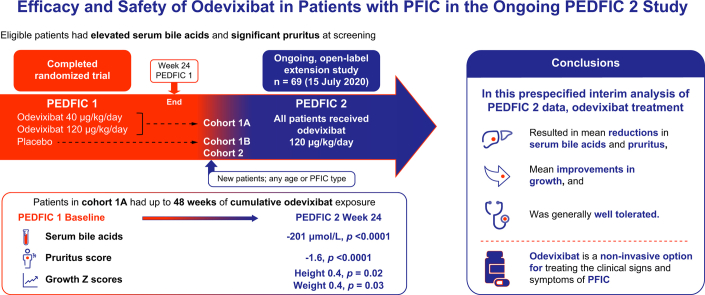

## Introduction

Progressive familial intrahepatic cholestasis (PFIC) is a group of liver diseases resulting from mutations in genes encoding proteins with diverse functions, including familial intrahepatic cholestasis protein 1 (FIC1), bile salt export pump (BSEP), and multidrug resistance protein 3 (MDR3) or PFIC1, PFIC2, and PFIC3, respectively.^1^ Children with PFIC may experience high serum bile acid concentrations, intractable pruritus, impaired growth, and progressive liver disease.^2^ Historically, long-term, effective treatment options for PFIC were limited to surgical interruption of the enterohepatic circulation (*i.e.* biliary diversion surgery) and liver transplantation.^3^

The ileal bile acid transporter (IBAT) resorbs intestinal bile acids for recirculation to the liver and is a therapeutic target for pharmacologic interruption of the enterohepatic circulation.^4^^,^^5^ Odevixibat, an orally administered IBAT inhibitor, markedly decreases ileal bile acid reuptake with minimal systemic exposure^6^ and is indicated for the treatment of pruritus in patients 3 months of age and older with PFIC in the USA and for the treatment of PFIC in patients age 6 months and older in the European Union and the UK.^7^^,^^8^

In the phase III PEDFIC 1 study, odevixibat produced statistically significant reductions in serum bile acids and pruritus relative to placebo in children with PFIC1 and PFIC2 and was generally well tolerated over 24 weeks.^9^ The current study, PEDFIC 2, is an ongoing, open-label extension study evaluating long-term efficacy and safety of odevixibat in patients with PFIC.

## Participants and methods

PEDFIC 2 (NCT03659916) was conducted in accordance with the Declaration of Helsinki and the International Conference on Harmonization guidelines for Good Clinical Practice. Research protocols and amendments were approved by relevant institutional review boards and ethics committees at each site (Table S1), and patients (or their caregivers) provided written informed consent.

### Study design and treatments

**Fig. 1:**
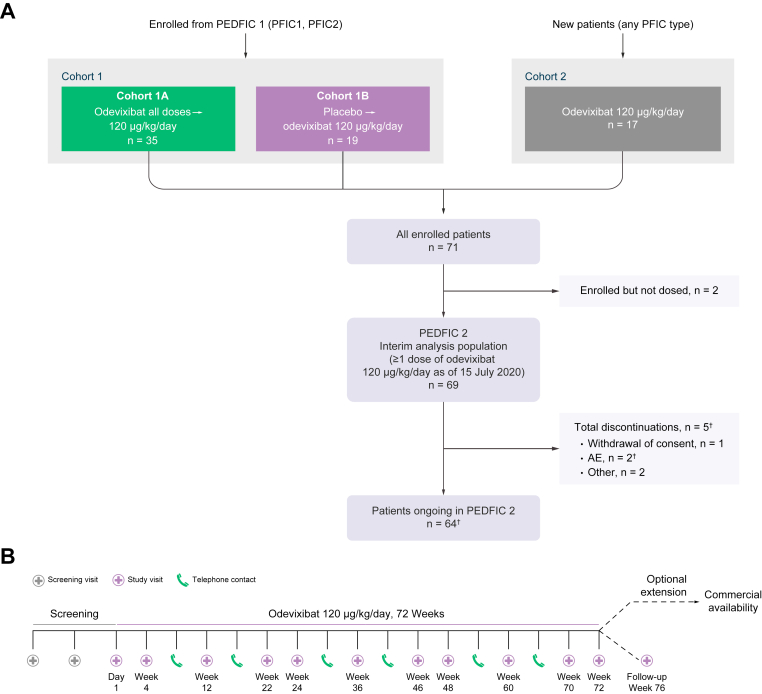
Patient disposition (A) and study design (B). (A) New patients in cohort 2 either did not meet PEDFIC 1 eligibility criteria or presented after closure of that study’s recruitment. ^†^One patient withdrew from treatment owing to AEs, but this was not reflected in the termination record at the time of data cut-off. (B) Cohort 2 screening visits occurred during Days 56−35 and 28−7 before the first odevixibat dose. For patients in cohort 1, the first visit of the PEDFIC 2 treatment period coincided with the PEDFIC 1 end-of-treatment visit (there was no interruption of treatment). AE, adverse event.

Eligible patients were enrolled into one of two cohorts (Fig. 1A). Cohort 1 comprised patients with PFIC1 or PFIC2 (aged 0.5–18 years) who completed the full 24-week treatment period in PEDFIC 1 or early rollovers (see below). These patients had received either odevixibat (40 or 120 μg/kg per day) or placebo in PEDFIC 1 (*i.e.* cohort 1A and cohort 1B, respectively). Initially, patients who withdrew from PEDFIC 1 because of perceived intolerable symptoms after ≥12 weeks could rollover early into PEDFIC 2; however, this option was later eliminated by a PEDFIC 1 protocol amendment.

Cohort 2 consisted of newly enrolled patients of any age with any PFIC type. One patient in cohort 2 had been previously treated with odevixibat in a phase II study approximately 3 years prior; all other patients in cohort 1B and cohort 2 were naive to odevixibat at the start of the study.

The PEDFIC 2 study included a screening period (for cohort 2 only) and a 72-week treatment period (Fig. 1B). During the treatment period, all patients received once-daily odevixibat 120 μg/kg. The odevixibat dose could have been down-titrated to 40 μg/kg per day after ≥1 week of treatment for tolerability concerns, with a return to the higher dose as soon as was considered clinically appropriate. Following the 72-week treatment period, patients could either enrol in an optional extension for continued treatment or return for a follow-up visit 4 weeks after ending study drug. A prespecified interim analysis of PEDFIC 2 data was conducted to fulfil regulatory requirements and supplement the findings of the PEDFIC 1 study; this interim analysis included data collected in PEDFIC 2 through a cut-off date of 15 July 2020.

### Eligibility criteria

Eligible patients were those with genetically confirmed PFIC, elevated serum bile acids (≥100 μmol/L), and significant pruritus (*i.e.* scratching or itching score ≥2 per caregiver/patient report [*i.e.* observer-reported outcome [ObsRO]/patient-reported outcome [PRO] based on PRUCISION scores; see Supplementary Materials & methods for details). Those with known pathologic variations of the *ABCB11* gene that resulted in complete absence of the BSEP protein were excluded.

### Assessments, endpoints, and analysis

#### Assessments and endpoints

The primary endpoints of the overall PEDFIC 2 study are change from baseline to the average of values at weeks 70 and 72 in serum bile acids and proportion of positive pruritus assessments at the patient level (*i.e.* scratching score ≤1 or a ≥1-point drop from PEDFIC 2 baseline) over the 72-week period. ‘Positive pruritus assessment’ was the terminology agreed upon with the United States Food and Drug Administration to describe this pruritus endpoint. For this prespecified interim analysis, the same outcomes were evaluated at Weeks 22–24 or over the first 24-week period of PEDFIC 2, respectively.

Blood samples for serum bile acid assessments were drawn at all study visits from Day 1 for those in cohort 1, or during screening for those in cohort 2, through Week 76. Caregivers/patients used the validated PRUCISION instrument twice daily in an eDiary format to record pruritus symptoms (and sleep characteristics or tiredness).^10^ Scores range from 0 to 4, with higher scores indicating worse symptoms (see Supplementary Materials & methods for more details).

Secondary and exploratory outcomes assessed included mean changes in serum bile acids at time points in addition to PEDFIC 2 Weeks 22–24; mean changes in pruritus scores over time; the proportion of patients meeting criteria for treatment response; and the effects of odevixibat on growth, sleep, biliary diversion and/or liver transplantation, and markers of cholestasis and liver disease. Native liver survival was assessed as an *ad hoc* supplementary analysis in addition to pre-planned analyses. Thresholds for serum bile acid response were based on levels from the NAtural course and Prognosis of PFIC and Effect of biliary Diversion (NAPPED) consortium that predict better long-term outcomes (serum bile acid levels <65 or <102 μmol/L for patients with PFIC1^11^ and PFIC2,^12^ respectively) or were prespecified in the study protocol (serum bile acid levels ≤70 μmol/L or reduced ≥70% from baseline). Pruritus response was defined as ≥1-point reduction in pruritus score from baseline (*i.e.* a clinically meaningful change).

Safety assessments included adverse event (AE) monitoring, physical examination, measurement of clinical laboratory parameters, and results of abdominal ultrasounds. In addition, because patients with PFIC may experience multiple comorbidities (*i.e.* fat-soluble vitamin deficiencies, diarrhoea, altered hepatic parameters), AEs of interest related to these comorbidities were also summarised (see Supplementary Materials & methods).

#### Data analysis

This interim data cut focused on efficacy data at Week 24 in PEDFIC 2. This represents up to 48 weeks of cumulative odevixibat exposure for those treated with odevixibat in PEDFIC 1 (cohort 1A) and up to 24 weeks of treatment for those who initiated odevixibat in PEDFIC 2 (*i.e.* cohort 1B and cohort 2). Future PEDFIC 2 data cuts will include data for up to 96 weeks of odevixibat treatment for patients who initiated treatment in PEDFIC 1, and up to 72 weeks of odevixibat treatment for patients who initiated treatment in PEDFIC 2. All analyses were conducted in patients who received ≥1 dose of odevixibat. AEs were classified by preferred term according to the Medical Dictionary for Regulatory Activities (MedDRA) version 23.0. Additional details, including estimates of sample size, are included in the Supplementary Materials & methods.

#### Statistical methods

Descriptive statistics were mainly used for this open-label extension study. However, statistical tests were prespecified for some outcomes in cohort 1A, as follows: one-sample *t* tests were used to evaluate statistical significance in change from PEDFIC 1 baseline to the average of values at Weeks 22 and 24 of PEDFIC 2 in serum bile acids, change from PEDFIC 1 baseline to Weeks 21–24 of PEDFIC 2 in pruritus scores, and change from PEDFIC 1 baseline to Week 24 in height and weight Z scores.

## Results

### Study population

As of 15 July 2020, 69 patients have received treatment in PEDFIC 2 (Fig. 1A), including 53 patients who rolled over from PEDFIC 1 (*i.e.* cohort 1; see Supplementary Materials & methods for more details) and 16 newly enrolled patients (*i.e.* cohort 2). Although part of this study occurred during the COVID-19 pandemic, no patients discontinued as a result. Most treated patients are ongoing on therapy (64/69, 93%). Overall, four patients discontinued as of the data cut-off date: three in cohort 1 (one because of withdrawal of consent, one because of an AE of cholestasis and subsequent surgical biliary diversion, and one who underwent liver transplantation) and one in cohort 2 (because of withdrawal of consent and acute pancreatitis). One additional patient was off treatment, but the termination record was not complete at the time of data cut-off.

Table 1 displays patient demographics and characteristics at baseline of PEDFIC 1 and PEDFIC 2. PEDFIC 2 included a mostly young patient population: at baseline, the median patient age was 4.1 years. More than half of patients had PFIC2 (n = 45), 18 patients had PFIC1, five patients had PFIC3, and one patient had myosin 5B deficiency. The median time since PFIC diagnosis was 2.6 years.

**Table 1:** **Patient demographics and baseline characteristics**.

	PEDFIC 1 baseline†		PEDFIC 2 baseline		
	Received odevixibat in PEDFIC 1 n = 42	Received placebo in PEDFIC 1 n = 20	Cohort 1A‡ n = 34	Cohort 1B§ n = 19	Cohort 2 n=16
Age, mean (SD), years¶	4.5 (3.9)	3.8 (3.9)	4.6 (3.6)	4.3 (4.0)	7.9 (4.9)
Female, n (%)	23 (55)	8 (40)	18 (53)	7 (37)	9 (56)
Race, n (%)					
White	35 (83)	17 (85)	29 (85)	16 (84)	15 (94)
Black	2 (5)	0	1 (3)	0	0
Asian	1 (2)	1 (5)	1 (3)	1 (5)	0
Other	4 (10)	2 (10)	3 (9)	2 (11)	1 (6)
Height, mean (SD), cm	95 (21)	89 (24)	97 (19)	93 (24)	115 (25)
Weight, mean (SD), kg	16 (9.6)	15 (9.8)	17 (8.3)	16 (11)	25 (16)
PFIC type, n (%)					
PFIC1	12 (29)	5 (25)	10 (29)	5 (26)	3 (19)
PFIC2	30 (71)	15 (75)	24 (71)	14 (74)	7 (44)
PFIC3	NA	NA	NA	NA	5 (31)
MYO5B deficiency	NA	NA	NA	NA	1 (6)
Use of UDCA at baseline, n (%)	32 (76)	18 (90)	23 (68)	17 (90)	13 (81)
Use of rifampicin at baseline, n (%)	24 (57)	17 (85)	15 (44)	17 (90)	7 (44)
Serum bile acids, mean (range), μmol/L	252 (36−605)	248 (57−435)	127 (1–439)	271 (11–528)	222 (11–465)
Pruritus score††, mean (range)	2.9 (1.6−4)	3.0 (1.9−4)	2.0 (0–4)	2.7 (1.3–4)	2.9 (2–4)
ALT, mean (range), U/L	110 (16−798)	77 (19−236)	74 (9–352)	71 (14–193)	70 (14–231)
AST, mean (range), U/L	106 (37−405)	90 (32−219)	71 (15−211)	82 (17–210)	97 (31–251)
Total bilirubin, mean (range), mg/dl	3.2 (0.2−18.6)	3.1 (0.3−11.4)	1.7 (0.1–12.3)	3.1 (0.2–19.8)	2.4 (0.7–7.0)

ALT, alanine aminotransferase; AST, aspartate aminotransferase; NA, not applicable; MYO5B, myosin 5B; PFIC, progressive familial intrahepatic cholestasis; UDCA, ursodeoxycholic acid.

† Data at PEDFIC 1 baseline are for all patients in PEDFIC 1.

‡ Patients in cohort 1A are a subset of patients who received odevixibat in PEDFIC 1.

§ Patients in cohort 1B are a subset of patients who received placebo in PEDFIC 1.

¶ For patients from France and Germany, only birth year is collected on the case report form and age is calculated based on collected age months and years from the external file.

†† AM and PM scores.

The median duration of odevixibat exposure was 43.1 weeks in cohort 1A and 36.1 weeks in cohort 1B; in cohort 2, which started enrolment approximately 1 year after the first patient in cohort 1 rolled over to PEDFIC 2, the median odevixibat exposure was 19.4 weeks. There were 25 patients in cohort 1A and 11 patients in cohort 1B with ≥24 weeks of odevixibat exposure at the PEDFIC 2 interim data cut; in cohort 2, five patients had ≥24 weeks of odevixibat exposure.

### Primary endpoints

#### Serum bile acids

**Fig. 2:**
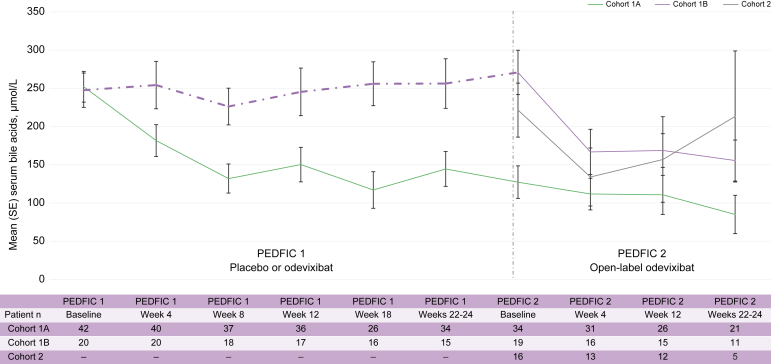
Change in serum bile acids during PEDFIC 1 and through PEDFIC 2 Week 24. PEDFIC 1 time points represent all PEDFIC 1 patients (odevixibat group, n = 42; placebo group, n = 20); values shown for PEDFIC 2 time points represent only the patients in PEDFIC 2 (cohort 1A, n = 34; cohort 1B, n = 19; cohort 2, n = 16). Dashed purple line indicates period of placebo administration. In cohort 1A, a significant change was observed in serum bile acids from PEDFIC 1 baseline to PEDFIC 2 weeks 22–24 (mean change: -201 μmol/L; 95% CI: -281 μmol/L, -121 μmol/L; *p* <0.0001 [one-sample *t* test]).

For patients in cohort 1A, the mean (SE) change in serum bile acids from PEDFIC 1 baseline to Weeks 22–24 in PEDFIC 2 (n = 21) was -201 μmol/L (38 μmol/L), *p* <0.0001; 95% CI: -281 μmol/L, -121 μmol/L. Data for these patients by prior odevixibat dose in PEDFIC 1 are presented in Table S2. Mean serum bile acid levels over time for all cohorts are shown in Fig. 2; individual changes in serum bile acids by PFIC type are presented in Fig. S1. For patients in cohort 1B and cohort 2 with available data at Weeks 22–24 (n = 11 and n = 5, respectively), mean (SE) changes from baseline in these patients were -144 μmol/L (49 μmol/L) and -104 μmol/L (39 μmol/L), respectively.

#### Pruritus

**Fig. 3:**
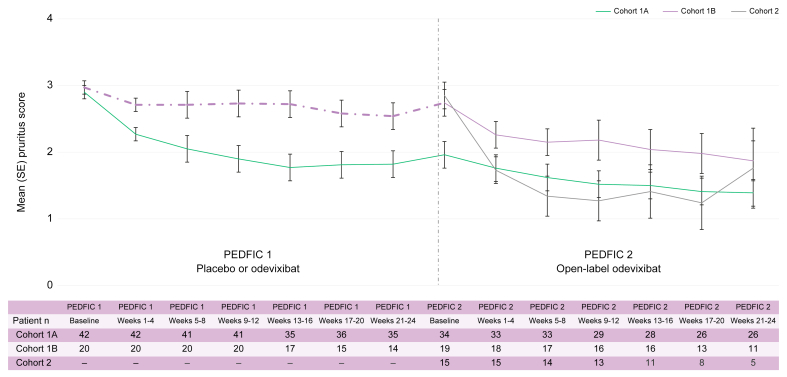
Change in pruritus scores during PEDFIC 1 and through PEDFIC 2 Week 24. PEDFIC 1 time points represent all PEDFIC 1 patients (odevixibat group, n = 42; placebo group, n = 20); values shown for PEDFIC 2 time points represent only the patients in PEDFIC 2 (cohort 1A, n = 34; cohort 1B, n = 19; cohort 2, n = 16). Dashed purple line indicates period of placebo administration. In cohort 1A, a significant change was observed in pruritus score from PEDFIC 1 baseline to PEDFIC 2 weeks 21–24 (mean change: -1.6; 95% CI: -2.0, -1.1; *p* <0.0001 [one-sample *t* test]).

The mean (SE) proportion of positive (*i.e.* improved) pruritus assessments at the patient level during 24 weeks of treatment in PEDFIC 2 was 33% (7%) in cohort 1A (n = 26), 56% (11%) in cohort 1B (n = 11), and 62% (20%) in cohort 2 (n = 5). For patients in cohort 1A, Table S2 presents pruritus data by prior dose of odevixibat in PEDFIC 1. Additionally, mean (SE) change in monthly pruritus score (based on ObsRO assessments) from PEDFIC 1 baseline to Weeks 21–24 in PEDFIC 2 in cohort 1A patients (n = 26) was -1.6 (0.2); *p* <0.0001; 95% CI: -2.0, -1.1. Fig. 3 shows pruritus scores over time for all cohorts.

### Additional efficacy endpoints

#### Other analyses related to serum bile acids and pruritus

From PEDFIC 1 baseline through Week 24 in PEDFIC 2, 18 of 33 patients (55%) with data in the interval had a serum bile acid response based on published criteria^11^^,^^12^ and 18 of 34 patients (53%) had a serum bile acid response based on criteria defined in PEDFIC 1 (Table S3).^9^ For those who initiated odevixibat in PEDFIC 2 (*i.e.* cohort 1B and cohort 2), six of 22 (27%) and 11 of 31 (36%), respectively, met published or PEDFIC 1 serum bile acid response criteria through PEDFIC 2 Week 24 (Table S3). Additional responder analyses, such as those based on pruritus, are also depicted in Table S3.

For patients in cohort 1A who received odevixibat 40 μg/kg per day in PEDFIC 1 and transitioned to odevixibat 120 μg/kg per day in PEDFIC 2, some patients had enhanced pruritus responses after this dose escalation; outcomes for these patients related to serum bile acid response were somewhat variable (Table S4).

Odevixibat produced generally consistent treatment effects on serum bile acids and proportion of positive pruritus assessments across all subgroups examined (*i.e.* based on demographic characteristics, baseline serum bile acid levels, degree of hepatic impairment, baseline use of ursodeoxycholic acid [UDCA] and rifampicin [data not shown]). Additionally, effects with odevixibat were observed across PFIC types (Table 2). For example, among odevixibat-naive patients (*i.e.* cohort 1B and cohort 2), mean reductions in serum bile acids were observed through PEDFIC 2 week 12 regardless of PFIC diagnosis (mean [n; SE] change from baseline: -32 μmol/L [n = 5; 42 μmol/L], -121 μmol/L [n = 17; 43 μmol/L], and -127 μmol/L [n = 4; 20 μmol/L] for those with PFIC1, 2, and 3, respectively).

**Table 2:** **Changes in serum bile acids and percentage of positive pruritus assessments by PFIC type from PEDFIC 2 baseline through Week 24 of PEDFIC 2**.

	Serum bile acids, μmol/L						Proportion of positive pruritus assessments, %			
	Baseline		Change from baseline to Week 12		Change from baseline to Weeks 22–24		Initial interval†		Weeks 0–24	
	n	Mean (SE)	n	Mean (SE)	n	Mean (SE)	n	Mean (SE)	n	Mean (SE)
PFIC1										
Cohort 1A	10	154 (35)	7	-14 (10)	5	-27 (14)	10	27 (10)	7	24 (10)
Cohort 1B	5	206 (28)	4	-40 (53)	3	-82 (31)	5	33 (17)	3	15 (7)
Cohort 2	3	121 (59)	1	-1.5	NA	NA	3	62 (19)	NA	NA
PFIC2										
Cohort 1A	24	116 (27)	19	4 (24)	16	-15 (15)	23	24 (6)	19	36 (8)
Cohort 1B	14	294 (37)	11	-157 (48)	8	-167 (65)	13	53 (9)	8	72 (10)
Cohort 2	7	279 (64)	6	-54 (85)	4	-96 (49)	6	62 (14)	3	41 (28)
PFIC3‡										
Cohort 2	5	212 (48)	4	-127 (20)	1	-136	5	95 (2)	1	94
MYO5B deficiency‡										
Cohort 2	1	169	1	-45	NA	NA	1	87	1	91

All patients in PEDFIC 2 receive odevixibat. For those who received odevixibat in PEDFIC 1, mean serum bile acid levels at PEDFIC 1 baseline were 226 μmol/L in patients with PFIC1 and 263 μmol/L in patients with PFIC2; for those who received placebo in PEDFIC 1, patients with PFIC1 and PFIC2 had mean baseline serum bile acid levels of 200 μmol/L and 263 μmol/L, respectively. The proportion of positive pruritus assessments from Weeks 0–24 during PEDFIC 1 was 61% in patients with PFIC1 and 51% in patients with PFIC2 among those who received odevixibat and 22% and 31%, respectively, among those who received placebo.

MYO5B, myosin 5B; NA, not applicable; PFIC, progressive familial intrahepatic cholestasis.

† Represents Weeks 0–4 for patients with PFIC1 and PFIC2 and 0–12 for patients with PFIC3 and MYO5B deficiency.

‡ Data are only available for these patient types in cohort 2, per study eligibility criteria for PEDFIC 1 and PEDFIC 2.

#### Growth

**Fig. 4:**
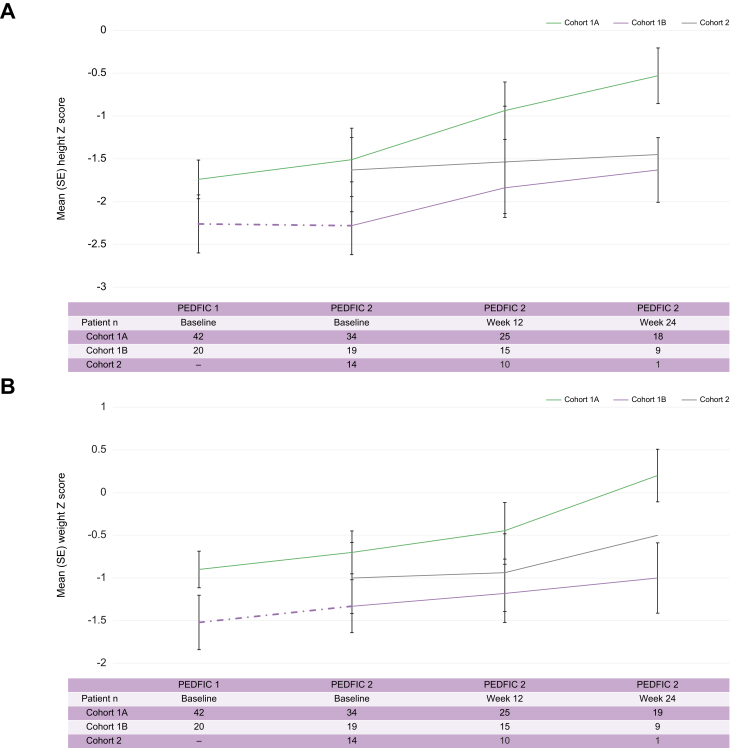
Effects of odevixibat on height (A) and weight (B) from PEDFIC 1 baseline through PEDFIC 2 Week 24. PEDFIC 1 values represent all patients in PEDFIC 1 (odevixibat group, n = 42; placebo group, n = 20); PEDFIC 2 time points represent only patients in PEDFIC 2 (cohort 1A, n = 34; cohort 1B, n = 19; cohort 2, n = 16). Dashed purple lines indicate placebo period. In cohort 1A, significant changes were observed in height and weight Z scores from PEDFIC 1 baseline to PEDFIC 2 Week 24 (mean changes: 0.4 [95% CI: 0.1, 0.7; *p* = 0.02] and 0.4 [95% CI: 0.0, 0.7; *p* = 0.03], respectively [one-sample *t* tests]).

Mean (SE) changes in height and weight Z scores in cohort 1A from PEDFIC 1 baseline through Week 24 of PEDFIC 2 were 0.4 (0.1; n = 18; *p* = 0.02; 95% CI: 0.1, 0.7) and 0.4 (0.2; n = 19; *p* = 0.03; 95% CI: 0.0, 0.7), respectively. Changes in growth over time for all cohorts are shown in Figs 4A and B; those in cohort 1B and cohort 2 also had mean improvements in height and weight Z scores following odevixibat initiation in PEDFIC 2.

#### Sleep

Patients treated with odevixibat in PEDFIC 1 had reductions in the percentage of days they needed help falling asleep, needed soothing, or slept with their caregiver.^9^ These improvements continued for cohort 1A patients in PEDFIC 2 (mean [SE] reductions from PEDFIC 2 baseline through the Week 24 of PEDFIC 2 were -12% [5%], -10% [5%], and -5% [5%], respectively; n = 26 for all). Sleep parameters also improved with 24 weeks of odevixibat treatment in PEDFIC 2 for those in cohorts 1B and 2 (mean [SE] values were -25% [12%], -18% [14%], and -10% [10%], respectively, for cohort 1B [n = 11 for all] and -53% [18%], -21% [23%], and -23% [19%], respectively, for cohort 2 [n = 5 for all]).

#### Surgical procedures

Surgical outcomes assessed using PEDFIC 2 data included the number of patients with surgical biliary diversion or liver transplantation; an *ad hoc* supplementary analysis also characterised native liver survival in patients with serum bile acid reductions or pruritus response with odevixibat. Of 69 patients who received odevixibat in PEDFIC 2, two (3%), both in cohort 1B, underwent surgical biliary diversion or had a liver transplantation during PEDFIC 2 owing to lack of improvement in pruritus. Both patients had PFIC2 (BSEP subtype 1)^12^ and stable hepatic status. One patient had biliary diversion surgery at Week 37 of PEDFIC 2; this patient did not experience a reduction in serum bile acids before surgery. The second patient had an elective liver transplantation at Week 19 of PEDFIC 2; the patient’s serum bile acids were reduced 25% at the last available assessment (Week 12) *vs.* baseline. In an *ad hoc* supplementary analysis of pooled data from patients treated with odevixibat from PEDFIC 1 and PEDFIC 2 to a data cut-off date of 31 January 2022, reductions in serum bile acid levels with odevixibat at 6 months of treatment were strongly associated with native liver survival for up to 3 years, with no patients who met serum bile acid response criteria undergoing liver transplant (Fig. S2). In addition, all patients with ≥1-point reduction in pruritus score at Month 6 remained liver transplant free during the same period (Fig. S2).

#### Changes in key liver function tests over time

Before initiation of odevixibat, most patients had elevated hepatic biochemical parameters (Table 1), per their underlying disease. Patients generally had improvements in these hepatic laboratory values with odevixibat, although some mean values remained elevated; in some cases, normalisation or levels near normalisation were reached (Table 3). Changes in other markers of liver disease are presented in Table S5.

**Table 3:** **Effects of odevixibat on markers of cholestasis through Week 24 of PEDFIC 2**.

	Week 12			Week 24		
	n	Mean (SE)	Mean (SE) change from baseline to Week 12	n	Mean (SE)	Mean (SE) change from baseline to Week 24
Serum ALT, U/L†						
Cohort 1A	26	54 (8)	-22 (13)	21	54 (16)	-27 (23)
Cohort 1B	16	83 (23)	12 (18)	9	99 (33)	16 (21)
Cohort 2	12	116 (33)	37 (27)	4	44 (28)	-10 (31)
Serum AST, U/L‡						
Cohort 1A	27	57 (6)	-13 (7)	20	54 (8)	-15 (10)
Cohort 1B	16	92 (18)	6 (11)	9	86 (15)	-1.7 (6)
Cohort 2	11	120 (34)	30 (23)	4	54 (21)	-7.8 (16)
Total bilirubin, mg/dl§						
Cohort 1A	26	1.3 (0.5)	0.0 (0.1)	21	0.9 (0.3)	-0.2 (0.1)
Cohort 1B	16	2.7 (0.7)	-0.6 (0.7)	9	2.3 (0.8)	-1.6 (1.3)
Cohort 2	12	2.9 (1.0)	0.8 (0.9)	4	1.3 (0.4)	-0.2 (0.5)

† Normal reference range varies by age and sex, but typical values are ≤55 U/L.

‡ Normal reference range varies by age and sex, but typical values are <79 U/L.

§ Normal reference range: ≤1.2 mg/dL. ALT, alanine aminotransferase; AST, aspartate aminotransferase.

### Safety

In this prespecified interim analysis of PEDFIC 2 data, 50 of 69 (72%) patients experienced at least one treatment-emergent AE (TEAE) (Table 4). Of these, 45 had mild or moderate TEAEs. The most commonly reported TEAEs in PEDFIC 2 at the data cut-off (occurring in ≥10% of patients overall) were upper respiratory tract infection (n = 14, 20%), pyrexia (n = 13, 19%), cough (n = 10, 15%), increased blood bilirubin (n = 9, 13%), and diarrhoea (n = 8, 12%). For patients with diarrhoea, all events were mild or moderate in severity. Six patients had diarrhoea events that were deemed unrelated or unlikely related to treatment, and two patients had events that were possibly or definitely related to treatment.

**Table 4:** **Summary of TEAEs during the PEDFIC 2 treatment period**.

	Cohort 1		Cohort 2 n = 16
Patients, n (%)	Cohort 1A n = 34	Cohort 1B n = 19	
Any TEAE	28 (82)	14 (74)	8 (50)
Mild	17 (50)	6 (32)	2 (13)
Moderate	10 (29)	7 (37)	3 (19)
Severe	1 (3)	1 (5)	3 (19)
TEAEs of diarrhoea	7 (21)	1 (5)	0
Drug-related TEAEs	10 (29)	5 (26)	5 (31)
Serious TEAEs	0	3 (16)	1 (6)
TEAEs leading to discontinuation	0	1 (5)	2 (13)
Drug-related TEAEs occurring in ≥5% of patients overall, by preferred term			
ALT increased	1 (3)	1 (5)	2 (13)
Blood bilirubin increased	3 (9)	2 (11)	2 (13)

All patients in PEDFIC 2 received odevixibat 120 μg/kg per day. Adverse events were untoward events that had worsened in a clinically significant manner from baseline, as assessed by investigators, and are presented by Standardized Medical Dictionary for Regulatory Activities preferred terms.

ALT, alanine aminotransferase; TEAE, treatment-emergent adverse event.

Three (4%) patients discontinued as a result of a TEAE (none of which were considered related to odevixibat); these included one patient in cohort 1B with a TEAE of cholestasis, one patient in cohort 2 with a medical history of chronic pancreatitis with a TEAE of acute pancreatitis, and one patient in cohort 2 with TEAEs of splenomegaly, jaundice, hypophagia, and decreased weight.

Drug-related TEAEs were reported in 20 of 69 (29%) patients. The most common drug-related TEAEs (occurring in ≥5% of patients overall) are shown in Table 4. Four of 69 (6%) patients experienced serious TEAEs, including three (16%) in cohort 1B and one (6%) in cohort 2. All serious AEs were assessed as unrelated to study treatment. No deaths occurred. Three patients underwent a dose reduction from 120 to 40 μg/kg per day (after elevated transaminases and/or bilirubin, n = 2; and after a TEAE of constipation, n = 1); two patients continued on this dose and 1 patient was off the study drug as of the data cut-off date.

With regard to AEs of interest, no patients experienced new or worsening fat-soluble vitamin deficiency refractory to clinically recommended vitamin supplementation based on mean changes in fat-soluble vitamin levels during the study (Table S6), and medical review of individual patient data uncovered none that met the criteria for clinically significant diarrhoea. Overall, 34 events in 26 patients underwent review and adjudication by the Data and Safety Monitoring Board for suspected drug-induced liver injury or liver-related events. All but one of these events were assessed as related to the patient’s underlying disease or other causes. The remaining event (increased alanine aminotransferase and total bilirubin) occurred in a patient in cohort 1B and was considered related to the study drug. No patients developed liver decompensation or had hepatic events reported in the Standardized MedDRA Query of *Drug Related Hepatic Disorders – Severe Events Only*. No clinically significant changes or safety signals were noted based on laboratory assessments or physical examinations.

## Discussion

Results from the ongoing open-label PEDFIC 2 study suggest that odevixibat 120 μg/kg per day can provide durable treatment effects in patients with PFIC. These interim data supplement the results from the preceding PEDFIC 1 study^9^ and extend to other PFIC types the observations that odevixibat is associated with reductions in serum bile acids and improvements in pruritus. Odevixibat also had a safety profile that was consistent with prior odevixibat studies in both healthy individuals and paediatric patients with cholestatic liver disease,^6^^,^^13^ with no unexpected AEs reported.

The characteristics of the patients included in this study were consistent with the known characteristics of patients with PFIC1, PFIC2, and PFIC3.^1^^,^^14^ For example, the majority of patients were receiving UDCA and/or rifampicin, conventional therapies for PFIC,^14^ at study baseline. Also, patients in cohort 2 were slightly older, which may be attributable to the expanded eligibility criteria in cohort 2; in addition, some patients such as those with PFIC3 may present later in life.^1^^,^^15^ These observations suggest that the results of this study can be generalised to the larger population of patients with PFIC.

Patients in cohort 1A (of whom 33 of 34 were ongoing on treatment as of the data cut-off date) who received odevixibat in PEDFIC 1 and entered PEDFIC 2 with improved serum bile acid levels generally had a durable effect with longer-term treatment. For patients in cohort 1B and cohort 2 who initiated odevixibat treatment in PEDFIC 2, reductions in serum bile acids were observed as early as Week 4 of treatment and generally continued through Week 24. Data from this interim analysis preliminarily suggest that response to odevixibat may improve over time, although longer-term studies are needed to confirm this finding.

The NAPPED consortium, which aims to detail the natural history of PFIC and uncover associations between treatments and long-term outcomes,^16^ analysed outcomes following surgical biliary diversion, a procedure which aims to divert bile acids out of the enterohepatic circulation and reduce the size of the bile acid pool, in patients with PFIC1 and found that lower serum bile acid levels (<65 μmol/L) post-surgery tended to be associated with prolonged native liver survival.^11^ Similarly, lower serum bile acid levels post diversion in patients with PFIC2 (<102 μmol/L or decreased ≥75%) reliably predicted native liver survival for ≥15 years.^12^ Here, we present *ad hoc* supplementary data on native liver survival in patients treated with odevixibat, a medical option to divert bile acids from the gut; this analysis indicates that all odevixibat-treated patients with a serum bile acid response to treatment retained their native livers, whereas those who did not meet serum bile acid response criteria had higher rates of liver transplantation (Fig. S2).

Significant pruritus can lead to severe cutaneous mutilation, loss of sleep, and multiple behavioural impairments.^2^^,^^17^ Thus, improvement in pruritus, and particularly night-time scratching, is a goal of therapy as it is of clinical benefit. Indeed, in the preceding PEDFIC 1 study, positive effects on patient and family quality of life were observed with odevixibat *vs.* placebo.^18^ Here, mean pruritus scores declined over the treatment period in all patients, with continued mean improvements in patients who received odevixibat in PEDFIC 1 and rolled over to PEDFIC 2 (*i.e.* cohort 1A) and overall mean improvements in patients who started odevixibat 120 μg/kg per day in PEDFIC 2 (*i.e.* cohorts 1B and 2). For patients naive to odevixibat, pruritus scores decreased within 4 weeks of initiating odevixibat in PEDFIC 2, with further decreases observed over time. Consistent with reductions in pruritus with odevixibat, numerical improvements in several measures of patient sleep were observed. Additionally, mean changes in height and weight through Week 24 of PEDFIC 2 suggested growth gains, observations that are consistent with growth changes previously reported following surgical biliary diversion in patients with PFIC.^19^

Mean improvements in serum bile acids and pruritus scores with odevixibat were generally accompanied by mean decreases in hepatic laboratory values that are commonly associated with paediatric cholestasis.^20^ End-stage liver disease resulting from prolonged cholestasis, or pruritus unresponsive to medical therapy or surgical diversion, are indications for liver transplantation in patients with PFIC,^4^^,^^21^^,^^22^ and preclinical evidence suggests IBAT inhibition may reduce hepatic inflammation or fibrosis.^19^^,^^23^^,^^24^

Some limitations warrant discussion. PEDFIC 2 was an open-label study, with no comparator arm. Although this design could make distinguishing treatment effects from regression to the mean and Hawthorne effects challenging, these study results are supported by data from the randomised, placebo-controlled PEDFIC 1 trial, where odevixibat treatment of patients with PFIC resulted in significant mean reductions in serum bile acids and pruritus *vs.* placebo.^9^ In addition, treatment effects in PEDFIC 2 were not determined by a single outcome, but rather were based on both biologic assessments (for serum bile acids) and report of observed symptoms (for pruritus). A final limitation is that some subgroups (*e.g.* those with PFIC3; patients in cohort 2 with available data at Week 24 of odevixibat treatment) were represented by small numbers of patients. Because cohort 2 was still enrolling at the time of the data cut-off, these subgroups may be larger in the final analysis.

Data from this ongoing, long-term study suggest that odevixibat has durable effects in PFIC, including improvements in serum bile acid levels, pruritus, growth, and hepatic measures, as well as survival with native liver in treatment responders. Odevixibat was generally well tolerated, reduced the systemic accumulation of bile acids that results from cholestasis, and improved pruritus. Together, these effects have the potential to improve hepatic health and delay or prevent liver transplantation in patients with PFIC. The totality of the evidence across multiple endpoints suggests the benefit of odevixibat for treating the clinical signs and symptoms associated with PFIC.

## Financial support

This study was sponsored by Albireo Pharma, Inc. who had input into the study design; in the collection, analysis, and interpretation of data; in the writing of the report; and in the decision to submit the paper for publication.

## Authors’ contributions

Coordinating investigator: RJT; Medical officer: PH; Design, conduct, and oversight of the clinical trial: LK, JPM, TW, NW, PH; Site investigators and participated in patient recruitment, treatment, data collection, and follow-up: RJT, RA, UB, PLC, PC, BD, LD, ADG, OD, EG, TG, GG, WH, RHJH, BMK, SJK, FL, AL, EL, KML, CLM, PM, HO, SRR, BR, EySh, EtSo, NS, EkSt, MET, WLV, HJV, JMV; Verification of the data: LK, PH; Critical review of the data: RJT, UB, LD, EG, BMK, SJK, KML, PM, EkSt, RJS, HJV; All authors contributed to the critical review, revision, and final approval of the manuscript.

## Data availability statement

Qualified academic investigators and researchers may request additional participant-level, de-identified clinical data, and supporting documents (statistical analysis plan and protocol) pertaining to this study. For details regarding data availability, instructions for requesting information, and our data disclosure policy please email us at medinfo@albireopharma.com.

## Conflicts of interest

RJT: Albireo and Mirum – Consultant; Generation Bio – Consultant and stock options; Rectify Therapeutics – Consultant and stockholder; LD: Albireo, Alexion, Mirum, Selecta, Vivet, Spark, Tome, and Genespire – Consultant; ADG: Albireo – Consultant; UB: Albireo, Mirum, Alnylam, Vivet, and Nestlé – Consultant; EG: Laboratoires C.T.R.S., Mirum, Vivet, and Albireo – Consultant; TG: Albireo – Consultant; RHJH: GMP-Orphan and Univar – Consultant; BMK: Albireo, Mirum, and Audentes – Consultant; Albireo and Mirum – Unrestricted educational grants; SJK: Albireo, HemoShear, Intercept, Mirum, and Vertex – Consultant; FL: Alexion – Consultant; AL: GMP-Orphan and CSL Behring – Consultant; EL: Mirum – Received honoraria; KML: Albireo, Mirum, and Travere Therapeutics – Consultant; CM: Albireo – Consultant; PM: Sobi AB and Albireo – Consultant; EtSo: Cellaion – Chairman and CEO; Albireo – Consultant and investigator; Mirum and Intercept – Investigator; RJS: Mirum, Albireo, and Alexion – Consultant; EkSt: Albireo and Mirum – Consultant and research support; Univar – Consultant; Orphalan – Speaker's fee; HJV: Ausnutria BV, Albireo, Danone/Nutricia Research, Intercept, Mirum, Orphalan, and Vivet – Consultant; JMV: Mirum – Consultant; JPM, QN, TW, NW, QY, PH, and LK: Albireo – current or former employment; RA, PLC, PC, BD, ÖD, GG, WH, HÖ, SRR, BR, EySh, NS, MET, and WLvdW: nothing to disclose.

Please refer to the accompanying ICMJE disclosure forms for further details.
