# Supplementary material for: Interim results from an ongoing, open-label, single-arm trial of odevixibat in progressive familial intrahepatic cholestasis
Source: JHEP Rep. 2023 Apr 29;5(8):100782. doi: 10.1016/j.jhepr.2023.100782 (PMC10338319; doi:10.1016/j.jhepr.2023.100782)
Supplement: Multimedia component 1 [file mmc1.pdf]

Supplementary material

Interim results from an ongoing, open-label, single-arm trial of odevixibat in progressive familial intrahepatic cholestasis

Richard J. Thompson, Reha Artan, Ulrich Baumann, Pier Luigi Calvo, Piotr Czubkowski, Buket Dalgic, Lorenzo D'Antiga, Angelo Di Giorgio, Özlem Durmaz, Emmanuel Gonzalès, Tassos Grammatikopoulos, Girish Gupte, Winita Hardikar, Roderick H.J. Houwen, Binita M. Kamath, Saul J. Karpen, Florence Lacaille, Alain Lachaux, Elke Lainka, Kathleen M. Loomes, Cara L. Mack, Jan P. Mattsson, Patrick McKiernan, Quanhong Ni, Hasan Özen, Sanjay R. Rajwal, Bertrand Roquelaure, Eyal Shteyer, Etienne Sokal, Ronald J. Sokol, Nisreen Soufi, Ekkehard Sturm, Mary Elizabeth Tessier, Wendy L. van der Woerd, Henkjan J. Verkade, Jennifer M. Vittorio, Terese Wallefors, Natalie Warholic, Qifeng Yu, Patrick Horn, Lise Kjems

TABLE OF CONTENTS

Supplementary Methods……………………………………………………………2

Supplementary Results……………………………………………………………5

Supplementary Figures……………………………………………………………8

Supplementary Tables……………………………………………………………10

References……………………………………………………………………17

PEDFIC 2 investigators: Reha Artan, Ulrich Baumann, Pier Luigi Calvo, Piotr Czubkowski, Buket Dalgic, Lorenzo D'Antiga, Angelo Di Giorgio, Özlem Durmaz, Emmanuel Gonzalès, Tassos Grammatikopoulos, Girish Gupte, Winita Hardikar, Roderick H.J. Houwen, Binita M. Kamath, Saul J. Karpen, Florence Lacaille, Alain Lachaux, Elke Lainka, Kathleen M. Loomes, Cara L. Mack, Patrick McKiernan, Hasan Özen, Sanjay R. Rajwal, Bertrand Roquelaure, Eyal Shteyer, Etienne Sokal, Nisreen Soufi, Ekkehard Sturm, Mary Elizabeth Tessier, Richard J. Thompson, Wendy L. van der Woerd, Henkjan J. Verkade, and Jennifer M. Vittorio

Supplementary Methods

The PEDFIC 2 study initiated in September 2018, and 33 sites in North America, Europe, the Middle East, and Australia enrolled patients as of the 15 July 2020 data cutoff date.

Additional details: outcomes and assessments

Because there was a paucity of publicly available instruments for assessing symptoms and impacts of PFIC from the perspective of pediatric patients and/or their caregivers, the PRUCISION instrument was developed. Initial steps of development included review of the literature, discussion with expert clinicians, and interviews with pediatric patients with cholestatic liver diseases and their caregivers.[1] The final PRUCISION instrument is based on caregiver report (an observer-reported outcome [ObsRO]) or patient report (a patient-reported outcome [PRO]) and focuses on key symptoms of pruritus and sleep disturbance.[1]

Caregivers/patients used PRUCISION twice daily in an eDiary format. Morning (ie, AM) assessments captured details on patient scratching/itching and sleep during the previous night, and evening (ie, PM) assessments captured these details experienced during the day.[1] The ObsRO pruritus portion of this instrument was validated by an independent group using psychometric analyses of PEDFIC 1 data, which included measures of test-retest reliability, construct validity, and sensitivity to change.[2] Based on these analyses, ≥1-point decrease in ObsRO pruritus score was deemed clinically meaningful.[2] This threshold for meaningful change was determined prior to any unblinding in PEDFIC 1.

Change in growth was assessed using linear growth compared with a standard growth curve (ie, Z score). Sleep parameters included the percentage of days patients needed help falling asleep, needed soothing, or slept with their caregiver. Cholestasis and liver disease assessments included serum alanine aminotransferase (ALT), aspartate aminotransferase (AST), and total bilirubin levels, AST-to-platelet ratio index (APRI) score, Fibrosis-4 (FIB-4) score, and Pediatric End-stage Liver Disease (PELD) or Model for End-stage Liver Disease (MELD) scores.

The following AEs of interest were summarized: 1) new or worsening fat-soluble vitamin deficiency refractory to clinically recommended vitamin supplementation; 2) clinically significant diarrhea (ie, diarrhea with duration ≥21 days without other etiology; diarrhea of severe intensity or reported as a serious AE; or diarrhea with concurrent dehydration requiring treatment intervention); and 3) hepatic events, including cases that underwent

adjudication by the Data and Safety Monitoring Board such as potential drug-induced liver injury events, suspected liver decompensation events, and events in the Standardized Medical Dictionary for Regulatory Activities Query of *Drug Related Hepatic Disorders – Severe Events Only*.

Additional details: data analysis

There were no imputations for missing data. Any assessments after intercurrent events (eg, initiation of rescue treatments such as biliary diversion surgery or liver transplantation) or follow-up assessments were excluded from analysis.

Change from baseline in serum bile acids was summarized at the end of the 24-week treatment period based on the average of the values at weeks 22 and 24.
For cohort 1, two definitions of baseline were used: PEDFIC 1 baseline refers to the last value prior to the initiation of treatment in PEDFIC 1; PEDFIC 2 baseline is the last value prior to the first dose of odevixibat in PEDFIC 2. For cohort 2, baseline refers to the value prior to initiation of odevixibat in PEDFIC 2.

There was no formal hypothesis testing in this open-label study. The sample size for cohort 1 was determined based on rollover of patients from PEDFIC 1; a sample size of 60 for cohort 2 was estimated based on availability of a target patient population to evaluate the therapeutic benefit for those patients.

Additional details: post hoc analyses of patients who escalated odevixibat dose from 40 μg/kg/day in PEDFIC 1 to 120 μg/kg/day in PEDFIC 2

Patients who were treated with 40 μg/kg/day in PEDFIC 1 and enrolled in PEDFIC 2, where they transitioned to 120 μg/kg/day, were evaluated for treatment response to odevixibat in post hoc analyses. Treatment response was defined as either: 1) a ≥1-point decrease from baseline in ObsRO monthly pruritus score (ie, a pruritus response), or 2) a ≥70% reduction in serum bile acids from baseline or reaching a level ≤70 μmol/L (ie, a serum bile acid response).

Supplementary Results

Additional disposition details for patients entering from PEDFIC 1

There were 62 patients randomized in PEDFIC 1. Of these, 60 were eligible to enroll in PEDFIC 2 (ie, had completed the 24-week treatment period or rolled over early due to intolerable symptoms per protocol), and 54 of these 60 patients enrolled in PEDFIC 2. There were 3 patients from the site in Saudi Arabia who completed PEDFIC 1 but could not enroll in PEDFIC 2 as the study had not opened in that country.

Primary efficacy outcomes by prior odevixibat dose in PEDFIC 1

For patients in cohort 1A, further reductions in serum bile acids were observed during PEDFIC 2 regardless of prior odevixibat dose in PEDFIC 1 (which was either 40 μg/kg/day [ie, odevixibat 40→120 μg/kg/day] or 120 μg/kg/day [ie, odevixibat 120→120 μg/kg/day]). Mean change in serum bile acids from PEDFIC 2 baseline to PEDFIC 2 weeks 22–24 was –13 μmol/L (a mean decrease of 6%) in odevixibat

40→120 μg/kg/day patients and –24 μmol/L (a mean decrease of 15%) in odevixibat 120→120 μg/kg/day patients. Serum bile acid changes over a cumulative treatment period of 48 weeks (ie, PEDFIC 1 baseline to PEDFIC 2 week 24) are shown in Supplementary Table 2.

The mean proportion of positive pruritus assessments in PEDFIC 2 for those in the odevixibat 40→120 μg/kg/day group was 37% and in the odevixibat 120→120 μg/kg/day group was 27%. For patients in either group, continued decreases in pruritus scores were observed through week 24 of PEDFIC 2 (–0.6 for odevixibat 40→120 μg/kg/day and –0.4 for odevixibat 120→120 μg/kg/day). Mean pruritus scores over a cumulative treatment period of 48 weeks (ie, PEDFIC 1 baseline to PEDFIC 2 week 24) are shown in Supplementary Table 2.

Outcomes from patients who escalated from odevixibat 40 μg/kg/day in PEDFIC 1 to 120 μg/kg/day in PEDFIC 2

Of 20 patients who received odevixibat 40 μg/kg/day during PEDFIC 1 and continued into PEDFIC 2, eleven met pruritus responder criteria while receiving odevixibat 40 μg/kg/day during PEDFIC 1 and 9 did not (Supplementary Table 4). Of these 9 patients, 4 (44%) met criteria for pruritus response after 12 weeks of receiving odevixibat 120 μg/kg/day in PEDFIC 2 (Supplementary Table 4). By week 24 of PEDFIC 2, three of 8 (38%) patients who were pruritus non-responders with odevixibat 40 μg/kg/day in PEDFIC 1 became pruritus responders with 120 μg/kg/day (Supplementary Table 4). These data indicate that approximately 40% of patients who

were pruritus non-responders while receiving 40 μg/kg/day became pruritus responders when the dose was increased to 120 μg/kg/day. Importantly, among the patients who were pruritus responders on 40 μg/kg/day in PEDFIC 1 and went on to receive 120 μg/kg/day in PEDFIC 2, all 8 with available data at week 12 remained responders, as did all 7 with available data at week 24 (Supplementary Table 4).

Of 20 patients who received odevixibat 40 μg/kg/day during PEDFIC 1 and continued into PEDFIC 2, eleven met serum bile acid responder criteria while receiving odevixibat 40 μg/kg/day during PEDFIC 1 and 9 did not; data are available for 6 patients who did not meet the serum bile acid responder definition while on 40 μg/kg/day during PEDFIC 1 (Supplementary Table 4). In PEDFIC 2, 12 weeks after switching to odevixibat 120 μg/kg/day, all of these 6 patients remained serum bile acid non-responders, and for 4 of these patients with available data at week 24 of PEDFIC 2, one (25%) met the serum bile acid responder definition (Supplementary Table 4).

SUPPLEMENTARY FIGURES

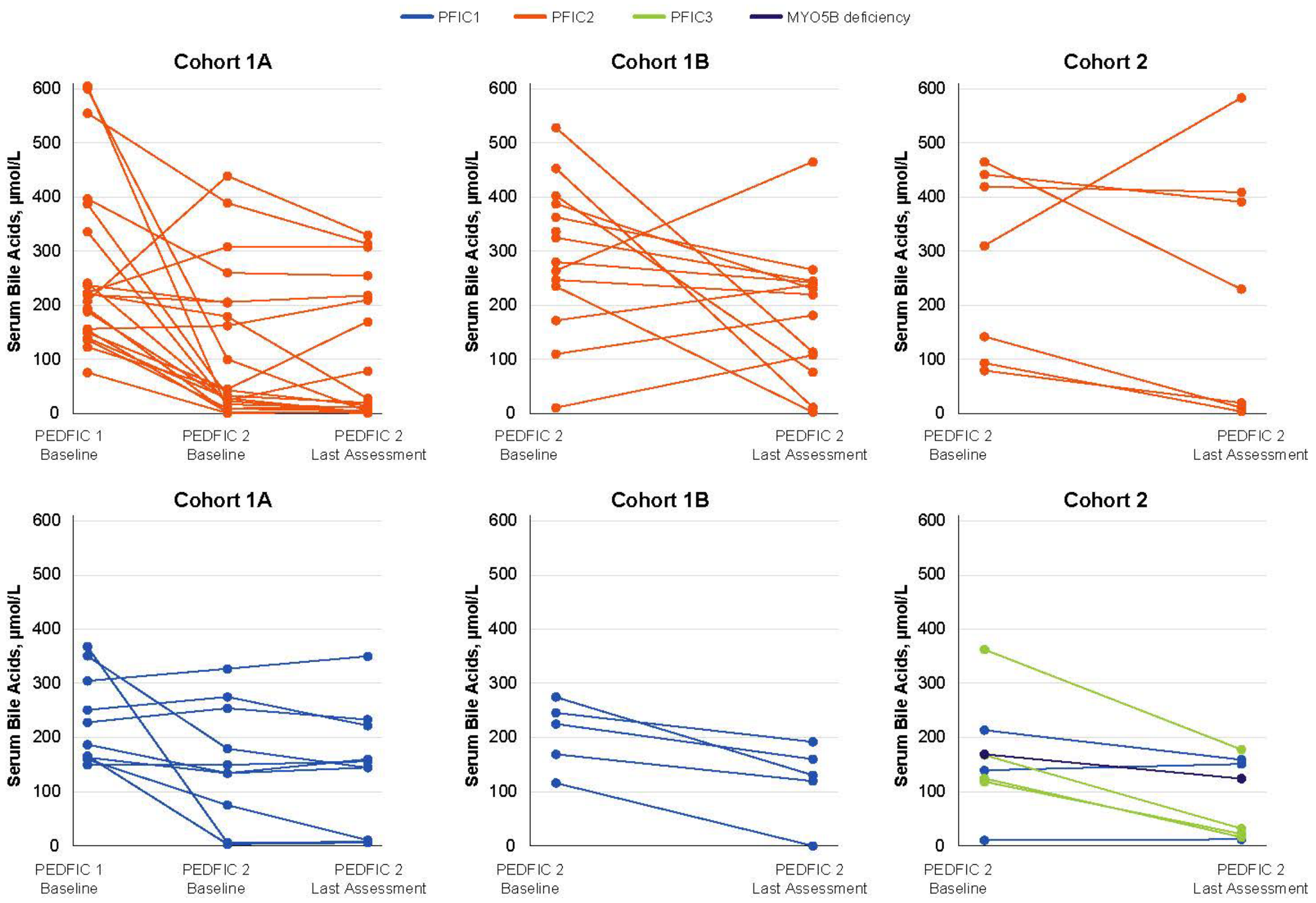

Fig. S1: Change in serum bile acids in individual patients by PFIC type from before first dose of odevixibat through last available assessment in PEDFIC 2 up to week 24

PFIC=progressive familial intrahepatic cholestasis.

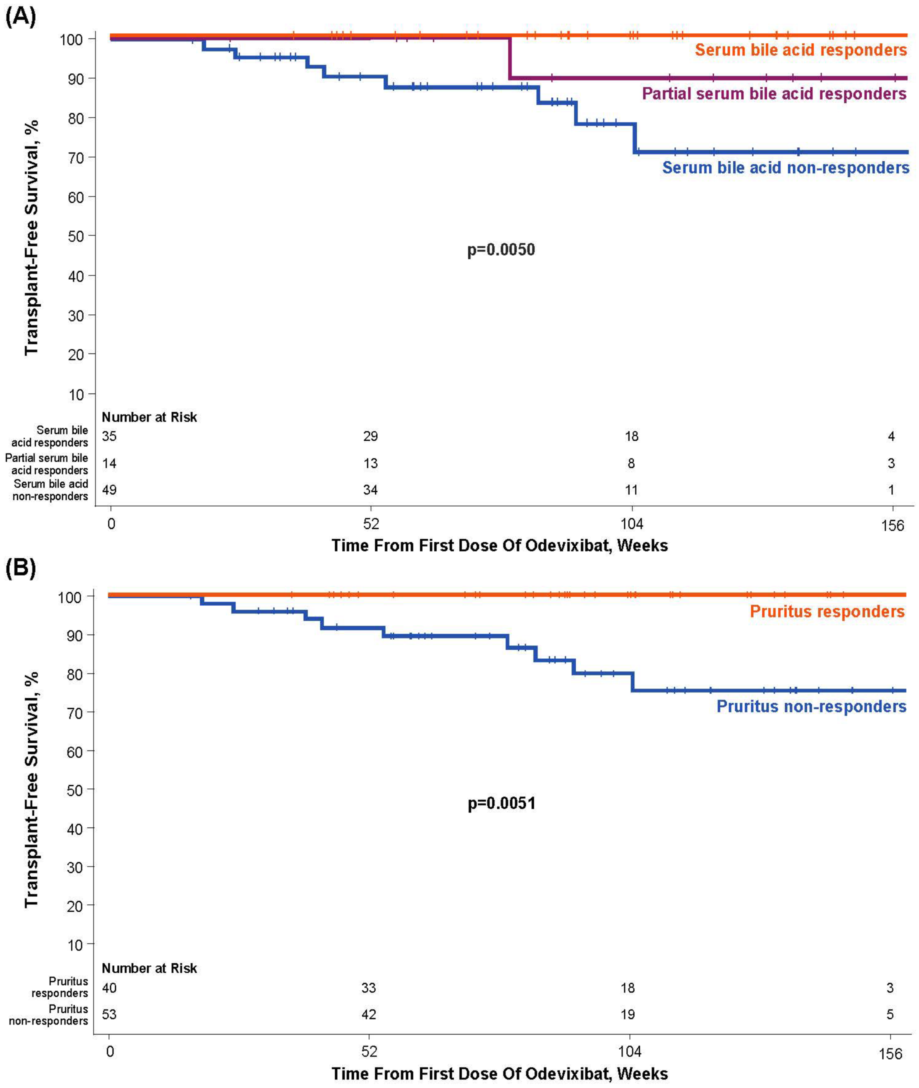

Fig. S2: Native liver survival by serum bile acid response (A) or pruritus response (B) category in odevixibat-treated patients

Native liver survival was analyzed in odevixibat-treated patients in an ad hoc supplementary analysis of pooled data from PEDFIC 1 and PEDFIC 2 to a data cutoff date of 31 January 2022. Serum bile acid response was defined as ≥70% reduction from baseline or levels ≤70 μmol/L at month 6, serum bile acid partial response was defined as ≥30% to <70% reduction at month 6, and serum bile acid non-response was defined as <30% reduction, liver transplant, or treatment discontinuation before month 6. Pruritus response was defined as a ≥1-point drop in monthly pruritus score from baseline to month 6. Of 98 patients analysed, 35 (36%) were serum bile acid responders, 14 (14%) were partial serum bile acid responders, and 49 (50%) were non-responders. All 35 serum bile acid responders and 13 of the 14 partial serum bile acid responders remained transplant free; 8 of the 49 non-responders underwent liver transplantation. Overall, a decrease in serum bile acids at 6 months was strongly associated with native liver survival in patients with PFIC, and all patients with a pruritus response at 6 months remained transplant free. p value is based on log-rank test for responders vs non-responders. +, Censored. PFIC=progressive familial intrahepatic cholestasis.

SUPPLEMENTARY TABLES

Table S1: Names of site-specific boards/institutions that approved the study protocol

The Royal Children's Hospital, Research Ethics & Governance (LEC); Melbourne, Australia
Comité d'Ethique Hospitalo-Facultaire, SaintLuc UCL (EC); Brussels, Belgium
SickKids Research Ethics Board (LEC); Toronto, Canada
East II Ethics Committee; Regional University Hospital Center – Saint Jacques Hospital (CEC); Besançon, France
Ethikkommission Medizinische Fakultät der Universität Duisburg-Essen (LEC); Essen, Germany
Ethikkommission der Medizinischen Hochschule Hannover (LEC); Hannover, Germany
Ethikkommission an der Medizinischen Fakultät der Eberhard-Karls-Universität und am Universitätsklinikum Tübingen (CEC); Tübingen, Germany
Helsinki Committee, Shaare Zedek Medical Center (LEC); Jerusalem, Israel
Helsinki Committee, Rabin Medical Center (LEC); Petah Tikva, Israel
Comitato Etico della provincia di Bergamo, ASST Papa Giovanni XXIII (CEC); Bergamo, Italy
Comitato Etico per la Sperimentazione Clinica della Provincia di Padova, Azienda Ospedaliera di Padova (LEC); Padova, Italy
Comitato Etico Interaziendale, A.O.U. Città della Salute e della Scienza di Torino (LEC); Torino, Italy
MREC Brabant (CEC); Tilburg, the Netherlands
Komisja Bioetyczna przy Instytucie "Pomnik-Centrum Zdrowia Dziecka" (CEC); Warsaw, Poland
King Faisal Specialist Hospital and Research Center, Research Advisory Council; Riyadh, Saudi Arabia
CEIC Hospital Universitario Vall de Hebron, Hospital Materno-Infantil (CEC); Barcelona, Spain
Regionala Etikprövningsnämnden i Stockholm, Karolinska Institutet (CEC); Solna, Sweden
Akdeniz University Medical Faculty, Clinical Research Studies Ethics Committee (CEC); Antalya, Turkey
London – Dulwich Research Ethics Committee, Health Research Authority (CEC); London, United Kingdom

Children's Hospital of Los Angeles Institutional Review Board (LEC); Los Angeles, California, United States
Committee on Human Research; San Francisco, California, United States
Emory University Institutional Review Board (LEC); Atlanta, Georgia, United States
Johns Hopkins Medical Institutions Office of Human Subjects Research Institutional Review Boards (LEC); Baltimore, Maryland, United States
The Washington University in St. Louis Institutional Review Board (LEC); St. Louis, Missouri, United States
Columbia University Medical Center Institutional Review Board; New York, New York, United States
Institutional Review Board of the Mount Sinai School of Medicine (LEC); New York, New York, United States
Cleveland Clinic Institutional Review Board (LEC); Cleveland, Ohio, United States
The Committees for Protection of Human Subjects/Institutional Review Board Research Regulatory Affairs (LEC); Philadelphia, Pennsylvania, United States
WCB Institutional Review Board (LEC); Puyallup, Washington, United States

LEC=local ethics committee. EC=ethics committee. CEC=central ethics committee.

Table S2. Changes in serum bile acids and pruritus scores in cohort 1A patients by prior odevixibat dose

	Odevixibat 40→ 120 μg/kg/day			Odevixibat 120→ 120 μg/kg/day		
	**n**	**Mean (SE)**	**Range**	**n**	**Mean (SE)**	**Range**
**Serum bile acids**						
PEDFIC 1 baseline	19	251 (28)	76, 605	15	253 (40)	116, 600
PEDFIC 2 weeks 22–24	12	79 (31)	2, 255	9	93 (44)	3, 314
Cumulative change from PEDFIC 1 baseline to PEDFIC 2 week 22–24	12	–193 (50)	–600, 20	9	–211 (62)	–596, 84
**Pruritus scores**						
PEDFIC 1 baseline	19	3.0 (0.1)	2, 4	15	2.9 (0.1)	2.2, 3.4
PEDFIC 2 week 21–24	15	1.5 (0.3)	0, 4	11	1.2 (0.4)	0, 4
Cumulative change from PEDFIC 1 baseline to PEDFIC 2 week 22–24	15	–1.4 (0.3)	–3.8, 0.9	11	–1.7 (0.4)	–3.1, 1.1

Table S3: Serum bile acid and pruritus responders from the start of odevixibat treatment and through PEDFIC 2 week 24

		Proportion of patients meeting response criteria, n/m (%)[a]				
		≥1-point reduction in pruritus score	Serum bile acid level <65 μmol/L (PFIC1) or <102 μmol/L (PFIC2)		Serum bile acid level ≤70 μmol/L or reduction of ≥70% from baseline	
	**Cohort**	**Pruritus response**	**Serum bile acid response**	**Serum bile acid and/or pruritus response**	**Serum bile acid response**	**Serum bile acid and/or pruritus response**
Start of odevixibat[b] to PEDFIC 2 week 24	Cohort 1A	23/34 (68)	18/33 (55)	24/34 (71)	18/34 (53)	24/34 (71)
	Cohort 1B + cohort 2	19/33 (58)	6/22 (27)	20/34 (59)	11/31 (36)	21/34 (62)

[a]At last available assessment in interval.
[b]For patients in cohort 1A, this is PEDFIC 1 baseline; patients in this cohort had up to 48 weeks of cumulative odevixibat exposure; for patients in cohort 1B and cohort 2, this is PEDFIC 2 baseline, and patients in these cohorts had up to 24 weeks of cumulative odevixibat exposure.
PFIC=progressive familial intrahepatic cholestasis.

Table S4. Proportions of patients receiving odevixibat 40 μg/kg/day in PEDFIC 1 and 120 μg/kg/day in PEDFIC 2 meeting criteria for pruritus or serum bile acid response

**Pruritus Response**				
	**Responders on 40 μg/kg/day[a] (n=11)**		**Non-responders on 40 μg/kg/day[b] (n=9)**	
**Visit**	Responder on 120 μg/kg/day n/N (%)	Non-responder on 120 μg/kg/day n/N (%)	Responder on 120 μg/kg/day n/N (%)	Non-responder on 120 μg/kg/day n/N (%)
PEDFIC 2 Weeks 9−12	8/8 (100)	0/8 (0)	4/9 (44)	5/9 (56)
PEDFIC 2 Weeks 21−24	7/7 (100)	0/7 (0)	3/8 (38)	5/8 (63)
**Serum Bile Acid Response**				
	**Responders on 40 μg/kg/day[c] (n=11)**		**Non-responders on 40 μg/kg/day[d] (n=9)**	
**Visit**	Responder on 120 μg/kg/day n/N (%)	Non-responder on 120 μg/kg/day n/N (%)	Responder on 120 μg/kg/day n/N (%)	Non-responder on 120 μg/kg/day n/N (%)
PEDFIC 2 Week 12	7/9 (78)	2/9 (22)	0/6 (0)	6/6 (100)
PEDFIC 2 Weeks 22/24	7/8 (88)	1/8 (13)	1/4 (25)	3/4 (75)

[a]Achieved ≥1-point reduction from baseline in pruritus score on the ObsRO instrument during PEDFIC 1; [b]Did not achieve a 1-point reduction from baseline in pruritus score on the ObsRO instrument during PEDFIC 1; [c]Achieved ≥70% reduction in serum bile acids from baseline or reached a level of ≤70 μmol/L during PEDFIC 1 (for patients who completed PEDFIC 1, the average serum bile acid value from weeks 22 and 24 was used to determine serum bile acid response; for patients who rolled over to PEDFIC 2 early, the serum bile acid value at week 12 was used); [d]Did not achieve ≥70% reduction in serum bile acids from baseline nor reach a level of ≤70 μmol/L during PEDFIC 1. ObsRO=observer-reported outcome.

Table S5. Effects of odevixibat on markers of liver disease through week 24 of PEDFIC 2

	Cohort 1				Cohort 2	
	Cohort 1A		Cohort 1B			
	n	Mean (SE)	n	Mean (SE)	n	Mean (SE)
**APRI**						
PEDFIC 1 baseline	42[a]	0.6 (0.1)	20[b]	0.5 (0.1)	NA	
PEDFIC 2 baseline	33	0.5 (0.1)	19	0.5 (0.1)	15	1.0 (0.3)
Change to week 12	23	0.2 (0.2)	13	0.2 (0.1)	9	1.9 (0.9)
Change to week 24	18	–0.0 (0.1)	9	0.1 (0.1)	4	0.1 (0.2)
**FIB-4**						
PEDFIC 1 baseline	42[a]	0.14 (0.03)	20[b]	0.14 (0.04)	NA	
PEDFIC 2 baseline	33	0.14 (0.02)	19	0.14 (0.04)	15	0.45 (0.12)
Change to week 12	23	0.07 (0.05)	13	0.04 (0.01)	9	0.38 (0.18)
Change to week 24	18	0.03 (0.01)	9	0.02 (0.01)	4	0.04 (0.01)
**PELD/MELD**						
PEDFIC 1 baseline	42[a]	−1.5 (1.1)	20[b]	−0.8 (1.7)	NA	
PEDFIC 2 baseline	34	–4.0 (1.3)	19	–1.5 (1.9)	16	0.4 (1.7)
Change to week 12	24	0.7 (0.4)	16	0.9 (1.3)	11	1.1 (1.2)
Change to week 24	17	–0.2 (0.3)	9	–1.4 (1.2)	2	1.7 (5.8)

[a]All patients who received odevixibat in PEDFIC 1; [b]All patients who received placebo in PEDFIC 1. APRI=aspartate aminotransferase-to-platelet ratio index. NA=not applicable. FIB-4=fibrosis-4 score. PELD/MELD=pediatric end-stage liver disease/model for end-stage liver disease.

Table S6. Changes from baseline in fat-soluble vitamins

	Cohort 1				Cohort 2	
	Cohort 1A		Cohort 1B			
	n	Mean (SD)	n	Mean (SD)	n	Mean (SD)
**Vitamin A (μmol/L)**						
PEDFIC 2 baseline	34	0.3 (0.7)	19	0.1 (0.1)	16	0.2 (0.2)
Change to last assessment	28	3.6 (19)	16	0.0 (0.2)	14	–0.1 (0.2)
**Vitamin D[a] (nmol/L)**						
PEDFIC 2 baseline	34	78 (56)	19	74 (55)	16	52 (32)
Change to last assessment	28	11 (56)	16	12 (46)	14	8.8 (25)
**Vitamin E[b] (μmol/L)**						
PEDFIC 2 baseline	34	13 (7.6)	19	9.0 (6.0)	16	15 (7.1)
Change to last assessment	28	2.6 (9.8)	16	1.7 (5.4)	14	–1.7 (6.9)

[a]25-Hydroxyvitamin D; [b]Reported as alpha tocopherol.

REFERENCES

[1] Gwaltney C, Bean S, Venerus M, Karlsson K, Warholic N, Kjems L, et al. Development of the patient- and observer-reported PRUCISION instruments to assess pruritus and sleep disturbance in pediatric patients with cholestatic liver diseases Adv Ther 2022;39:5126-5143.

[2] Gwaltney C, Ivanescu C, Karlsson L, Warholic N, Kjems L, Horn P. Validation of the PRUCISION instruments in pediatric patients with progressive familial intrahepatic cholestasis. Adv Ther 2022;39:5105-5125.
