# Supplementary material for: Interim results from an ongoing, open-label, single-arm trial of odevixibat in progressive familial intrahepatic cholestasis
Source: JHEP Rep. 2023 Apr 29;5(8):100782. doi: 10.1016/j.jhepr.2023.100782 (PMC10338319; doi:10.1016/j.jhepr.2023.100782)
Supplement: Multimedia component 2 [file mmc2.docx]

**Complete, Transparent, Accurate, and Timely Account Table**

***Biological samples***

| **Description** | **Source** | **Identifier** |
| --- | --- | --- |
| Blood and urine samples | Patients who received study drug in PEDFIC 2 | Each patient was assigned a unique 8-digit code that remained associated with each patient’s samples |

***Software***

| **Software name** | **Manufacturer** | **Version** |
| --- | --- | --- |
| SAS | SAS Institute | 9.4 or higher |

***Other (e.g. drugs, proteins, vectors etc.)***

| Odevixibat | 120 µg/kg/day |  |
| --- | --- | --- |

***Please provide the details of the corresponding methods author for the manuscript:***

| Jan P. Mattsson  Albireo AB Arvid Wallgrens backe 20, 413 46 Göteborg, Sweden Phone: +46 31 741 14 80 |
| --- |
