# Supplementary material for: Interim results from an ongoing, open-label, single-arm trial of odevixibat in progressive familial intrahepatic cholestasis
Source: JHEP Rep. 2023 Apr 29;5(8):100782. doi: 10.1016/j.jhepr.2023.100782 (PMC10338319; doi:10.1016/j.jhepr.2023.100782)
Supplement: Multimedia component 4 [file mmc4.zip › Clinical Trial/a4250-008-protocol-amendment-5_redaction applied_Redacted.pdf]

CLINICAL STUDY PROTOCOL A4250-008

An Open-label Extension Study to Evaluate Long-term Efficacy and Safety of A4250 in Children with Progressive Familial Intrahepatic Cholestasis Types 1 and 2 (PEDFIC 2)

IND Number:	130591
EudraCT Number	2017-002325-38
Text Product:	A4250
Indication:	Progressive Familial Intrahepatic Cholestasis (PFIC)
Sponsor:	Albireo AB
Development Phase:	Phase 3
[redacted]	[redacted]
[redacted]	[redacted]
[redacted]	[redacted]
Date of the Protocol:	18 March 2020
Version of the Protocol:	Amendment 05

Confidential

The information in this document contains trade secrets and commercial information that are privileged or confidential and may not be used, divulged, published, or otherwise disclosed without the written consent of Albireo AB. These restrictions on disclosure apply equally to all future information supplied to you or generated by you in connection with the study.

SPONSOR SIGNATURE PAGE

PROTOCOL TITLE: An Open-label Extension Study to Evaluate Long-term Efficacy and Safety of A4250 in Children with Progressive Familial Intrahepatic Cholestasis Types 1 and 2 (PEDFIC 2)

PROTOCOL NUMBER: A4250-008

Albireo AB

[redacted]

[redacted]

[redacted]

Date (day/month/year)

INVESTIGATOR SIGNATURE PAGE

PROTOCOL TITLE: An Open-label Extension Study to Evaluate Long-term Efficacy and Safety of A4250 in Children with Progressive Familial Intrahepatic Cholestasis Types 1 and 2 (PEDFIC 2)

PROTOCOL NUMBER: A4250-008

I have read this protocol and agree that it contains all necessary details for performing this study. I will conduct the study as outlined herein and will complete the study within the time designated, in accordance with all stipulations of the protocol and in accordance with Good Clinical Practice (GCP), local regulatory requirements, and the Declaration of Helsinki.

I will provide copies of the protocol and all pertinent information to all individuals responsible to me who assist in the conduct of this study. I will discuss this material with them to ensure that they are fully informed regarding the study drug and the conduct of the study.

I will use only the informed consent approved by the Independent Ethics Committee (IEC) and will fulfil all responsibilities for submitting pertinent information to the IEC responsible for this study.

I agree that the Sponsor (Albireo AB) shall have access to any source documents from which case report form information may have been generated.

I further agree not to originate or use the name of Albireo AB or A4250 in any publicity, news release, or other public announcement, written or oral, whether to the public, press, or otherwise, relating to this protocol, to any amendment to the protocol, or to the performance of this protocol without the prior written consent of Albireo AB.

____

Name of Investigator

____ _____________________________________

Signature Date (day/month/year)

1 ADMINISTRATIVE INFORMATION

An Open-label Extension Study to Evaluate Long-term Efficacy and Safety of A4250 in Children with Progressive Familial Intrahepatic Cholestasis Types 1 and 2 (PEDFIC 2)

Protocol No.:	A4250-008
Date of the initial Protocol:	24 August 2017
Date and Number of Amendment(s):	Amendment 01, 18 April 2018<br>Amendment 02, 18 January 2019<br>Amendment 03, 11 March 2019<br>Amendment 04, 25 October 2019<br>Amendment 05, 18 March 2020
Sponsor:	Albireo AB<br>Arvid Wallgrens Backe 20<br>413 46 Göteborg<br>Sweden
[redacted]	[redacted]
[redacted]	
[redacted]	
[redacted]	

2 STUDY SYNOPSIS

**Name of Sponsor/Company:**<br>Albireo AB	**Name of Product:**<br>A4250	**Name of Active Ingredient:**<br>A4250

Title of Study:
An Open-label Extension Study to Evaluate Long-term Efficacy and Safety of A4250 in Children with Progressive Familial Intrahepatic Cholestasis Types 1 and 2 (PEDFIC 2)

Principal Investigator:
[redacted]

Study Centers:
Up to 50 sites will be included in this study in the United States (US), Canada, Europe, Australia, and Middle East.

Publication(s):
None.

**Planned Study Period:**<br>Q3 2018 to Q3 2021	**Development Phase:**<br>Phase 3

Objectives:

Primary Objective (Cohort 1)

To demonstrate a sustained effect of A4250 on serum bile acids (s-BAs) and pruritus in children with progressive familial intrahepatic cholestasis (PFIC) Types 1 and 2.

Primary Objective (Cohort 2)

To evaluate the effect of A4250 on s-BAs and pruritus in patients with PFIC who either (1) do not meet eligibility criteria for Study A4250-005 (PEDFIC 1) or (2) patients who do meet the eligibility criteria for Study A4250-005 after recruitment of Study A4250-005 has been completed.

Secondary Objectives (Cohorts 1 and 2)

- To evaluate the long-term safety and tolerability of repeated daily doses of A4250
- To evaluate the effect of A4250 on growth
- To evaluate the effect of A4250 on biliary diversion and/or liver transplantation
- To evaluate the effect of A4250 on biochemical markers of cholestasis and liver disease

Methodology:

This is a Phase 3, multi-center, open-label extension study to investigate the long-term efficacy and safety of a 120 µg/kg/day daily dose of A4250 in patients with PFIC. Cohort 1 will consist of children with PFIC Types 1 and 2 who have participated in study A4250-005. Cohort 2 will consist of approximately 60 patients with PFIC who have elevated s-BAs and cholestatic pruritus and who either (1) do not meet eligibility criteria for Study A4250-005 (PEDFIC 1) or (2) are eligible for enrolment in A4250-005 after recruitment of study A4250-005 has been completed. Up to 40 patients post biliary diversion surgery are allowed to participate in Cohort 2.

Eligible patients will be enrolled into this open-label extension study and treated with a daily dose of 120 µg/kg/day of A4250 for 72 weeks. Patients not tolerating the 120 µg/kg/day dose after a minimum of 1 week will have the option to down-titrate to a lower dose (40 µg/kg/day). The patient should return to the higher dose as soon as deemed appropriate by the investigator. More than one upward dose titration (from 40 µg/kg/day directly to 120 µg/kg/day) for the same event is not recommended. Any dose titration should be done in consultation with the sponsor Medical Monitor or designee.

Patients who wish to continue receiving A4250 after 72 weeks, will have the option to remain on treatment until the drug is commercially available, provided continued use is supported by the risk-benefit profile and the subject has not been previously withdawn or discontinued from the study.

The primary analysis will be performed after the last patient (from Cohort 1 or 2) completes the 72-week treatment period. Analyses during the extension period will consist of safety summaries and other evaluations on an ongoing basis per the schedule of assessment for the extension period.

**Name of Sponsor/Company:** Albireo AB	**Name of Product:** A4250	**Name of Active Ingredient:** A4250

Number of Patients:

Approximately 120 patients in total. Approximately 60 patients will be enrolled following participation in Study A4250-005 (Cohort 1) and approximately 60 patients will be enrolled directly into this study (Cohort 2).

Diagnosis and Main Criteria for Inclusion:

Cohort 1:

1. Completion of the 24-week Treatment Period of Study A4250-005 or withdrawn from Study A4250-005 due to patient/caregiver judgment of intolerable symptoms after completing at least 12 weeks of treatment. Patients who withdraw from A4250-005 due to a study drug related adverse event (AE) will not be eligible
2. Signed informed consent and assent as appropriate. Patients who turn 18 years of age (or legal age per country) during the study will be required to re-consent to remain on the study
3. Patients expected to have a consistent caregiver for the duration of the study
4. Caregivers (and age appropriate patients) must be willing and able to use an electronic diary (eDiary) device as required by the study

***Cohort 2*:**

1. A male or female patient of any age, with a clinical diagnosis of PFIC and with a body weight ≥5 kg at Visit S-1
2. Patient must have clinical genetic confirmation of PFIC
3. Patient must have elevated s-BA concentration, specifically measured to be ≥100 µmol/L, taken as the average of 2 samples at least 7 days apart (Visits S-1 and S-2) prior to the Screening/Inclusion Visit (Visit 1)
4. Patient must have history of significant pruritus and a caregiver-reported observed scratching or patient-reported itching (for patients >18 with no caregiver-reported observed scratching) in the eDiary average of ≥2 (on 0 to 4 scale) in the 2 weeks prior to the Screening/Inclusion Visit (Visit 1)
5. Patient and/or legal guardian must sign informed consent (and assent) as appropriate. Patients who turn 18 years of age (or legal age per country) during the study will be required to re-consent in order to remain in the study.
6. Age appropriate patients are expected to have a consistent caregiver for the duration of the study
7. Caregivers and age-appropriate patients (≥8 years of age, if able) must be willing and able to use an eDiary device as required by the study

Test Product, Dose and Mode of Administration:

A4250, 120 µg/kg/day orally administered. Patients not tolerating the 120 µg/kg/day dose after a minimum of 1 week will have the option to down-titrate to a lower dose (40 µg/kg/day).

Reference Therapy, Dose and Duration of Administration:

Not applicable.

Duration of Treatment:

72 weeks

Variables:

Efficacy:

Primary Efficacy Endpoints

EU and rest of world: Change from baseline in s-BA after 72 weeks of treatment.

US: Proportion of positive pruritus assessments at the patient level over the 72 week treatment period using the Albireo observer-reported outcome (ObsRO) instrument.

Secondary Efficacy Endpoints

EU and rest of world: Proportion of positive pruritus assessments at the patient level over the 72-week treatment period using the Albireo ObsRO instrument.

US: Change from baseline in s-BA after 72 weeks of treatment.

All Regions:

- Change from baseline in s-BA at Weeks 4, 12, 22, 24, 36, 46, 48, 60, 70, 72, and 76
- Proportion of individual assessments meeting the definition of a positive pruritus assessment at the patient level using the Albireo ObsRO instrument from Weeks 0-4, Weeks 0-12, Weeks 0-22, Weeks 0-24, Weeks 0-36, Weeks 0-46, Weeks 0-48, Weeks 0-60, and Weeks 0-70, respectively, or the proportion of positive pruritus assessments at each 4-week interval between Visit 1/Screening and Visit 5/Week 24, then by each visit between Visit 5/Week 24 to Visit 12/Week 76
- Proportion of individual AM assessments meeting the definition of a positive pruritus assessment at the patient level using the Albireo ObsRO instrument from Weeks 0-4, Weeks 0-12, Weeks 0-22, Weeks 0-24, Weeks 0-36, Weeks 0-46, Weeks 0-48, Weeks 0-60, Weeks 0-70, and Weeks 0-72, respectively, or the proportion of positive pruritus assessments at each 4-week interval between Visit 1/Screening and Visit 5/Week 24, then by each visit between Visit 5/Week 24 to Visit 12/Week 76
- Proportion of individual PM assessments meeting the definition of a positive pruritus assessment at the patient level using the Albireo ObsRO instrument from Weeks 0-4, Weeks 0-12, Weeks 0-22, Weeks 0-24, Weeks 0-36, Weeks 0-46, Weeks 0-48, Weeks 0-60, Weeks 0-70, and Weeks 0-72, respectively, or the proportion of positive pruritus assessments at each 4-week interval between Visit 1/Screening and Visit 5/Week 24, then by each visit between Visit 5/Week 24 to Visit 12/Week 76
- All-cause mortality, number of patients undergoing biliary diversion surgery or liver transplantation. These parameters will be evaluated separately and together at weeks 24, 48, and 72
- Change in growth from baseline to Weeks 24, 48, and 72 after initiation of A4250 treatment, defined as the linear growth deficit (height/length for age, weight for age, and body mass index [BMI]) compared to a standard growth curve (Z-score, standard deviation [SD] from P50)
- Change in aspartate aminotransferase (AST) to platelet ratio index (APRI) score and Fib-4 score from baseline to Week 72
- Change in pediatric end-stage liver disease (PELD)/model for end-stage liver disease (MELD) score from baseline to Week 72
- Change in use of antipruritic medication at Weeks 24, 48, and 72

Exploratory Efficacy Endpoints

All Exploratory Endpoints will be evaluated at Weeks 24, 48, and 72 unless otherwise noted. Baseline is calculated prior to initiation of A4250 treatment. Exploratory endpoints include:

- Change in serum alanine aminotransferase (ALT), gamma-glutamyl transferase, and total bilirubin concentration from baseline to Week 72
- Proportion of individual assessments meeting the definition of a positive pruritus assessment at the patient level over the 72-week Treatment Period. A positive pruritus assessment includes an itch score ≤1, or at least a one-point drop from baseline based on the Albireo patient-reported outcome (PRO) instrument; only patients ≥8 years of age will complete the Albireo PRO instrument
- Change from baseline over the 72-week treatment period in sleep parameters measured with the Albireo PRO and ObsRO instruments
- Change from baseline in international normalized ratio, albumin, liver enzymes, leukocytes, and platelets
- Change from baseline measures of bile acid synthesis (autotaxin, plasma 7α-hydroxy-4-cholesten-3-one [p-C4])

**Name of Sponsor/Company:** Albireo AB	**Name of Product:** A4250	**Name of Active Ingredient:** A4250

- Assessment of Global Symptom Relief at weeks 4, 12, 24, 48 and 72 as measured by patient, caregiver, and clinician global impression of change (PGIC) items
- Change from baseline by each 4-week interval between Visit 1/Screening and Visit 5/Week 24, then by each visit between Visit 5/Week 24 to Visit 12/Week 76 in patient-reported and observer-reported night time itching and scratching severity scores, respectively
- Change from baseline by each 4-week interval between Visit 1/Screening and Visit 5/Week 24, then by each visit between Visit 5/Week 24 to Visit 12/Week 76 in patient-reported and observer-reported morning time itching and scratching severity scores, respectively
- Change from baseline by each 4-week interval between Visit 1/Screening and Visit 5/Week 24, then by each visit between Visit 5/Week 24 to Visit 12/Week 76 in pooled pruritus score including observer-reported scratching for patients <8 years of age and patient-reported itch severity for patients ≥8 years of age
- Change from baseline by each 4-week interval between Visit 1/Screening and Visit 5/Week 24, then by each visit between Visit 5/Week 24 to Visit 12/Week 76 in additional patient-reported and observer-reported sleep parameters (e.g., tiredness, number of awakenings)
- Change from baseline in Pediatric Quality of Life Inventory (PedsQL) questionnaire
- Change from baseline in stage of liver fibrosis as assessed by Fibroscan® (where available)
- Change from baseline in stage of liver fibrosis as assessed by post-treatment biopsy (when available)

Safety:

Safety criteria are as follows:

- Occurrence of treatment-emergent AEs including severity and relatedness to study drug at all visits
- The incidence of treatment-emergent serious adverse events (SAEs), based upon information from patient reports, including the description, causality, and severity of an SAE
- Trends evaluated for the following assessments: physical examinations, concomitant medications, vital signs, laboratory test results (including clinical chemistry, hematology, urinalysis, alfa-fetoprotein, and vitamins A, E, and 25-hydroxy vitamin D), and abdominal ultrasound

Statistical Methods:

Descriptive statistics will mainly be used in this open-label extension study.

Baseline 1 will be defined as the last value prior to treatment start in study A4250-005 for Cohort 1 patients.

Baseline 2 will be defined as the last value prior to treatment start in Study A4250-008 for all patients. Baseline 2 will be used in all analyses unless otherwise specified.

The full analysis set will consist of all patients who received at least 1 dose of study drug in Study A4250-008. The full analysis set will be the primary analysis set for all analyses unless otherwise specified.

Evaluation of Primary Efficacy Variables:

The proportion of positive pruritus assessments at the patient level over the 72-week treatment period will be summarized. A positive pruritus assessment is defined as a scratching score of ≤1 or at least a one-point drop from baseline on the Albireo ObsRO instrument. At each assessment, the AM score will be compared to the baseline AM average, and the PM score will be compared to the baseline PM average. Both AM and PM pruritus assessments will be included in the analysis of this endpoint. Baseline will be defined as the average score from 14 days prior to treatment start in A4250-008 for AM and PM, respectively.

The change from baseline over the treatment period in s-BA will be analyzed mainly by using descriptive statistics. Fasting s-BA baseline will be calculated as the average of the pre-dose value in Study A4250-008 (Visit 1) and the previous visit (Visit 8 in study A4250-005) for Cohort 1. Fasting s-BA baseline will be calculated as the average of Visit S-2 and the pre-dose Visit 1 values in Study A4250-008 for Cohort 2. The end value is the average of the values at Weeks 70 and 72 in study A4250-008.

Descriptive statistics will be summarized for Cohort 1, Cohort 2, and combined Cohort 1 + Cohort 2. Within Cohort 1, the following subgroups will be presented: ‘A4250 40 μg/kg/day (005) to 120 μg/kg/day (008)’, ‘A4250 120 μg/kg/day (005) to 120 μg/kg/day (008)’, ‘A4250 40 μg/kg/day (005) and 120 μg/kg/day (005) to 120 μg/kg/day (008)’, and ‘placebo (005) to 120 μg/kg/day (008)’. Cohort 1 Placebo to A4250

**Name of Sponsor/Company:** Albireo AB	**Name of Product:** A4250	**Name of Active Ingredient:** A4250

120 µg/kg/day will also be combined with Cohort 2 for presentations. Change over time will also be displayed using graphical presentations (e.g., a spaghetti plot which illustrates the change in both Study A4250-005 and Study A4250-008 by treatment group in Study A4250-005).

Evaluation of Secondary and Exploratory Efficacy Variables:

All secondary and exploratory variables will be analyzed descriptively for categorical and continuous data, as applicable. For continuous data, the change from baseline will be analyzed in addition to the actual visit values. For categorical data, shift tables or frequency and percentages of patients will be presented as appropriate.

Change in growth (Z-score, SD from P50 standard growth curve) will be analyzed by using one-sample T-test or Wilcoxon signed rank test as appropriate. Change in growth (height/length for age, weight for age, and BMI) will also be displayed using graphical presentations.

A 95% confidence interval (CI) will be calculated for the proportion of patients with at least one event, i.e., bile diversion or liver transplantation or death. The upper boundary will be compared to the expected proportion of events, using the event probability estimated from the untreated reference population in an ongoing observational cohort study.

Time to first event, i.e. time to surgical bile diversion or liver transplantation or death, will be explored by descriptive statistics and Kaplan-Meier curves, which also will be used to make a comparison to the untreated reference population.

Evaluation of Safety Variables:

Safety data will be analyzed using descriptive statistics and summaries overall of SAEs, AEs, vital signs, clinical laboratory tests (hematology, clinical chemistry and urinalysis) and concomitant medication. Analyses will be performed using the full analysis set.

Sample Size Determination:

There is no formal hypothesis testing in this open label-study. Sample size for Cohort 1 was determined in Study A4250-005. For Cohort 1, patients will be enrolled following completion or participation of 12 weeks of Study A4250-005. The expected proportion of patients with at least one event, i.e., surgical bile diversion or liver transplantation or death, in untreated patients is expected to be high enough to be able to detect an improvement after A4250 treatment. If the expected proportion with at least one event is estimated to be 30% in the study population of Cohort 1, and then if the proportion of patients with at least one event during A4250 long-term treatment are shown to be only 10% in the study, the power is approximately 89% to 96% (1-sided test, alpha=2.5%) for a CI with upper boundary <30% based on a sample size of n=48 to 60. The sample size of 60 for Cohort 2 was estimated based on the availability of target patient population to evaluate the therapeutic benefit for those patients.

Date of the Protocol: 18 March 2020

3 TABLE OF CONTENTS

1 ADMINISTRATIVE INFORMATION ... 4
2 STUDY SYNOPSIS ... 5
3 TABLE OF CONTENTS ... 10
3.1 List of Tables ... 14
3.2 List of Figures ... 14
3.3 List of Appendices and Supplements ... 14
4 LIST OF ABBREVIATIONS AND DEFINITION OF TERMS ... 15
5 INTRODUCTION ... 17
5.1 Investigational Medicinal Product ... 17
5.2 Background ... 17
5.2.1 Progressive Familial Intrahepatic Cholestasis ... 17
5.2.2 Summary of Clinical and Nonclinical Studies ... 19
5.3 Rationale ... 20
5.3.1 Risk/Benefit ... 21
6 STUDY OBJECTIVES ... 22
6.1 Primary Objectives ... 22
7 INVESTIGATIONAL PLAN ... 23
7.1 Overall Study Design and Plan ... 23
7.1.1 Description ... 23
7.1.2 Schedule of Assessments ... 26
7.1.3 Study Procedures and Assessments ... 34
7.1.3.1 Screening Period (Days -56 to 0) (For Cohort 2 only) ... 34
7.1.3.2 Treatment Period ... 35
7.1.3.3 End of Treatment/Early Termination/Start of Optional Extension Period ... 39
7.1.3.4 Follow-up Visit ... 40
7.1.3.5 Optional Extension Period ... 40
7.1.3.6 Optional Extension Period End of Treatment ... 40
7.2 Study Population ... 41
7.2.1 Inclusion Criteria ... 41
7.2.2 Exclusion Criteria ... 42
7.2.3 Withdrawal of Patients ... 44
7.2.4 Study Termination by Sponsor ... 45
8 TREATMENT OF PATIENTS ... 46
8.1 Identity of Study Drug ... 46

8.2 Administration of Study Drug 46
8.3 Study Drug Packaging and Labelling 47
8.3.1 Packaging and Labelling 47
8.3.2 Storage 48
8.3.3 Blinding and Randomization of Study Drug 48
8.4 Procedure for Breaking the Randomization Code 48
8.5 Patient Compliance 48
8.6 Patient Identification 48
8.7 Study Drug Accountability 49
8.8 Concomitant Therapy and Prohibited Medications 49
9 ASSESSMENT OF EFFICACY 50
9.1 Efficacy Endpoints 50
9.1.1 Primary Efficacy Endpoints 50
9.1.2 Secondary Efficacy Endpoints 50
9.1.3 Exploratory Efficacy Endpoints 51
9.2 Efficacy Assessments 52
9.2.1 Serum Bile Acids 52
9.2.2 Itching and Sleep Score 52
9.2.3 Growth 53
9.2.4 Biomarker Samples 53
9.2.5 Change of Antipruritic Medication 53
9.2.6 Quality of Life Questionnaire (PedsQL) 53
9.2.7 PELD/MELD Score 53
9.2.8 Fibroscan® 54
9.2.9 Markers of Fibrosis 54
9.2.10 Liver Biopsy 54
9.2.11 Global Impression of Change and Global Impression of Symptom Measures 54
10 ASSESSMENT OF SAFETY 55
10.1 Adverse Events 55
10.1.1 Definitions and Investigator Assessments 55
10.1.1.1 Clinical Significance 55
10.1.1.2 Serious Adverse Events 55
10.1.1.3 Severity Assessment 56
10.1.1.4 Causality Assessment 56
10.1.2 Recording of Adverse Events 57

10.1.3 Recording and Reporting of Serious Adverse Events 58
10.1.4 Reporting of Suspected Unexpected Serious Adverse Reactions 59
10.2 Laboratory Values/Vital Signs/Physical Examinations and Other Safety Assessments 60
10.2.1 Laboratory Assessments 60
10.2.2 Individual Patient Safety Monitoring 61
10.2.2.1 Liver Monitoring 61
10.2.2.2 Close Observation 62
10.2.2.3 De-challenge/Re-challenge for Liver and Clinical Hepatitis Monitoring 63
10.2.2.4 Diarrhea 63
10.2.2.5 Monitoring after Permanent Discontinuation of Study Drug Due to Safety 63
10.2.3 Demographics/Medical and Surgical History (For Cohort 2 only) 64
10.2.4 Clinical Genetic Testing (For Cohort 2 only) 64
10.2.5 Physical Examination 64
10.2.6 Vital Signs 64
10.2.7 Abdominal ultrasound 64
10.2.8 Overdose 65
10.2.9 Pregnancy 65
11 STATISTICAL EVALUATION 66
11.1 Sample Size and Power 66
11.2 Statistical Methods 66
11.2.1 Statistical Analysis Sets 66
11.2.2 Methods of Statistical Analyses 66
11.2.2.1 General Principles 66
11.2.2.2 Missing Data 67
11.2.2.3 Demographic and Baseline Characteristics 67
11.2.2.4 Subject Disposition 67
11.2.2.5 Evaluation of Primary Efficacy Variables 67
11.2.2.6 Evaluation of Secondary and Exploratory Efficacy Variables 68
11.2.2.7 Evaluation of Safety Variables 69
11.2.2.8 Compliance and Exposure 69
11.2.3 Interim Analysis 69
11.2.4 Data Safety Monitoring Board 70

12 DIRECT ACCESS TO SOURCE DATA/NOTES 71
13 QUALITY CONTROL AND QUALITY ASSURANCE 72
13.1 Conduct of the Study 72
13.2 Study Monitoring... 72
14 ETHICS 74
14.1 Independent Ethics Committee/Institutional Review Board.................. 74
14.2 Written Informed Consents and Assents 74
15 DATA HANDLING AND RECORD KEEPING... 76
15.1 Case Report Forms/Source Data Handling 76
15.2 Retention of Essential Documents... 76
16 FINANCING AND INSURANCE 77
17 PUBLICATION POLICY 78
18 REFERENCE LIST... 79
19 APPENDICES 80

3.1 List of Tables

Table 1 Schedule of Assessments (Screening Period through Follow-up Period) .. 27
Table 2 Schedule of Assessments (Optional Extension Period) 33
Table 3 Dosing and Capsule Strength 47
Table 4 Routine Laboratory Parameters 61

3.2 List of Figures

Figure 1 Study Design 26

3.3 List of Appendices and Supplements

Appendix 1 Concomitant Medication Guidelines 80
Appendix 2 Diary Questions including Albireo Patient-Reported Outcomes/Observer-Reported Outcome Instrument 82
Appendix 3 Patient Global Impression of Change (PGIC) and Patient Global Impression of Symptoms (PGIS) 85
Appendix 4 Pediatric Quality of Life Inventory (PedQL) Questionnaire 90
Appendix 5 Guideline for Fat-Soluble Vitamin Supplementation 111
Appendix 6 Contraceptive Requirements 114
Appendix 7 Blood Volumes 117

4 LIST OF ABBREVIATIONS AND DEFINITION OF TERMS

ADME	absorption, distribution, metabolism, and excretion
AE(s)	adverse event(s)
AFP	alfa-fetoprotein
ALT	alanine aminotransferase
APRI	AST to platelet ratio index
ASBT	apical sodium-dependent bile transporter (also known as IBAT)
AST	aspartate aminotransferase
ATC	Anatomical Therapeutic Chemical
BMI	body mass index
BSEP	bile salt export pump
CI	confidence interval
CPK	creatine phosphokinase
CRA	clinical research associate
CYP	cytochrome P450
DSMB	Data and Safety Monitoring Board
eCRF	electronic case report form
eDiary	electronic diary
EU	European Union
EudraCT	European Union drug regulatory agency clinical trial
FAS	full analysis set
FDA	United States Food and Drug Administration
FIC1	familial intrahepatic cholestasis-1
GCP	Good Clinical Practice
GGT	gamma-glutamyl transferase
GI	gastrointestinal
IB	Investigator's Brochure
IBAT	ileal bile acid transporter (also known as ASBT)
ICF	informed consent form
ICH	International Council for Harmonisation
IEC	Independent Ethics Committee
INR	international normalized ratio
IRB	Institutional Review Board
IWRS	Interactive Web Response System
LDH	lactate dehydrogenase
LFT	liver function test

LPLV	last patient last visit
MDR3	multidrug-resistance protein 3
MedDRA	Medical Dictionary for Regulatory Activities
MELD	model for end-stage liver disease
NAPPED	NAtural Course and Prognosis of PFIC and Effect of Biliary Diversion
ObsRO	observer-reported outcome
PBC	primary biliary cholangitis
p-C4	plasma 7α-hydroxy-4-cholesten-3-one
PCS	potentially clinically significant
PedsQL	Pediatric Quality of Life Inventory
PELD	pediatric end-stage liver disease
PFIC	progressive familial intrahepatic cholestasis
PGIC	patient global impression of change
PGIS	patient global impression of symptoms
PRO	patient-reported outcome
PT	prothrombin time
PVM	pharmacovigilance manager
QoL	quality of life
SAE(s)	serious adverse event(s)
SAP	statistical analysis plan
s-BA	serum bile acid
s-FGF19	serum fibroblast growth factor 19
SD	standard deviation
SOC	system organ class
SUSAR	suspected unexpected serious adverse reaction
TEAE(s)	treatment-emergent adverse event(s)
ULN	upper limit of normal
US	United States
WHO	World Health Organization

5 INTRODUCTION

5.1 Investigational Medicinal Product

A4250 is a small molecule and a potent selective inhibitor of the ileal bile acid transporter (IBAT), also known as the apical sodium-dependent bile acid transporter (ASBT). IBAT is an integral brush border membrane glycoprotein that co-transports sodium and bile acids and appears to be a critical component in the regulation of the bile acid pool size in animals and man. This transporter, expressed in the distal ileum, is a key element in the enterohepatic circulation of bile acids since it facilitates the high-affinity, high-capacity reabsorption of bile acids. Indeed, 95% of secreted bile acids are reabsorbed via IBAT [Hofmann 2009; Miethke 2016]. A4250 is orally administered and acts locally in the gut where it binds reversibly to IBAT to decrease the reuptake of bile acids. A4250 has minimal systemic exposure at the predicted therapeutic dose ranges.

No safety signals were found with A4250 in preclinical safety and toxicity studies. In the first clinical study of A4250 in healthy volunteers, A4250 was given as a single oral dose up to 10 mg or daily oral doses up to 3 mg/day for 7 days. A4250 was well tolerated and the only findings were gastrointestinal (GI) related effects, such as diarrhea, at higher doses.

In an exploratory Phase 2 study in children with cholestatic pruritus, A4250 was given as oral doses of 10 to 200 µg/kg/day for 4 weeks. A4250 reduced mean levels of serum bile acids (s-BAs) in all cohorts. In 11 of 13 progressive familial intrahepatic cholestasis (PFIC) patients, substantial reductions (ranging from 43% to 98%) were observed. Patient-reported diary data documented improvement of pruritus during treatment. A4250 was well tolerated. No serious adverse events (SAEs) were deemed related to study drug, and most adverse events (AEs), including some increased transaminases, were considered unrelated to study drug and transient in nature.

5.2 Background

5.2.1 Progressive Familial Intrahepatic Cholestasis

PFIC is a rare autosomal recessive cholestatic liver disease estimated to affect between one in every 50,000 to 100,000 children born worldwide. PFIC represents 10% to 15% of causes of cholestasis in children and 10% to 15% of liver transplantation indications in children. All types of PFIC exist worldwide and both sexes appear to be equally affected.

The common underlying pathogenesis of PFIC is disruption of bile formation and bile transport through the liver [Jacquemin 2000]. The classification of PFIC has evolved over the years. The most commonly used subclassification is PFIC Types 1, 2, and 3 which is based on the associated affected gene and described in more detail below.

- PFIC, Type 1: also referred to as “Byler disease” or “familial intrahepatic cholestasis 1 (FIC1) protein deficiency.” FIC1 protein is located on the canalicular membrane of hepatocytes and facilitates movement of aminophospholipids from the outer to inner leaflet

of the plasma membrane of the hepatocyte. The *ATP8B1* gene encodes FIC1 protein. Biallelic pathologic variants in the *ATP8B1* gene are associated with FIC1 dysfunction and classified clinically as PFIC Type 1 disease.

- PFIC, Type 2: also referred to as "Byler syndrome" or "bile salt export pump (BSEP) deficiency." BSEP is a transporter protein that is expressed at the canalicular membrane of hepatocytes and is the primary exporter of bile acids. The *ABCB11* gene encodes the BSEP protein. Biallelic pathologic variations in the *ABCB11* gene is associated with BSEP dysfunction and is classified clinically as PFIC Type 2 disease.
- PFIC, Type 3: is caused by a deficiency of the multidrug-resistance protein 3 (MDR3) due to mutations in the *ABCB4* gene. MDR3 is a phospholipid translocase critical for phospholipid secretion.

Severe pruritus is common in children diagnosed with PFIC. Itching (and subsequent scratching) is a significant morbidity for these patients and their families [Suchy 2007]. A more severe degree of pruritus is experienced compared to patients with other forms of liver disease and to other pruritic conditions such as atopic dermatitis [Murray 2011]. In patients with PFIC, liver biopsy reveals canalicular cholestasis and, later, the appearance of portal fibrosis. Serum biochemistry indicates cholestasis with hyperbilirubinemia, elevated alanine aminotransferase (ALT) and aspartate aminotransferase (AST). The concentrations of bile acids in serum are very high, while serum gamma-glutamyl transferase (GGT) activity (the exception being MDR3 variants) and cholesterol [Hori 2010] are normal. Symptoms of portal hypertension and liver failure will develop during the course of the disease [Davit-Spraul 2009; Alissa 2008]. Symptoms develop early; median age at onset of symptoms is 2 months, and 78% of PFIC patients present with jaundice [Pawlikowska 2010]. The life-threatening and debilitating nature of PFIC is reflected by the fact that survival in those not resorting to surgery is 50% at 10 years of age and almost zero at 20 years of age. Approximately half of PFIC patients undergo liver transplantation [Davit-Spraul 2009] and treatment resistant pruritus is the leading indication for the surgical procedure partial external biliary diversion, mostly in PFIC Type 1 and 2 patients.

PFIC is life-threatening and debilitating. Currently, the treatment for PFIC is palliative and there is currently no pharmaceutical treatment approved for use in PFIC. The therapeutic choices are restricted to non-specific therapy of the symptoms and signs of the disease such as nutritional support, preventing vitamin deficiencies, and treatment of extrahepatic features. Medical treatment options include off-label use of ursodeoxycholic acid, rifampin, antihistamines, and naltrexone. A minority of patients respond nominally and transiently to these interventions. Biliary diversion is used to decrease systemic bile acids through interruption of the enterohepatic circulation and so avoid transplantation. Liver transplantation is typically only viewed as an option when patients have failed medical treatment and/or biliary diversion and have liver failure or persistent uncontrolled pruritus.

5.2.2 Summary of Clinical and Nonclinical Studies

This is a summary of nonclinical and clinical studies. More detail is provided in the Investigator Brochure ([IB] see A4250 IB).

A4250 is a potent selective IBAT inhibitor with no safety signals seen in the safety pharmacological studies. In addition, no safety signals were seen in repeat-dose oral toxicity studies in rats with up to 26 weeks of daily dosing and in dogs with up to 39 weeks of daily dosing. No safety signals were identified when A4250 was administered daily to juvenile rats from age 14 days to 63 days.

A4250 is minimally absorbed with very low systemic exposure in both humans and other species. In blood, protein binding is high (>99.7%). Systemically absorbed A4250 is slowly metabolized in humans and in preclinical studies by hydroxylation only, a metabolite that retains IBAT activity. A4250 did not show any significant inhibition of cytochrome P450 (CYP) enzymes in the rat. A4250 inhibited human CYP3A4 and CYP2D6 (IC_{50}: 16 µmol/L for both enzymes), and CYP2C9 (IC_{50}: 1.2 µmol/L).

To date, A4250 has been studied in 3 Albireo-sponsored clinical studies: a study in healthy volunteers evaluating single and multiple administration of A4250; a single-dose absorption, distribution, metabolism, and excretion (ADME) study; and a Phase 2 study in children with cholestatic pruritus. In addition, an investigator-sponsored study has been conducted in patients with primary biliary cholangitis (PBC). In total, 98 subjects/patients have been exposed to A4250.

In the first clinical study (A4250-001), A4250 was given to healthy volunteers as a single oral dose up to 10 mg or daily oral doses up to 3 mg/day for 7 days. Following a single oral dose of 10 mg, the median maximum drug concentration was 0.2 nmol/L and the area under the curve was 0.8 nmol × h/L (n=6). Pharmacodynamic evaluation of bile acid synthesis biomarkers (serum fibroblast growth factor 19 [s-FGF19] and plasma 7α Hydroxy 4-cholesten-3-one [p-C4]) identified the expected decrease in s-FGF19 and an increase in p-C4. In addition, total plasma bile acids decreased and total fecal bile acids increased in A4250-treated patients compared to placebo. A4250 was generally well tolerated. The only findings were GI-related effects, such as diarrhea at higher doses.

In the ADME study (A4250-007), healthy subjects were administered a single oral dose of 3 mg [^{14}C]-A4250 capsule. The main excretion route was determined to be fecal. There were no quantifiable concentrations of A4250 in plasma or total radioactivity in plasma or whole blood. A4250 was well tolerated. AEs were reported and most were mild and not related to A4250. The 3 AEs experienced by 2 subjects reported as related to study drug were GI in nature, including diarrhea (2 AEs; 2 subjects) and abdominal pain (1 AE; 1 subject).

In an exploratory Phase 2 study in children with cholestatic pruritus (A4250-003), A4250 was administered as oral daily doses of 10 to 200 µg/kg/day for 4 weeks. Twenty patients (8 females, 12 males) aged 1 to 17 years were enrolled in this open-label

dose-escalation study. Four patients were re-enrolled into a second dose level. No treatment-related SAEs were reported, and most AEs, including some increased transaminases, were transient. Four-week, once-daily treatment with A4250 reduced mean levels of s-BA in all cohorts. Substantial s-BA reductions in 11 of 13 PFIC patients (ranging from 43% to 98%) were observed. A4250 also reduced s-BA in the 11 non-PFIC patients, but to a lesser extent than in PFIC patients. Patient-reported diaries documented improvement of the visual analogue scale itch score in 17 of 24 patients during treatment. The dose with the greatest improvement showed a mean decrease (± standard error of the mean) of 2.9 ±1.1 from baseline. The results from this trial form the basis of the current study design.

5.3 Rationale

In this investigational study, A4250 is under development for treatment of PFIC and is also being considered for other orphan cholestatic liver diseases.

There is currently no medical treatment approved for PFIC. In PFIC patients with severe pruritus, biliary diversion surgery is used to decrease systemic bile acids through interruption of the enterohepatic circulation and thereby reduce pruritus [Whitington 1988]. Liver transplantation is typically used as an option when patients have failed medical treatment and/or biliary diversion and have liver failure or persistent uncontrolled pruritus.

By inhibiting IBAT with high selectivity and potency, A4250 has the potential to relieve cholestasis and improve liver function without surgical intervention in patients with PFIC. Since A4250 is minimally absorbed and achieves a substantial reduction of s-BA, it has the potential to be a safer and less invasive alternative to surgical biliary drainage. The rationale for using A4250 is to decrease s-BA levels, and to reduce the major morbidity of pruritus, improving the health and wellbeing of patients affected with PFIC. Ultimately, this noninvasive therapeutic approach has the potential to not only improve quality of life (QoL), but also reduce the need for biliary diversion and subsequent liver failure and liver transplantation.

The doses selected for the A4250-005 Phase 3 study, 40 and 120 µg/kg/day, were based on the efficacy and safety data generated from patients with PFIC (n=10 + 3 re-entered) and supporting data from all patients (n=20 + 4 re-entered) in the five dose groups dosed in the A4250-003 Phase 2 study. Improvements of s-BAs and pruritus were observed with doses from 30 to 100 µg/kg/day with a dose-related trend. The best dose response in all patients was at the 60 µg/kg/day for s-BAs and 100 µg/kg/day for pruritus. The best dose response in the PFIC subgroup was at 30 µg/kg/day for both s-BAs and pruritus. Patients with PFIC responded well to the doses given from 30 to 200 µg/kg/day; however, a clear dose relationship could not be established in order to select a single dose for the A4250-005 study. As efficacy was observed at both low and high doses, 40 and 120 µg/kg/day were considered to be the most optimal for the A4250-005 study. The higher dose, 120 µg/kg/day, will be used in this study, with the possibility to down-titrate to the lower dose after a minimum of 1 week on the high

dose. The patient should return to the higher dose as soon as deemed appropriate by the investigator. More than one upward dose titration (from 40 µg/kg/day directly to 120 µg/kg/day) for the same event is not recommended. Any dose titration should be done in consultation with the sponsor Medical Monitor or designee.

Rationale for patient selection:

Cohort 1: Patients in Cohort 1 are children with PFIC Types 1 and 2 who have completed study A4250-005 (PEDFIC 1) and will be enrolled to collect data on the efficacy (impact on pruritus and clinical laboratory parameters) and safety of long-term administration of A4250.

Cohort 2: Cohort 2 will enroll patients with PFIC who (1) were not eligible to enroll in A4250-005 (PEDFIC 1) or (2) are eligible for enrolment in A4250-005 after recruitment of study A4250-005 has been completed. As these patients will have cholestatic liver disease with elevated s-BAs and pruritus, reduction of s-BAs with A4250 treatment is believed to provide a therapeutic benefit for these patients. These patients will be enrolled to collect data on the efficacy (impact on pruritus and clinical laboratory parameters) and safety of long-term administration of A4250.

This study will be conducted in compliance with the protocol and with the International Council for Harmonisation (ICH) guidelines on Good Clinical Practice (GCP).

5.3.1 Risk/Benefit

A4250 has been evaluated in three Albireo-sponsored clinical studies: a double-blind placebo-controlled study in healthy volunteers, a single-dose ADME study, and a Phase 2 study in children with cholestatic pruritus. In addition, an investigator-sponsored study has been conducted in patients with PBC.

A total of 98 subjects/patients have been exposed to A4250. Healthy subjects have been exposed to up to 10 mg as a single dose and up to 3 mg daily as part of a multiple-dose evaluation. Children with cholestatic liver disease have been treated with up to 0.2 mg/kg/day (200 µg/kg/day) for 4 weeks. In total, 2 SAEs have been reported; both were judged by the physician to be not related to the study drug.

Patients with cholestatic liver diseases suffer from excess bile acids in the liver resulting in tissue damage. A commonly used treatment in these patients is bile diversion surgery, whereby approximately 50% to 100% of the enterohepatic circulation of bile acids is interrupted. Inhibition of IBAT with A4250, thereby interrupting the enterohepatic circulation of bile acids, is therefore a potential medical alternative to surgery which could be of benefit to these patients if shown to be effective and safe. Data from the Phase 2 study showed efficacy of A4250 in reducing s-BA concentrations and pruritus in such patients.

Based on the mode of action of an IBAT inhibitor, loose stools or diarrhea could be expected. However, in the pediatric Phase 2 study with 4 weeks daily treatment, only one patient had mild transient diarrhea after a single dose that did not recur on multiple dosing.

6 STUDY OBJECTIVES

6.1 Primary Objectives

Primary Objective (Cohort 1)

To demonstrate a sustained effect of A4250 on s-BAs and pruritus in children with PFIC Types 1 and 2

Primary Objective (Cohort 2)

To evaluate the effect of A4250 on s-BAs and pruritus in patients with PFIC who either (1) do not meet eligibility criteria for Study A4250-005 (PEDFIC 1) or (2) who do meet the eligibility criteria for Study A4250-005 after recruitment of Study A4250-005 has been completed.

Secondary Objectives (Cohort 1 and 2)

The secondary objectives of this study are:

- To evaluate the long-term safety and tolerability of repeated daily doses of A4250
- To evaluate the effect of A4250 on growth
- To evaluate the effect of A4250 on biliary diversion and/or liver transplantation
- To evaluate the effect of A4250 on biochemical markers of cholestasis and liver disease

7 INVESTIGATIONAL PLAN

7.1 Overall Study Design and Plan

7.1.1 Description

This is a Phase 3, multi-center, open-label extension study to investigate the long-term efficacy and safety of a 120 µg/kg daily dose of A4250 in patients with PFIC. Cohort 1 will consist of children with PFIC Types 1 and 2 who have participated in Study A4250-005. Cohort 2 will consist of approximately 60 patients with any type of PFIC who have elevated s-BAs and cholestatic pruritus and who either (1) do not meet eligibility criteria for Study A4250-005 (PEDFIC 1) or (2) are eligible for enrolment in A4250-005 after recruitment of study A4250-005 has been completed. Up to 40 patients post biliary diversion surgery are allowed to participate in Cohort 2.

The study includes a 72-week Treatment Period and a 4-week Follow-up Period. Patients who wish to continue receiving A4250 after 72 weeks will have the option to remain on treatment in an extension period with visits every 16 weeks until the drug is commercially available, provided continued use is supported by the risk-benefit profile and the subject has not been previously withdawn or discontinued from the study. In that case the 4-week Follow-up Period will not occur.

The study data will be reviewed periodically (approximately on a quarterly basis) by a Data Safety and Monitoring Board (DSMB) until the last patient reaches 72 weeks.

Eligible patients will be enrolled into this open-label extension study and treated with a daily dose of 120 µg/kg/day of A4250 for 72 weeks. Patients not tolerating the 120 µg/kg/day dose after a minimum of 1 week for reasons other than new liver findings and severe diarrhea, as described in Section 10.2.2, will have the option to down-titrate to a lower dose (40 µg/kg/day). The patient should return to the higher dose as soon as deemed appropriate by the investigator. More than one upward dose titration (from 40 µg/kg/day directly to 120 µg/kg/day) for the same event is not recommended. Any dose titration should be done in consultation with the sponsor Medical Monitor or designee. Patients participating in the optional extension period will continue on a daily dose of 120 µg/kg/day of A4250 with the option to down-titrate to a lower dose (40 µg/kg/day).

Patients in Cohort 2 will go through a screening period consisting of:

- Visit S-1: Screening Visit 1 (Days -56 to -35).
- Visit S-2: Screening Visit 2 (Days -28 to -7)

Patients not meeting eligibility criteria may be re-screened after consultation with the Medical Monitor. Patients not fulfilling inclusion/exclusion criteria after 3 attempts are not allowed to rescreen.

All patients will have a minimum of 12 visits at the study site and 6 scheduled telephone contacts (see Figure 1) as follows:

- Visit 1: Screening/Inclusion Visit (Day 1; coincides with Visit 9 in Study A4250-005 for patients in Cohort 1)
- Visit 2: Week 4
  - Telephone contact 1: Week 8
- Visit 3: Week 12
  - Telephone contact 2: Week 18
- Visit 4: Week 22
- Visit 5: Week 24
  - Telephone contact 3: Week 30
- Visit 6: Week 36
  - Telephone contact 4: Week 42
- Visit 7: Week 46
- Visit 8: Week 48
  - Telephone contact 5: Week 54
- Visit 9: Week 60
  - Telephone contact 6: Week 66
- Visit 10: Week 70
- Visit 11: Week 72/Optional extension period
- Visit 12: Follow-up Visit; Week 76 (for those not participating in optional extension period)
- Optional extension period visits every 16 weeks; Week 88 and onwards

Additional clinic visits may be required for patients who need direct site assistance including, but not limited to, AE monitoring for safety maintenance.

Informed consent must be obtained prior to performing any study procedures. After signing the informed consent form (ICF), patients will be evaluated for study eligibility and considered enrolled in the study. For Cohort 1, patients who completed 24 weeks, or who withdrew after a minimum of 12 weeks of treatment with A4250 due to intolerable symptoms in Study A4250-005 and meeting all inclusion criteria and no exclusion criteria are eligible for this study. Patients who withdraw from A4250-005 due to a study drug related AE will not be eligible. For Cohort 2, patients meeting all inclusion criteria and no exclusion criteria are eligible for this study.

Patients/caregivers will be instructed to fill in the electronic diary (eDiary) every morning and evening for the first 24 weeks (Visit 1 through Visit 5) and the 21 days before each clinic visit thereafter (Visit 6 through Visit 12). In addition, Cohort 2 patients/caregivers will be instructed to fill in the eDiary during the screening period. Patients participating in the optional extension period will stop eDiary entries at Visit 11. The eDiary will include patient-reported outcome (PRO) and observer-reported outcome (ObsRO) items for evaluation of itching (PRO), scratching (ObsRO), and sleep disturbance (PRO and ObsRO). Observer-reported outcomes in patients of all ages will be recorded by a caregiver. If possible, the same caregiver will complete the ObsRO items throughout the study. Additionally, caregivers will be requested to report in the diary the time that study drug was administered during the Treatment Period.

The patient will return to the clinic after 4 weeks and 12 weeks and thereafter every 10 to 12 weeks for follow-up measurements. Between the clinic visits there will be a telephone contact with the patient/caregiver to report AEs.

At Visit 11, patients will be offered the choice to continue receiving A4250 treatment in an optional extension period. In the extension period the patient will receive 120 µg/kg/day A4250, returning to clinic every 16 weeks until the drug is commercially available, provided continued use is supported by the risk-benefit profile and the subject has not been previously withdawn or discontinued from study.

If the patient is prematurely withdrawn from the study, all assessments scheduled for Visit 11 will be performed at the time the patient withdraws. If a patient participating in the optional extension study withdraws prior to commercial drug availability, all assessments scheduled for the optional Extension Period End of Treatment visit will be performed.

If a liver biopsy is performed according to the local regulations or standard of care at any time during A4250-008, the biopsy results should be recorded in the electronic case report form (eCRF). If the patient had liver biopsy results collected in study A4250-005 or prior to Week 24 in study A4250-008, the patient will be asked to consent to a follow-up liver biopsy, if allowed per local regulations, at Visit 11, unless 2 prior liver biopsy results have been collected with at least 1 year between biopsies. Liver biopsies will not be performed in Spain.

For those not participating in the optional extension period, Visit 12 will take place 28 days after Visit 11. All patients prematurely withdrawn will have this visit 28 days after the last dose of study drug for AE and other follow-up.

If a patient discontinues prior to Week 72, additional telephone contacts will be made every 3 months after the Follow-up Visit (Visit 12) up to Week 72 to gather whether or not the patient has had biliary diversion or liver transplantation.

Details of study assessments are provided in the schedule of assessments (Table 1 and Table 2).

Figure 1 Study Design

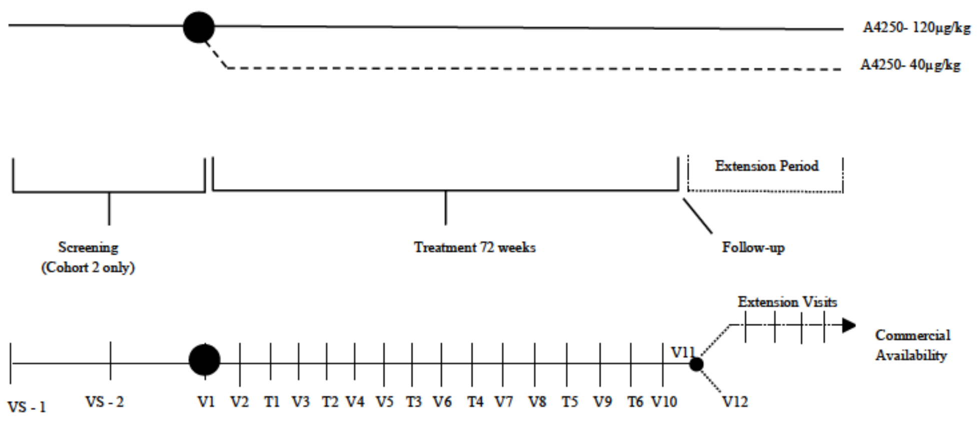

T: telephone contact; V: study visit; VS: Visit Screening; W: study week

Note: Visit 1 coincides with Visit 9 of Study A4250-005. Patients who are not tolerating the 120 µg/kg/day dose may be down-titrated to the 40 µg/kg/day dose following a minimum of 1 week of treatment. The patient can return to the higher dose as soon as deemed appropriate.

End of Study

The end of the study is defined as follows:

a) End of study in one country: last patient last visit (LPLV) and sites are closed.

b) End of study globally: LPLV globally and all sites closed

7.1.2 Schedule of Assessments

The schedule of assessments for the screening period through the follow-up period is presented in Table 1 and the schedule of assessments for the optional extension period is presented in Table 2. For information on blood volumes, please see Appendix 7.

Table 1 Schedule of Assessments (Screening Period through Follow-up Period)

	Screening Period (Cohort 2 only)		Treatment Period											Follow-up Period[t]
**Study Activity**			**Screening/ Inclusion Visit**	**4 weeks**	**12 weeks**	**22 weeks**	**24 weeks**	**36 weeks**	**46 weeks**	**48 weeks**	**60 weeks**	**70 weeks**	**72 weeks**	**76 weeks**
**Study Days (±window)**	**-56 – (-35) ±2**	**-28 – (-7) ±2**	**1**	**28 (±5)**	**84 (±7)**	**154 (±7)**	**168 (±7)**	**252 (±7)**	**322 (±7)**	**336 (±7)**	**420 (±7)**	**490 (±7)**	**504 (±7)**	**532 (±7)**
**Clinic Visits**	**Visit S-1**	**Visit S-2**	**Visit 1[a]**	**Visit 2**	**Visit 3**	**Visit 4**	**Visit 5**	**Visit 6**	**Visit 7**	**Visit 8**	**Visit 9**	**Visit 10**	**Visit 11/EOT[b]/Start of Optional Extension Period[r]**	**Visit 12[c]**
Informed consent	X		X[d]											
Inclusion/exclusion criteria	X		X											
Demography/Medical and Surgical History	X													
Concomitant medication[e]	X	X	X	X	X	X	X	X	X	X	X	X	X	X
Physical examination[f]	X[f]		X[f]		X[f]		X[f]			X			X	
Skin examination	X		X	X	X		X			X			X	
Vital signs[g]	X	X	X	X	X	X	X	X	X	X	X	X	X	X

	**Screening Period (Cohort 2 only)**		**Treatment Period**											**Follow-up Period[t]**
**Study Activity**			**Screening/ Inclusion Visit**	**4 weeks**	**12 weeks**	**22 weeks**	**24 weeks**	**36 weeks**	**46 weeks**	**48 weeks**	**60 weeks**	**70 weeks**	**72 weeks**	**76 weeks**
**Study Days (±window)**	**-56 – (-35) ±2**	**-28 – (-7) ±2**	**1**	**28 (±5)**	**84 (±7)**	**154 (±7)**	**168 (±7)**	**252 (±7)**	**322 (±7)**	**336 (±7)**	**420 (±7)**	**490 (±7)**	**504 (±7)**	**532 (±7)**
**Clinic Visits**	**Visit S-1**	**Visit S-2**	**Visit 1[a]**	**Visit 2**	**Visit 3**	**Visit 4**	**Visit 5**	**Visit 6**	**Visit 7**	**Visit 8**	**Visit 9**	**Visit 10**	**Visit 11/EOT[b]/Start of Optional Extension Period[r]**	**Visit 12[c]**
eDiary: itching, scratching, and sleep scores[h]	Daily diary entry							For 21 days before each clinic visit for Visit 6 through Visit 12						
Clinical chemistry[i]	X		X	X	X		X	X		X	X		X	X
Hematology[i]			X	X	X		X	X		X	X		X	X
Urinalysis[i]			X				X			X			X	
International normalized ratio		X		X	X	X		X	X		X	X		X
Serum bile acids[j]	X	X	X	X	X	X	X	X	X	X	X	X	X	X
Autotaxin			X	X			X			X			X	
p-C4			X	X			X			X			X	
Alfa-fetoprotein			X				X			X			X	
Vitamins A[j], E and 25-hydroxy vitamin D			X		X	X		X	X		X	X		

	**Screening Period (Cohort 2 only)**		**Treatment Period**											**Follow-up Period[t]**
**Study Activity**			**Screening/ Inclusion Visit**	**4 weeks**	**12 weeks**	**22 weeks**	**24 weeks**	**36 weeks**	**46 weeks**	**48 weeks**	**60 weeks**	**70 weeks**	**72 weeks**	**76 weeks**
**Study Days (±window)**	**-56 – (-35) ±2**	**-28 – (-7) ±2**	**1**	**28 (±5)**	**84 (±7)**	**154 (±7)**	**168 (±7)**	**252 (±7)**	**322 (±7)**	**336 (±7)**	**420 (±7)**	**490 (±7)**	**504 (±7)**	**532 (±7)**
**Clinic Visits**	**Visit S-1**	**Visit S-2**	**Visit 1[a]**	**Visit 2**	**Visit 3**	**Visit 4**	**Visit 5**	**Visit 6**	**Visit 7**	**Visit 8**	**Visit 9**	**Visit 10**	**Visit 11/EOT[b]/Start of Optional Extension Period[r]**	**Visit 12[c]**
Abdominal ultrasound			X				X			X			X	
Fibroscan (where available)			X				X			X			X	
QoL questionnaire (PedsQL)			X				X			X			X	
Patient/Caregiver/Clinician Patient Global Impression of Change			X[q]	X	X		X			X			X	
Patient/Caregiver/Clinician Patient Global Impression of Symptoms			X	X	X		X			X			X	
Pregnancy test[k]	X[k]	X	X[k]	X	X	X	X	X	X	X	X	X	X	X
Telephone contact			Patients will be contacted via telephone for adverse event monitoring at Weeks 8, 18, 30, 42, 54, and 66											
Liver biopsy[l]													X	

	**Screening Period (Cohort 2 only)**		**Treatment Period**											**Follow-up Period[t]**
**Study Activity**			**Screening/ Inclusion Visit**	**4 weeks**	**12 weeks**	**22 weeks**	**24 weeks**	**36 weeks**	**46 weeks**	**48 weeks**	**60 weeks**	**70 weeks**	**72 weeks**	**76 weeks**
**Study Days (±window)**	**-56 – (-35) ±2**	**-28 – (-7) ±2**	**1**	**28 (±5)**	**84 (±7)**	**154 (±7)**	**168 (±7)**	**252 (±7)**	**322 (±7)**	**336 (±7)**	**420 (±7)**	**490 (±7)**	**504 (±7)**	**532 (±7)**
**Clinic Visits**	**Visit S-1**	**Visit S-2**	**Visit 1[a]**	**Visit 2**	**Visit 3**	**Visit 4**	**Visit 5**	**Visit 6**	**Visit 7**	**Visit 8**	**Visit 9**	**Visit 10**	**Visit 11/EOT[b]/Start of Optional Extension Period[r]**	**Visit 12[c]**
Study drug dispensed[m]			X	X	X		X	X		X	X		X[s]	
Adverse events[no]	Continuous collection													
Study drug compliance evaluated				X	X[o]		X	X[p]		X	X		X[p]	

eCRF: electronic case report form; eDiary: electronic diary; EOT: end of treatment; p-C4: plasma 7α Hydroxy 4-cholesten-3-one; PedsQL: pediatric quality of life; QoL: quality of life.

a For patients in Cohort 1, Visit 1 coincides with Visit 9 in Study A4250-005. Assessments that have been performed at Visit 9 during Study A4250-005 will not be repeated.

b Assessments must also be performed at the time a patient is prematurely withdrawn from the study. If a patient discontinues prior to week 72, additional phone contact will be made every 3 months up to a total study participation of 72 weeks to assess if the patient has had biliary diversion or liver transplantation.

c Assessments must be performed 28 days following the final dose of study drug.

d Only for Cohort 1.

e Includes current medications.

f A complete physical exam will be performed at Visits S-1, 1, 3, 5, 8, and 11/EOT.

g Includes blood pressure, pulse, respiratory rate, temperature, height (or length depending on age) and body weight (using a certified weight scale). Body mass index will be calculated.

	Screening Period (Cohort 2 only)		Treatment Period											Follow-up Period[t]
**Study Activity**			**Screening/ Inclusion Visit**	**4 weeks**	**12 weeks**	**22 weeks**	**24 weeks**	**36 weeks**	**46 weeks**	**48 weeks**	**60 weeks**	**70 weeks**	**72 weeks**	**76 weeks**
**Study Days (±window)**	**-56 – (-35) ±2**	**-28 – (-7) ±2**	**1**	**28 (±5)**	**84 (±7)**	**154 (±7)**	**168 (±7)**	**252 (±7)**	**322 (±7)**	**336 (±7)**	**420 (±7)**	**490 (±7)**	**504 (±7)**	**532 (±7)**
**Clinic Visits**	**Visit S-1**	**Visit S-2**	**Visit 1[a]**	**Visit 2**	**Visit 3**	**Visit 4**	**Visit 5**	**Visit 6**	**Visit 7**	**Visit 8**	**Visit 9**	**Visit 10**	**Visit 11/EOT[b]/Start of Optional Extension Period[r]**	**Visit 12[c]**

h Itching, scratching, and sleep will be assessed via an eDiary (issued with any necessary training to patients/caregivers at Visit 1). Patients/caregivers will be instructed to fill in the eDiary every morning and evening for the first 24 weeks and the last 21 days before all the remaining visits to the clinic. There will be no interruption between Visits 11 and 12. Patients participating in the extension period will stop eDiary entries at Visit 11.

i See Table 4 for detailed parameters.

j Patients will fast (only water intake is permissible) for at least 4 hours prior to the collection of samples for serum bile acids and vitamin A. Exceptions can be made for infants, less than 12 months of age, if unable to fast for the entire 4 hours.

k For girls who have reached menarche. Serum test will be performed at Visit S-1 for Cohort 2 only and Visit 1 (for Cohort 1 only); urine test will be performed at all other visits. If a urine pregnancy test is positive, a serum pregnancy test should be performed to confirm the pregnancy.

l If a liver biopsy is performed at any time during A4250-008 according to the local regulations or standard of care, the biopsy results should be recorded in the eCRF. If the patient has liver biopsy results collected in study A4250-005 or prior to Week 24 in study A4250-008, the patient will be asked to consent to a follow up liver biopsy, if allowed per local regulations, at Visit 11 unless 2 prior liver biopsy results have been collected with at least 1 year between biopsies. Liver biopsies will not be performed in Spain.

m Study drug will be taken once daily from Day 1 through Day 504 as described in Section 8.2.

n Adverse event information will be collected from the time of signing of the informed consent form to study discontinuation.

o For hepatic adverse events and/or hepatic decompensation, a PK sample should be collected as close to the onset of the event as possible

p Dosage form acceptability questions will be asked of the caregiver and/or patient at Visits 3, 6, and 11.

q Not applicable for Cohort 2.

	Screening Period (Cohort 2 only)		Treatment Period											Follow-up Period[t]
**Study Activity**			**Screening/ Inclusion Visit**	**4 weeks**	**12 weeks**	**22 weeks**	**24 weeks**	**36 weeks**	**46 weeks**	**48 weeks**	**60 weeks**	**70 weeks**	**72 weeks**	**76 weeks**
**Study Days (±window)**	**-56 – (-35) ±2**	**-28 – (-7) ±2**	**1**	**28 (±5)**	**84 (±7)**	**154 (±7)**	**168 (±7)**	**252 (±7)**	**322 (±7)**	**336 (±7)**	**420 (±7)**	**490 (±7)**	**504 (±7)**	**532 (±7)**
**Clinic Visits**	**Visit S-1**	**Visit S-2**	**Visit 1[a]**	**Visit 2**	**Visit 3**	**Visit 4**	**Visit 5**	**Visit 6**	**Visit 7**	**Visit 8**	**Visit 9**	**Visit 10**	**Visit 11/EOT[b]/Start of Optional Extension Period[r]**	**Visit 12[c]**

r Patients who wish to continue receiving A4250 after 72 weeks, will have the option to remain on treatment in an extension period until the drug is commercially available, provided continued use is supported by the risk-benefit profile and the subject has not been previously withdawn or discontinued from study.

s Dispensation only for patients moving into the optional extension period.

t Only for patients who stop treatment at 72 weeks.

Table 2 Schedule of Assessments (Optional Extension Period)

	Extension Period	Extension Period EOT
**Study Activity**	**Every 16 weeks**	
**Study Days (±window)**	**Every 112 days (±7)**	
**Concomitant medication**[a]	X	X
**Vital signs**[b]	X	X
**Clinical chemistry**[c]	X	X
**s-BA**[d]	X	X
**Vitamins A**[d]**, E, and 25-hydroxy vitamin D**	X	X
**International normalized ratio**	X	X
**Urine pregnancy**[e]	X	X
**Adverse events**[f]	X	X
**Study drug compliance evaluated**	X	X
**Study drug dispensed**[g]	X	
**QoL questionnaire (PedsQL)**	X	X
**Patient/Caregiver/Clinician Patient Global Impression of Change**	X	X
**Patient/Caregiver/Clinician Patient Global Impression of Symptoms**	X	X

a Includes current medications.
b Includes blood pressure, pulse, respiratory rate, temperature, height (or length depending on age) and body weight (using a certified weight scale). Body mass index will be calculated.
c See Table 4 for detailed parameters.
d Patients will fast (only water intake is permissible) for at least 4 hours prior to the collection of samples for serum bile acids and vitamin A.
e For girls who have reached menarche.
f For hepatic adverse events and/or liver decompensation, a PK sample should be collected as close to the onset of the event as possible
g Study drug will be taken once daily throughout the extension period.

7.1.3 Study Procedures and Assessments

7.1.3.1 Screening Period (Days -56 to 0) (For Cohort 2 only)

Day -56 to Day -35/Clinic Visit S-1

Cohort 2 patients will undergo a Screening Visit up to 56 days prior to the planned first day of study treatment. Screening procedures and assessments are as follows:

- Obtain written informed consent
- Assess inclusion/exclusion criteria (Section 7.2)
- Record demographics
- Document concomitant medications
- Medical and surgical history (date of diagnosis of PFIC Type, prior investigational medications for PFIC, historical liver function test [LFT] values, any surgery performed, any other diagnosis, and historical liver biopsy data)
- Collect, if available, past confirmatory clinical genetic laboratory report for PFIC type and send to central reader for review. If a historical report is equivocal, unavailable, or unobtainable, a blood sample for clinical genetic testing will be collected to determine eligibility (Section 10.2.4)
- Physical examination (Section 10.2.5) and vital signs (Section 10.2.6)
- Skin examination (Section 10.2.5)
- eDiary training and compliance requirements; caregivers and/or patients begin daily recording of pruritus using the eDiary (Appendix 2)
- Clinical chemistry (Section 10.2.1)
- s-BA (Section 9.2.1)
- Serum pregnancy test for girls who have reached menarche. Please see Appendix 6 for contraceptive requirements
- AE monitoring

Day -28 to Day -7/Clinic Visit S-2

A second Screening Visit will be performed for patients in Cohort 2. Screening procedures and assessments are as follows:

- Review concomitant medications
- Vital signs (Section 10.2.6)
- Albireo PRO/ObsRO eDiary review for compliance
- s-BA (Section 9.2.1)

- International normalized ratio (INR)
- Urine pregnancy test for girls of who have reached menarche
- AE monitoring
- Clinical chemistry retest (if applicable)

7.1.3.2 Treatment Period

Study Day 1/Visit 1

For Cohort 1, patients will undergo a Screening/Inclusion Visit that coincides with Visit 9 of Study A4250-005.

For Cohort 2: Albireo PRO/ObsRO eDiary review for compliance and entrance criteria requirement (see Inclusion Criteria 2 [Cohort 2], Section 7.2.1)

The following procedures will be conducted for both cohorts:

- Obtain written informed consent (only for patients in Cohort 1)
- Assess inclusion/exclusion criteria (Section 7.2)
- Document concomitant medications
- Physical examination (Section 10.2.5) and vital signs (Section 10.2.6)
- Skin examination (Section 10.2.5)
- Albireo PRO/ObsRO eDiary training, compliance requirements, and instructions to continue recording of pruritus (Appendix 2)
- Clinical chemistry, hematology, and urinalysis (Section 10.2.1)
- s-BA (Section 9.2.1)
- p-C4 and autotaxin
- Alfa-fetoprotein (AFP)
- Vitamins A, E, and 25-hydroxy vitamin D (Section 10.2.1)
- Abdominal ultrasound (Section 10.2.7)
- Fibroscan, where available (Section 9.2.8)
- Administer QoL (Pediatric Quality of Life Inventory [PedsQL]) questionnaire
- Serum pregnancy test for girls who have reached menarche. Please see Appendix 6 for contraceptive requirements (only for patients in Cohort 1)
- Patient/caregiver/clinician complete the patient global impression of symptoms (PGIS) and the patient global impression of change (PGIC)
- AE monitoring

- Study drug is dispensed

Week 4/Visit 2

The following procedures and assessments will be conducted:

- Document concomitant medications
- Vital signs (Section 10.2.6)
- Skin examination (Section 10.2.5)
- Albireo PRO/ObsRO eDiary review for compliance
- Clinical chemistry and hematology (Section 10.2.1)
- INR
- s-BA (Section 9.2.1)
- p-C4 and autotaxin
- Patient/caregiver/clinician complete the PGIS and the PGIC
- Urine pregnancy test for girls who have reached menarche
- AE monitoring
- Evaluation of study drug compliance
- Study drug is dispensed

Week 8/Telephone contact 1

- A study nurse will contact patients via telephone for AE monitoring 4 weeks after Visit 2, at Week 8

Week 12/Visit 3

The following procedures and assessments will be conducted:

- Document concomitant medications
- Physical examination (Section 10.2.5) and vital signs (Section 10.2.6)
- Skin examination (Section 10.2.5)
- Albireo PRO/ObsRO eDiary review for compliance
- Clinical chemistry and hematology (Section 10.2.1)
- INR
- s-BA (Section 9.2.1)
- Vitamins A, E, and 25-hydroxy vitamin D (Section 10.2.1)
- Patient/caregiver/clinician complete the PGIS and the PGIC

- Urine pregnancy test for girls who have reached menarche
- AE monitoring
- Evaluation of study drug compliance; dosage form acceptability questions will be asked of the caregiver and/or patient
- Study drug is dispensed

Week 18/Telephone contact 2

- A study nurse will contact patients via telephone for AE monitoring 6 weeks after Visit 3, at Week 18

Weeks 22 and 46/Visits 4 and 7

The following procedures and assessments will be conducted:

- Document concomitant medications
- Vital signs (Section 10.2.6)
- Albireo PRO/ObsRO eDiary review for compliance
- INR
- s-BA (Section 9.2.1)
- Vitamins A, E, and 25-hydroxy vitamin D (Section 10.2.1)
- Urine pregnancy test for girls who have reached menarche
- AE monitoring

Weeks 24 and 48/Visits 5 and 8

The following procedures and assessments will be conducted:

- Document concomitant medications
- Skin examination (Section 10.2.5)
- Physical examination at Visit 5 (Section 10.2.5) and vital signs (Section 10.2.6)
- Albireo PRO/ObsRO eDiary review for compliance
- Clinical chemistry, hematology, and urinalysis (Section 10.2.1)
- s-BA (Section 9.2.1)
- p-C4 and autotaxin
- AFP
- Abdominal ultrasound (Section 10.2.7)
- Fibroscan, where available (Section 9.2.8)

- Administer PedsQL questionnaire
- Patient/caregiver/clinician complete the PGIS and the PGIC
- Urine pregnancy test for girls who have reached menarche
- AE monitoring
- Evaluation of study drug compliance
- Study drug is dispensed

Weeks 30 and 54/Telephone contacts 3 and 5

- A study nurse will contact patients via telephone for AE monitoring 6 weeks after Visits 5 and 8, at Weeks 30 and 54, respectively

Weeks 36 and 60/Visits 6 and 9

The following procedures and assessments will be conducted:

- Document concomitant medications
- Vital signs (Section 10.2.6)
- Albireo PRO/ObsRO eDiary review for compliance
- Clinical chemistry and hematology (Section 10.2.1)
- INR
- Vitamins A, E, and 25-hydroxy vitamin D (Section 10.2.1)
- s-BA (Section 9.2.1)
- Urine pregnancy test for girls who have reached menarche
- AE monitoring
- Evaluation of study drug compliance; dosage form acceptability questions will be asked of the caregiver and/or patient (only at Visit 6)
- Study drug is dispensed

Weeks 42 and 66/Telephone contacts 4 and 6

- A study nurse will contact patients via telephone for AE monitoring 6 weeks after Visits 6 and 9, at Weeks 42 and 66, respectively

Week 70/Visit 10

The following procedures and assessments will be conducted:

- Document concomitant medications
- Vital signs (Section 10.2.6)

- Albireo PRO/ObsRO eDiary review for compliance
- s-BA (Section 9.2.1)
- INR
- Vitamins A, E, and 25-hydroxy vitamin D (Section 10.2.1)
- Urine pregnancy test for girls who have reached menarche
- AE monitoring

7.1.3.3 End of Treatment/Early Termination/Start of Optional Extension Period

Week 72/Visit 11

The last dose of study drug will be administered in the morning the day before the visit and the following assessments will be performed:

- Document concomitant medications
- Physical examination (Section 10.2.5) and vital signs (Section 10.2.6)
- Skin examination (Section 10.2.5)
- Albireo PRO/ObsRO eDiary review for compliance
- Clinical chemistry, hematology, and urinalysis (Section 10.2.1)
- s-BA (Section 9.2.1)
- p-C4 and autotaxin
- AFP
- Abdominal ultrasound (Section 10.2.7)
- Fibroscan, where available (Section 9.2.8)
- Administer PedsQL questionnaire
- Urine pregnancy test for girls who have reached menarche
- Patient/caregiver/clinician complete the PGIC and PGIS
- AE monitoring
- Evaluation of study drug compliance; dosage form acceptability questions will be asked of the caregiver and/or patient
- Liver biopsy (if required; see Section 9.2.10)
- Patient/Caregiver decide whether to continue A4250 treatment
- Dispensing of drug for optional Extension Period (if applicable)

7.1.3.4 Follow-up Visit

Week 76/Visit 12 (Patients/Caregivers not participating in Optional Extension Period)

Patients will return to the study site 28 days after Visit 11 or the last dose of study drug (for those patients who prematurely withdrawal) for the following assessments:

- Document concomitant medications
- Vital signs (Section 10.2.6)
- Albireo PRO/ObsRO eDiary review for compliance
- Clinical chemistry and hematology (Section 10.2.1)
- INR
- s-BA (Section 9.2.1)
- Urine pregnancy test for girls who have reached menarche
- AE monitoring

7.1.3.5 Optional Extension Period

- Week 88 and every 16 weeks thereafter up to commercial availability of drug
- Document concomitant medications
- Vital signs (Section 10.2.6)
- Clinical Chemistry (Section 10.2.1)
- S-BA (Section 9.2.1)
- Vitamins A, E, and 25-hydroxy vitamin D (Section 10.2.1)
- INR
- Urine pregnancy test for girls who have reached menarche
- Administer PedsQL questionnaire
- Patient/caregiver/clinician complete the PGIS and the PGIC
- Evaluation of study drug compliance
- Study drug is dispensed
- AE monitoring

7.1.3.6 Optional Extension Period End of Treatment

- Document concomitant medications
- Vital signs (Section 10.2.6)
- Clinical Chemistry (Section 10.2.1)

- S-BA (Section 9.2.1)
- Vitamins A, E, and 25-hydroxy vitamin D (Section 10.2.1)
- INR
- Urine pregnancy test for girls who have reached menarche
- Administer PedsQL questionnaire
- Patient/caregiver/clinician complete the PGIS and the PGIC
- Evaluation of study drug compliance
- AE monitoring

7.2 Study Population

Cohort 1 will consist of approximately 60 children who completed 24 weeks, or who withdrew after a minimum of 12 weeks of treatment due to intolerable symptoms in Study A4250-005 and meeting all inclusion criteria and no exclusion criteria are eligible for this study. Patients who withdraw from A4250-005 due to a study drug related AE will not be eligible.

Cohort 2 will consist of approximately 60 patients with any type of PFIC who have elevated s-BAs and cholestatic pruritus and who either (1) do not meet eligibility criteria for Study A4250-005 (PEDFIC 1) or (2) do meet the eligibility criteria for A4250-005 after recruitment of study A4250-005 has been completed. Up to 40 patients post biliary diversion surgery are allowed to participate in Cohort 2.

7.2.1 Inclusion Criteria

Cohort 1:

1. Completion of the 24-week Treatment Period of Study A4250-005 or withdrawn from Study A4250-005 due to patient/caregiver judgment of intolerable symptoms after completing at least 12 weeks of treatment. Patients who withdraw from A4250-005 due to a study drug related AE will not be eligible
2. Signed informed consent and assent as appropriate. Patients who turn 18 years of age (or legal age per country) during the study will be required to re-consent to remain on the study
3. Patients expected to have a consistent caregiver for the duration of the study
4. Caregivers (and age appropriate patients) must be willing and able to use an eDiary device as required by the study

Cohort 2:

1. A male or female patient of any age, with a clinical diagnosis of PFIC and with a body weight ≥5 kg at Visit S-1

2. Patient must have clinical genetic confirmation of PFIC.
3. Patient must have elevated s-BA concentration, specifically measured to be ≥100 µmol/L, taken as the average of 2 samples at least 7 days apart (Visits S-1 and S-2) prior to the Screening/Inclusion Visit (Visit 1)
4. Patient must have history of significant pruritus and a caregiver-reported observed scratching or patient-reported itching (for patients >18 with no caregiver-reported observed scratching) in the eDiary average of ≥2 (on 0 to 4 scale) in the 2 weeks prior to the Screening/Inclusion Visit (Visit 1)
5. Patient and/or legal guardian must sign informed consent (and assent) as appropriate. Patients who turn 18 years of age (or legal age per country) during the study will be required to re-consent in order to remain in the study
6. Age appropriate patients are expected to have a consistent caregiver for the duration of the study
7. Caregivers and age-appropriate patients (≥8 years of age, if able) must be willing and able to use an eDiary device as required by the study

7.2.2 Exclusion Criteria

Patients meeting any of the following criteria at Visit 1 or Visit S-1 will not be eligible for study participation:

Cohort 1:

1. Decompensated liver disease: coagulopathy, history, or presence of clinically significant ascites, variceal hemorrhage, and/or encephalopathy
2. Sexually active males and females who are not using a reliable contraceptive method with ≤1% failure rate (such as hormonal contraception, intra-uterine device, or complete abstinence) throughout the duration of the study and 90 days thereafter (from signed informed consent through 90 days after last dose of study drug). See Appendix 6 for further details
3. Patients not compliant with treatment in study A4250-005
4. Any other conditions or abnormalities which, in the opinion of the investigator or Medical Monitor, may compromise the safety of the patient, or interfere with the patient participating in or completing the study

Cohort 2:

1. Known pathologic variations of the ABCB11 gene that have been demonstrated to result in complete absence of the BSEP protein
2. Patient with past medical history or ongoing presence of other types of liver disease including, but not limited to, the following:

a) Biliary atresia of any kind
b) Benign recurrent intrahepatic cholestasis, indicated by any history of normal s-BAs
c) Suspected or proven liver cancer or metastasis to the liver on imaging studies
d) Histopathology on liver biopsy is suggestive of alternate non-PFIC related etiology of cholestasis

3. Patient with a past medical history or ongoing presence of any other disease or condition known to interfere with the absorption, distribution, metabolism (specifically bile acid metabolism), or excretion of drugs in the intestine, including but not limited to, inflammatory bowel disease
4. Patient with past medical history or ongoing chronic (i.e., >3 months) diarrhea requiring intravenous fluid or nutritional intervention for treatment of the diarrhea and/or its sequelae
5. Patient has a confirmed past diagnosis of infection with human immunodeficiency virus or other present and active, clinically significant, acute, or chronic infection, or past medical history of any major episode of infection requiring hospitalization or treatment with parenteral anti-infective treatment within 4 weeks of treatment start (Study Day 1) or completion of oral anti-infective treatment within 2 weeks prior to start of Screening Period
6. Any patient with suspected or confirmed cancers except for basal cell carcinoma, and non-liver cancers treated at least 5 years prior to Screening with no evidence of recurrence
7. Patient has had a liver transplant, or a liver transplant is planned within 6 months of the Screening/Inclusion Visit
8. Decompensated liver disease, coagulopathy, history, or presence of clinically significant ascites, variceal hemorrhage, and/or encephalopathy
9. INR >1.4 (the patient may be treated with Vitamin K intravenously, and if INR is ≤1.4 at resampling the patient may be randomized)
10. Serum ALT >10 × upper limit of normal (ULN) at Screening
11. Serum ALT >15 × ULN at any time point during the last 6 months unless an alternate etiology was confirmed for the elevation
12. Total bilirubin >10 × ULN at Screening
13. Patient suffers from uncontrolled, recalcitrant pruritic condition other than PFIC. Examples include, but not limited to, refractory atopic dermatitis or other primary pruritic skin diseases
14. Any patient who is pregnant or lactating or who is planning to become pregnant within 72 weeks of the Screening/Inclusion Visit

15. Sexually active males and females who are not using a reliable contraceptive method with ≤1% failure rate (such as hormonal contraception, intrauterine device, or complete abstinence) throughout the duration of the study and 90 days thereafter (from signed informed consent through 90 days after last dose of study drug). See Appendix 6 for further details
16. Patient with a past medical history of alcohol or substance abuse will be excluded. Patient must agree to refrain from illicit drug and alcohol use during the study
17. Administration of bile acid or lipid binding resins and medications that slow GI motility (Refer to Appendix 1 – Concomitant Medications Guidelines)
18. Patient has had investigational exposure to a drug, biologic agent, or medical device within 30 days prior to Screening, or 5 half-lives of the study agent, whichever is longer
19. Any other conditions or abnormalities which, in the opinion of the investigator or Medical Monitor, may compromise the safety of the patient, or interfere with the patient participating in or completing the study

7.2.3 Withdrawal of Patients

Patients/caregivers will be informed that they have the right to withdraw from the study at any time, without prejudice to their medical care, and that they are not obliged to state their reasons.

Any withdrawal, and reasons for withdrawal, must be fully documented in the eCRF and source documents and the patient followed by the investigator/investigative staff. Withdrawn patients will not be replaced.

Patients will be withdrawn in the following circumstances:

- A patient's/caregiver's desire for withdrawal for any reason
- Lost to follow-up (every effort must be made to contact the patient/caregiver; a certified letter must be sent)
- An AE which, in the opinion of the investigator, necessitates withdrawal
- Death
- A patient's/caregiver's substantial non-compliance (eDiary and study drug compliance) or protocol violation
- An investigator's opinion that continuing the patient in the study is not appropriate. The investigator may withdraw a patient at any time, if it is considered to be in the patient's best interest
- Liver transplantation

The reason and the date the patient is withdrawn from the study will be documented in the eCRF and source documents. If a patient is withdrawn from further treatment with the study

drug, the investigator/investigative staff will attempt to complete all Visit 11 procedures at the time the patient is prematurely withdrawn from the study. The investigator/investigative staff will also attempt to complete all Visit 12 procedures 28 days following premature withdrawal (i.e., 28 days following the final dose of the study drug).

If a patient discontinues prior to Week 72, additional telephone contacts will be made every 3 months after the Follow-up Visit (Visit 12) up to Week 72 to gather whether or not the patient has had biliary diversion or liver transplantation.

7.2.4 Study Termination by Sponsor

This study may be terminated at any time by Albireo if significant safety concerns develop or, in the sponsor's judgment, there are no further benefits to be achieved from continuation of the study. In this event, Albireo/designee will inform the study investigators, institutions and all regulatory authorities.

Albireo may temporarily or permanently discontinue the study at an investigative site at any time for safety, ethical, compliance or other reasons. If this is necessary, Albireo will endeavor to provide advance notification to the site. If a site or the study is suspended or discontinued, the investigator/investigative staff will be responsible for promptly informing the Independent Ethics Committee (IEC) that this has happened. If required by local regulations, Albireo/designee will be responsible for informing the IEC and the Regulatory Authority of study or site discontinuation. In such an event, all study data and unused study drug must be returned to Albireo.

8 TREATMENT OF PATIENTS

8.1 Identity of Study Drug

A4250 will be supplied as capsules for oral administration. White opaque capsules filled with pellets containing A4250 will be provided. Two different capsule sizes will be available:

- Capsule size 0 that can be opened
- Capsule size 3 that should be swallowed intact. The size 3 capsules may be opened only under exceptional circumstances e.g., patient cannot swallow the capsule intact.

Bottles with 34 capsules will be given to the patient at each visit. A patient who requires 2 or more capsules per day will be given multiple bottles. Refer to the Investigational Product Manual.

A 5-digit study drug number will identify study drug packs and will be detailed on the study drug label. Dispensing of study drug will be coordinated by the Interactive Web Response System (IWRS). The system will assign study drug number(s) using a packing list based on the patient's weight and dose level (either 120 µg/kg/day or 40 µg/kg/day) for dispensation at each dispensing visit. In case of technical issues accessing the system online, please see the IWRS site user manual for country specific contact telephone numbers to the [redacted] HelpDesk 24/7 system support.

8.2 Administration of Study Drug

Patients will be dosed with 120 µg/kg/day for 72 weeks. Patients not tolerating the dose after a minimum of 1 week, for reasons other than new liver findings and severe diarrhea, will have the option to down-titrate to a lower dose (40 µg/kg/day). They should return to the higher dose as soon as deemed appropriate by the investigator. More than one upward dose titration (from 40 µg/kg/day directly to 120 µg/kg/day) for the same event is not recommended. If, in the opinion of the investigator, a dose titration should be considered prior to the 1 week minimum, the investigator should consult with the Medical Monitor or designee. Any dose titration should be done in consultation with the sponsor Medical Monitor or designee.

Study drug will be dispensed to the patient at defined intervals from Visit 1 through Visit 9, together with instructions on how to store and take the drug. Study drug administration data, including whether each patient took each dose or partial doses of study drug, whether there were any delayed or missed doses, and whether the capsule was opened or swallowed whole, will be documented through the diaries and transferred to the study database.

Patients participating in the optional extension period will be dosed with 120 µg/kg/day. Patients not tolerating the dose at any time throughout the extension period will have the option to down-titrate to a lower dose (40 µg/kg/day) following consultation with the sponsor Medical Monitor or designee. Patients should return to the higher dose as soon as deemed appropriate

by the investigator. More than one upward dose titration (from 40 µg/kg/day directly to 120 µg/kg/day) for the same event is not recommended.

A4250 should be taken in the morning together with food. On clinic visits days when laboratory assessments are conducted (Visits 1 to10; or Visits 1 to XX for those participating in the optional extension period), study drug should be taken after the visit. Patients should not crush or chew the capsule(s). When swallowing the capsule intact, the patient should administer the dose with a glass of water.

If the study medicine needs to be mixed in a small amount of food because the patient is unable to swallow the capsule intact, the capsule can be twisted open and the contents sprinkled and mixed in a small amount (one or two tablespoons (15 to 30 mL)) of room-temperature yogurt, apple sauce, oatmeal, or fruit purée. If the patient has not yet been weaned onto solid foods, the capsule contents can be sprinkled into a small amount (a few milliliters) of water, baby formula or breast milk and administered with an oral dosing pipette. Please see Pharmacy manual for detailed instructions.

If a patient's weight changes at any time during the study, dose adjustment will be required. The number of capsules provided to the patient should be based on the body weight thresholds identified in Table 3.

Table 3 Dosing and Capsule Strength

Body Weight (kg)	Capsule Size	Number of Capsules per Day	Capsule Strength, Low Dose (µg)	Total Dose (µg)	Capsule Strength, High Dose (µg)	Total Dose (µg)
5 to <7.5	0	1	200	200	600	600
7.5 to <12.5	0	2	200	400	600	1200
12.5 to <17.5	0	3	200	600	600	1800
17.5 to <19.5	0	4	200	800	600	2400
19.5 to <25.5	3	2	400	800	1200	2400
25.5 to <35.5	3	3	400	1200	1200	3600
35.5 to <45.5	3	4	400	1600	1200	4800
45.5 to 55.5	3	5	400	2000	1200	6000
>55.5	3	6	400	2400	1200	7200

8.3 Study Drug Packaging and Labelling

8.3.1 Packaging and Labelling

The capsules will be packed in high-density polyethylene containers, with childproof polypropylene caps. Study drug capsules containing A4250 in the strength specified in the table above will be manufactured.

Packaging and labelling will be prepared to comply with applicable regulatory requirements.

8.3.2 Storage

Treatment packs containing A4250 capsules should be stored and dispensed in accordance with regulations in their original containers. The storage facility at the investigative site should be locked and storage should be between 15°C and 25°C.

Patients/caregivers should be informed of appropriate storage conditions (i.e., room temperature, between 15°C and 25°C).

Any deviations from the recommended storage conditions should be immediately reported to Albireo and the study drug should not be used until authorization has been given by Albireo.

8.3.3 Blinding and Randomization of Study Drug

Not applicable.

8.4 Procedure for Breaking the Randomization Code

Not applicable.

8.5 Patient Compliance

The study nurse will monitor eDiary compliance by routine review of the CRF Health website. Patient/caregivers will be instructed to fill in the eDiary every morning and evening for the first 24 weeks and the last 21 days before all the remaining visits to the clinic. There will be no interruption between Visits 11 and 12. Patients participating in the optional extension period will stop eDiary entries at Visit 11. If both diary entries on a day are missing during this time, the study nurse will call the caregiver/patient to remind them to complete all scheduled entries. Any non-compliance will be documented and explained in the source documents.

Study drug compliance will be assessed by review of returned unused medication and by counting returned capsules at Visits 2 through 11 or Visits 2 through XX if participating in the optional extension phase, inclusive. Study drug compliance will be calculated for each patient and listed.

Treatment Compliance = 100 × ((Number of study drug dispensed - number of study drug returned)/number of study drug that should be taken)

Treatment compliance between 80% and 120% will be acceptable.

8.6 Patient Identification

After written informed consent is obtained from an eligible patient, an 8-digit patient identification number will be assigned. [redacted] This number will be captured by the IWRS, when the patient first enters the system in A4250-008. Cohort 1 patients will be assigned the same patient number as used in the A4250-005 study, while Cohort 2 patients will be assigned a new unique patient number.

8.7 Study Drug Accountability

Records shall be maintained of the delivery of study treatment to the study site(s), the inventory at the study site(s), the use of each patient and the return to Albireo.

These records shall include dates, quantities, batch numbers, expiry dates and the unique code numbers assigned to the study drug and to the study patients.

The investigator will be responsible for ensuring that the records adequately document that the patients were provided the quantities specified in the protocol and that all study drug received from Albireo is reconciled.

8.8 Concomitant Therapy and Prohibited Medications

For Cohort 1 patients, the investigator will note all ongoing medication in the eCRF at Visit 1. For Cohort 2 patients, the investigator will note all ongoing medication and any medication recently stopped (within 1 month prior to Visit S-1) in the eCRF at Visit S-1. At Visits 2 to 12, (and S-2 for Cohort 2) and visits in the optional extension period, all changes in medication (stopping or starting new medication or changes in dose) will be recorded in the eCRF. All medication (prescribed or over the counter) for pruritus, sleep, and vitamin supplementation will be recorded.

All medications still being taken by a patient on or after first intake of study drug and which continue to be taken during the study are regarded as concomitant medication.

Concomitant medication guidelines are listed in Appendix 1.

9 ASSESSMENT OF EFFICACY

9.1 Efficacy Endpoints

9.1.1 Primary Efficacy Endpoints

The primary efficacy endpoints are:

EU and rest of world: Change from baseline in s-BA after 72 weeks of treatment.

US: Proportion of positive pruritus assessments at the patient level over the 72-week treatment period using the Albireo ObsRO instrument.

9.1.2 Secondary Efficacy Endpoints

EU and rest of world: Proportion of positive pruritus assessments at the patient level over the 72-week treatment period using the Albireo ObsRO instrument.

US: Change from baseline in s-BA after 72 weeks of treatment.

All Regions:

- Change from baseline in s-BA at Weeks 4, 12, 22, 24, 36, 46, 48, 60, 70, 72, and 76
- Proportion of individual assessments meeting the definition of a positive pruritus assessment at the patient level using the Albireo ObsRO instrument from Weeks 0-4, Weeks 0-12, Weeks 0-22, Weeks 0-24, Weeks 0-36, Weeks 0-46, Weeks 0-48, Weeks 0-60, and Weeks 0-70, respectively, or the proportion of positive pruritus assessments at each 4-week interval between Visit 1/Screening and Visit 5/Week 24, then by each visit between Visit 5/Week 24 to Visit 12/Week 76
- Proportion of individual AM assessments meeting the definition of a positive pruritus assessment at the patient level using the Albireo ObsRO instrument from Weeks 0-4, Weeks 0-12, Weeks 0-22, Weeks 0-24, Weeks 0-36, Weeks 0-46, Weeks 0-48, Weeks 0-60, Weeks 0-70, and Weeks 0-72, respectively, or the proportion of positive pruritus assessments at each 4-week interval between Visit 1/Screening and Visit 5/Week 24, then by each visit between Visit 5/Week 24 to Visit 12/Week 76
- Proportion of individual PM assessments meeting the definition of a positive pruritus assessment at the patient level using the Albireo ObsRO instrument from Weeks 0-4, Weeks 0-12, Weeks 0-22, Weeks 0-24, Weeks 0-36, Weeks 0-46, Weeks 0-48, Weeks 0-60, Weeks 0-70, and Weeks 0-72, respectively, or the proportion of positive pruritus assessments at each 4-week interval between Visit 1/Screening and Visit 5/Week 24, then by each visit between Visit 5/Week 24 to Visit 12/Week 76
- All-cause mortality, number of patients undergoing biliary diversion surgery or liver transplantation. These parameters will be evaluated separately and together at weeks 24, 48, and 72

- Change in growth from baseline to Weeks 24, 48, and 72 after initiation of A4250 treatment, defined as the linear growth deficit (height/length for age, weight for age, and body mass index [BMI]) compared to the standard growth curve (Z-score, standard deviation [SD] from P50)
- Change in AST to platelet ratio index (APRI) score and Fib-4 score from baseline to Week 72
- Change in pediatric end-stage liver disease (PELD)/model for end-stage liver disease (MELD) score from baseline to Week 72
- Change in use of antipruritic medication at Weeks 24, 48, and 72

9.1.3 Exploratory Efficacy Endpoints

All Exploratory Endpoints will be evaluated at Weeks 24, 48, and 72 unless otherwise noted. Baseline is calculated prior to initiation of A4250 treatment. Exploratory endpoints include:

- Change in serum ALT, GGT, and total bilirubin concentration from baseline to Week 72
- Proportion of individual assessments meeting the definition of a positive pruritus assessment at the patient level over the 72-week Treatment Period. A positive pruritus assessment includes an itch score ≤1, or at least a one-point drop from baseline based on the Albireo PRO instrument; only patients ≥8 years of age will complete the Albireo PRO instrument
- Change from baseline over the 72-week treatment period in sleep parameters measured with the Albireo PRO and ObsRO instruments
- Change from baseline in INR, albumin, liver enzymes, leukocytes, and platelets
- Change from baseline measures of bile acid synthesis (autotaxin, p-C4)
- Assessment of Global Symptom Relief at Weeks 4, 12, 24, 48, and 72 as measured by patient, caregiver, and clinician PGIC items
- Change from baseline by each 4-week interval between Visit 1/Screening and Visit 5/Week 24, then by each visit between Visit 5/Week 24 to Visit 12/Week 76 in patient-reported and observer-reported night time itching and scratching severity scores, respectively
- Change from baseline by each 4-week interval between Visit 1/Screening and Visit 5/Week 24, then by each visit between Visit 5/Week 24 to Visit 12/Week 76 in patient-reported and observer-reported morning time itching and scratching severity scores, respectively
- Change from baseline by each 4-week interval between Visit 1/Screening and Visit 5/Week 24, then by each visit between Visit 5/Week 24 to Visit 12/Week 76 in pooled pruritus score including observer-reported scratching for patients <8 years of age and patient-reported itch severity for patients ≥8 years of age

- Change from baseline by each 4-week interval between Visit 1/Screening and Visit 5/Week 24, then by each visit between Visit 5/Week 24 to Visit 12/Week 76 in additional patient-reported and observer-reported sleep parameters (e.g., tiredness, number of awakenings)
- Change from baseline in PedsQL questionnaire
- Change from baseline in stage of liver fibrosis as assessed by Fibroscan® (where available)
- Change from baseline in stage of liver fibrosis as assessed by post-treatment biopsy (when available)

9.2 Efficacy Assessments

9.2.1 Serum Bile Acids

Blood samples for analysis of fasting s-BA will be drawn at all study visits from Visit 1 (Visit S-1 for Cohort 2) through Visit 12. Fasting s-BAs will also be drawn at all visits during the optional extension period. Patients will fast (only water intake is permissible) for at least 4 hours prior to the collection of samples for s-BA. Exceptions can be made for infants, less than 12 months of age, if unable to fast for the full 4 hours. For any visit at which a bile acid sample result is unreportable, an additional unscheduled visit for a repeat sample collection may be scheduled. Samples will be handled and transported to a central laboratory per instructions in the laboratory manual.

9.2.2 Itching and Sleep Score

Itching, observed scratching, and sleep disturbance will be assessed twice each day via eDiary. Patient/caregivers will be instructed to fill in the eDiary every morning and evening for the first 24 weeks and the last 21 days before all the remaining visits to the clinic. There will be no interruption between Visits 11 and 12. Patients participating in the optional extension period will stop eDiary entries at Visit 11. Patients and/or caregivers will be instructed to complete the eDiary in the morning after the patient wakes and in the evening just before the patient goes to sleep.

The eDiary includes Albireo ObsRO and PRO items. Patients <8 years of age will not be asked to complete the Albireo PRO items; only the Albireo ObsRO will be completed by caregivers of patients in this age group. Older patients, ≥8 years of age, will complete the Albireo PRO items and the caregiver will complete the Albireo ObsRO items. The Albireo PRO items assess severity of itch, aspects of sleep disturbance (morning diary only), and tiredness. For patients 8 to 12 years of age, the caregiver will read the Albireo PRO items along with the child and record the child's response. A guide will be provided to the caregivers that provides standardized explanations of the Albireo PRO items, in case the patient is confused or requires clarification about the meaning of a question. The Albireo ObsRO items assess severity of observed scratching, aspects of observed sleep disturbance (morning diary only), and observed signs of tiredness (evening diary only). The Albireo ObsRO and PRO scratching and itch

severity items use 0 to 4 response scales, where each response is distinguished by a unique facial expression, verbal anchor, number, and color code (Appendix 2).

A daily score AM and PM for the Albireo ObsRO scratching item will be averaged from the 2 ratings for each day. A daily score will be considered missing if both of the daily assessments are missing. A weekly score will be calculated by averaging the daily scratching scores. A weekly score will be considered missing if ≥4 out of 7 days a week of data are missing. For AM and PM baseline, a 14-day daily score of AM and PM prior to the first dose of study medication will be averaged as the baseline score.

For AM baseline, a 14-day AM score prior to the first dose of study medication will be averaged as the baseline AM score. The same approach will be used for PM baseline and to calculate a patient-reported itch severity score.

9.2.3 Growth

Growth will be measured as height (velocity) and weight (Z-score) using a certified weight scale. BMI will be calculated by weight (kg) / height $(m)^2$. Change will be defined as linear growth deficit (weight and BMI for age) compared to a standard growth curve.

9.2.4 Biomarker Samples

Blood samples for p-C4 and autotaxin will be drawn at Visits 1, 2, 5, 8, and 11 (p-C4 and autotaxin will only be drawn for patients with body weight >10 kg). Samples will be treated and transported to a central laboratory per instructions in the laboratory manual.

9.2.5 Change of Antipruritic Medication

Any change of antipruritic medication must be noted in the eCRF.

9.2.6 Quality of Life Questionnaire (PedsQL)

Caregivers and, if applicable, patients will be asked to fill out a QoL questionnaire (PedsQL) at Visits 1, 5, 8, 11 and at all visits of the optional extension period. Details of the questions included on the questionnaire can be found in Appendix 4.

9.2.7 PELD/MELD Score

The PELD score will be calculated for children up to 12 years of age. For patients 13 years or older, the MELD score will be calculated.

PELD Score = 4.80 * ln(total bilirubin) + 18.57 * ln(INR) - 6.87 * ln(albumin) + 4.36 (if patient <1 year: scores for patients listed for liver transplantation before the patient's first birthday continue to include the value assigned for age (<1 year) until the patient reaches the age of 24 months) + 6.67 (if patient has growth failure [<-2 SD])

Laboratory values less than 1.0 will be set tot 1.0 for the calculation of the PELD score.

MELD Score = 9.57 * ln(creatinine) + 3.78 * ln(total bilirubin) + 11.2 * ln(INR) + 6.43

Laboratory values less than 1.0 will be set to 1.0 and serum creatinine values greater than 4.0 mg/dL (equivalent to 353.6 µmol/L) will be set to 4.0 for calculation of the MELD score.

If a patient goes from 11 years of age to 12 between the beginning and end of study, both PELD and MELD scores will be calculated at the first visit after the 12th birthday and move to MELD score. The results should be displayed in one summary table.

9.2.8 Fibroscan®

Where available, Fibroscan® will be performed as per institution standard practice at Visits 1, 5, 8, and 11.

9.2.9 Markers of Fibrosis

APRI score and Fib-4 score will be calculated at Visits 1, 3, 5, 8, and 11.

APRI = [(AST in U/L) / (AST upper limit of normal [ULN] in U/L)] x 100 / (Platelets in 10^9/L)

Fibrosis 4 Score = (Age*AST in U/L) / (Platelets in 10^9/L * √ (ALT in U/L))

9.2.10 Liver Biopsy

If a liver biopsy is performed according to the local regulations or standard of care at any time during A4250-008, the biopsy results should be recorded in the eCRF. If the patient has liver biopsy results collected in study A4250-005 or prior to Week 24 in study A4250-008, the patient will be asked to consent to a follow up liver biopsy, if allowed per local regulations, at Visit 11 unless 2 prior liver biopsy results have been collected with at least 1 year between biopsies. Liver biopsies will not be performed in Spain.

9.2.11 Global Impression of Change and Global Impression of Symptom Measures

Patients, caregivers, and clinicians will complete the PGIC and the PGIS measures at Visits 1, 2, 3, 5, 8, 11, and at all visits of the optional extension period (Appendix 3).

The PGIC items assess change in itch (patient version), scratching (caregiver and clinician versions), and sleep (all versions) since starting the study drug. The PGIS items assess itch (patient version), scratching (caregiver and clinician versions), and sleep (all versions) in the past week. Caregivers and clinicians will complete the PGIC and PGIS for all patients; those patients ≥8 years of age will complete the patient version.

10 ASSESSMENT OF SAFETY

The timing and frequency of safety assessments are described in Section 7.1.2 and Section 7.1.3.

The primary safety analysis for this study will include the incidence of total treatment-emergent adverse events (TEAE) and TEAEs categorized by causality, severity, and seriousness assessments.

Trends in safety will also be evaluated for the following assessments:

- Physical examinations
- Concomitant medications
- Vital signs
- Laboratory test results (including clinical chemistry, hematology, urinalysis, AFP, vitamins A, E, and 25-hydroxy vitamin D, and INR)
- Abdominal ultrasound
- Discontinuations due to AEs

10.1 Adverse Events

10.1.1 Definitions and Investigator Assessments

An AE is defined as any untoward medical occurrence in an enrolled patient regardless of causal relationship with study drug. An AE can therefore be any clinically significant unfavorable and unintended sign, symptom, or disease that occurs once a patient is enrolled (ICF is signed) in the study until the patient is discharged from the study, whether or not related to the study drug.

10.1.1.1 Clinical Significance

Clinical significance is defined as any abnormal finding that results in further clinical investigation(s), treatment(s), or the diagnosis of new condition. Patient-reported events and protocol-mandated laboratory values, vital signs, and physical examination findings can be considered clinically significant (i.e., an AE) if there is a deterioration as compared to baseline. Examples of clinically significant worsening from baseline could include, but is not limited to, events causing withdrawal from the study and events requiring medical intervention outside of the study causing apparent clinical manifestations, or judged relevant by the investigator.

10.1.1.2 Serious Adverse Events

Serious criteria are applied by the investigator to each AE as specifically defined below. These criteria are used to determine whether an AE is serious or non-serious. The assessment is made independently of severity assessment (Section 10.1.1.3). For example, the development of a

severe rash that occurs after signing of informed consent may not meet serious criteria as defined below and therefore would be considered a severe, non-serious AE.

Any AE that meets any 1 of the following 6 criteria is considered an SAE:

- The outcome of the AE is **death**
- The AE is immediately **life-threatening**. Life-threatening means that the patient is, in the opinion of the investigator, at immediate risk of death from the reaction as it occurred. This does not include an AE that, if more severe, might have caused death
- The AE results in persistent or significant **disability/incapacity**. Disability means a substantial disruption of a person's ability to conduct normal life functions
- The AE requires or prolongs **hospitalization**
- The AE results in a **congenital anomaly/birth defect**
- **The AE is an important medical event**. Important medical events may meet serious criteria should the investigator assess that they may significantly jeopardize the patient, represent a significant hazard, or requires medical/surgical intervention to prevent one of the outcomes listed above. Examples of potential SAEs based on this criterium include, but are not limited to, allergic bronchospasm requiring intensive treatment in an emergency room or at home, blood dyscrasias or convulsions even if they do not result in inpatient hospitalization, or the development of drug dependency and drug abuse.

10.1.1.3 Severity Assessment

Severity assessments are based on the intensity of the event in relation to expectation. The investigator will assess the intensity of AEs based on the following definitions:

- Mild (awareness of sign or symptom, but easily tolerated)
- Moderate (discomfort sufficient to cause interference with normal activities)
- Severe (incapacitating, with inability to perform normal activities)

Severity is a measure of intensity where seriousness is defined by the criteria outlined in Section 10.1.1.2. An AE of severe intensity need not necessarily be considered serious. For example, nausea that persists for several hours may be considered severe nausea, but not meet serious criteria, and therefore would be assessed as a severe AE but not an SAE.

10.1.1.4 Causality Assessment

The investigator determines the causality of all AEs to the study drug using medical judgment and considering all relevant factors such as (but not limited to) the underlying study indication, coexisting disease, concomitant medication, relevant history, pattern of the AE, temporal relationship to the study drug, and de-challenge or re-challenge. The causality assessment of the AE/SAE is to be made as follows.

Related to study drug (possibly, probably, or definitely related)

Based on medical judgment, there is at least a reasonable possibility that the study drug caused the event; one or more of the following criteria apply:

- The event follows a reasonable temporal sequence from administration of study drug
- The event could not be reasonably attributed to the known characteristics of the patient's clinical state, environmental or toxic factors, or other modes of therapy administered to the patient
- The event follows a known pattern of response to study drug
- The event disappears or decreases on cessation or reduction in dose of the study drug. (It should be noted that in some situations an AE will not disappear or decrease in intensity upon discontinuation of study drug despite other clear indications of relatedness)
- The event reappears or worsens when the study drug is re-administered

Unrelated to study drug (unlikely or unrelated)

Based on medical judgment there is no reasonable possibility that the study drug caused the event; one or more of the following criteria apply:

- The event does not follow a reasonable temporal sequence from administration of study drug
- The event could be reasonably attributed to the known characteristics of the patient's clinical state, concurrent illness, environment or toxic factors, or other modes of therapy administered to the patient
- The event does not follow a known pattern of response to study drug
- The event does not disappear or decrease on cessation or reduction in dose of the study drug, and it does not reappear or worsen when the study drug is re-administered

10.1.2 Recording of Adverse Events

It is the investigator's responsibility to assess whether each untoward event is a clinically significant worsening from baseline, thereby considered an AE. For all AEs, the severity, seriousness, and causality to study drug for each AE as outlined in Section 10.1.1 will be assessed and recorded in the eCRF.

All serious and non-serious AEs are collected once the caregiver/patient has signed the ICF and until the post-treatment follow-up (Visit 12), 28 days after the last dose of study drug. Collection of all serious and non-serious AEs will continue for the optional extension period.

Any AEs or SAEs that are unresolved at the patient's last AE assessment in the study are followed up by the investigator until resolution or stabilization up to the database lock and

recorded in the eCRF. Albireo retains the right to request additional information for any patient with ongoing AE(s) or SAE(s) at the end of the study.

TEAEs are defined as any AE that occurs after dosing (Day 1).

If there is a clinically significant deterioration of a laboratory value/vital sign or other routine study assessment that is associated with a diagnosis, the clinical diagnosis will be reported as an AE and the associated signs and symptoms will be considered additional information unless the sign or symptom is more severe than expected given the diagnosis. For example, if an investigator diagnoses a study patient with hepatitis during the study period, hepatitis would be considered the AE and the concomitant signs and symptoms of abdominal pain, vomiting, and elevated ALT and AST would not be reported separately unless, in the opinion of the investigator, one of these signs or symptoms is more severe than expected and therefore a separate AE assessment is indicated.

10.1.3 Recording and Reporting of Serious Adverse Events

Every SAE (regardless of severity and causality) that occurs once the caregiver/patient has signed the ICF and until the post-treatment follow-up (Visit 12), and 28 days after the final dose of study drug, should immediately and not later than within 24 hours of knowledge of the event, be reported by the investigator or delegate in the SAE Report Form.

Report of a SAE must include at least the following information:

- Patient identification information (study number, site number, initials, and date of birth [as per local country requirements for data protection])
- The last study drug administration date
- The diagnosis of the event with the description (or a brief description of signs/symptoms/clinical course if the diagnosis is not available) and the date of onset
- The action used to treat the event (i.e., treatment medications, temporary discontinuation)
- The reason(s) for considering the event serious
- The relationship of the event to the study drug or to the study procedure (e.g., the investigator's assessment of causality)
- A brief narrative of the SAE

Follow-up reports including all new information obtained of the subsequent course of the SAE must be prepared and the information collected in the SAE Report Form submitted to [redacted] DRUG SAFETY (Pharmacovigilance and Safety Services Department) by e-mail within 24 hours of knowledge of the event.

The pharmacovigilance manager (PVM) may contact the investigator to obtain further information on a reported SAE. The investigator/investigative staff must respond to any

request for follow-up information or answers to questions regarding the SAE within the same timelines as for initial reports.

The PVM reports the occurrence of the SAE and follow-up to the Albireo Medical Monitor for medical assessment of the case.

Should an outcome of death occur within the study period or within 28 days after the last administration of study drug, an SAE Report Form should be completed including the main and contributory causes of death.

All SAE reports must be e-mailed to the following e-mail address within **24 hours**:

[redacted]

If email is unavailable, SAEs may be transmitted via fax to the following number:

[redacted]

In a study-related medical emergency situation, when the assigned Medical Monitors for the study cannot be reached by a caller, an on call physician can be reached 24 hours per day, 7 days per week, via the [redacted] call center. The following number is a chargeable telephone number allowing a global reach from both landlines and mobile phones and the internet page indicated below contains a list of country-specific toll-free telephone numbers. It should be noted that not all countries globally have access to toll-free numbers as indicated on the "24/7 Medical Help desk" index. Countries without toll-free numbers need to dial the chargeable number as indicated below. Furthermore, toll-free numbers might not be available from mobile phones.

On Call Telephone No.: [redacted]

[redacted]

10.1.4 Reporting of Suspected Unexpected Serious Adverse Reactions

Suspected unexpected serious adverse reaction (SUSAR) is an SAE that occurs in a patient, the nature or severity of which is not expected per the applicable product information (e.g., the IB for an unauthorized investigational product or summary of product characteristics for an authorized product).

Reporting and tracking of SUSARs will be in accordance with all applicable competent authority regulations. The Ethics Committees and all investigators involved in this study will be informed according to national and international requirements.

10.2 Laboratory Values/Vital Signs/Physical Examinations and Other Safety Assessments

10.2.1 Laboratory Assessments

At the clinic visits indicated in Table 1, samples will be drawn for clinical chemistry, hematology, and urinalysis analyses. The parameters assessed are presented in Table 4.

Blood for AFP will be drawn at Visits 1, 5, 8, and 11. Fat-soluble vitamin levels including vitamins A and E, and 25-hydroxy vitamin D will be assessed at Visits 1, 3, 4, 6, 7, 9, 10, and all optional extension period visits and INR (surrogate for vitamin K) will be assessed at Visits S-2 (Cohort 2 only), 2, 3, 4, 6, 7, 9, 10, and 12, and all optional extension period visits. Patients will fast (water intake only is permissible) for at least 4 hours prior to the collection of samples for vitamin A. Exceptions can be made for infants, <12 months of age, if unable to fast for the full 4 hours. If a patient has any vitamin level(s) that are outside of the reference range, vitamin supplementation adjustments may be required. Target ranges and supplementation strategy guidelines can be found in Appendix 5.

A serum pregnancy test will be performed at Visits S-1 (Cohort 2 only) and 1, and a urine pregnancy test will be collected at all other visits, including the optional extension period visits, for girls who have reached menarche. If a urine pregnancy test is positive, a serum pregnancy test should be performed to confirm the pregnancy. Study drug administration will be held pending the confirmatory results. If the serum pregnancy test is negative, the patient can resume dosing. If the serum pregnancy test is positive, the patient should be discontinued (see Section 10.2.9).

All samples will be processed and transported to a laboratory per instructions in the laboratory manual.

The observed values will be recorded and assessed as “normal” or “abnormal not clinically significant” or “abnormal clinically significant”.

For hepatic adverse events and/or hepatic decompensation, a PK sample should be collected as close to the onset of the event as possible.

Additional safety blood samples may be needed due to follow-up of an abnormal value or analysis failure. The blood samples collected for safety laboratory analysis will be destroyed after the analyses have been completed.

Table 4 Routine Laboratory Parameters

Clinical Chemistry	Hematology	Urinalysis
• Albumin*<br>• ALT*<br>• Alkaline phosphatase*<br>• AST*<br>• Bilirubin – total and conjugated*<br>• Calcium<br>• Chloride<br>• Creatinine<br>• Creatine kinase<br>• GGT<br>• Potassium<br>• Sodium<br>* Clinical chemistry for optional extension period	• Hematocrit<br>• Hemoglobin<br>• Platelet count<br>• Red blood cell count<br>• White blood cell count and differential (neutrophils, eosinophils, basophils, lymphocytes, and monocytes)	• Blood<br>• Glucose<br>• Ketones<br>• Leukocytes<br>• Nitrites<br>• pH<br>• Protein

10.2.2 Individual Patient Safety Monitoring

10.2.2.1 Liver Monitoring

Strategies to monitor markers of liver disease throughout the study are outlined below where the ULN will be based on central laboratory reference values for age and gender.

For abnormalities classified as hepatic adverse events and/or hepatic decompensation, a PK sample should be collected as close to the onset of the event as possible.

If isolated transaminase elevations are observed, defined as:

1. Normal bilirubin AND absence of clinical hepatitis symptoms AND
   - ALT or AST ≥5 × ULN (if normal at baseline) or an absolute threshold of 800 U/L, whichever comes first
   - OR ALT or AST ≥3 × baseline (if abnormal at baseline) or an absolute threshold of 800 U/L, whichever comes first

Then:

a) Repeat liver profile (AST, ALT, bilirubin, and prothrombin time [PT] or INR) within 2 to 3 days

b) Evaluate creatine phosphokinase (CPK) and lactate dehydrogenase (LDH)

c) As needed (for example, persistent ALT/total bilirubin elevation or worsening of liver function), consider evaluation for alternative etiologies

Monitor the patient using close observation found in Section 10.2.2.2.

If any ONE of the following criteria are met:

1. Transaminases (ALT or AST ≥3 × baseline or 800 U/L, whichever comes first) AND bilirubin (total bilirubin > 2 × ULN) elevations
2. Transaminase elevations alone (ALT or AST >10 × ULN or 5 × baseline or absolute threshold of 800 U/L, whichever comes first) in presence of normal LDH and CPK
3. Total bilirubin increased, unrelated to hemolysis (elevated reticulocyte count) or established genetic diseases, such as Gilbert's syndrome
    a) Doubling if total bilirubin was <3 mg/dL (equivalent to 51.3 µmol/L) at baseline
    b) OR Increase by >3 mg/dL (equivalent to 51.3 µmol/L) if total bilirubin was ≥3 mg/dL (equivalent to 51.3 µmol/L) at baseline
4. INR increase refractory to vitamin K administration
    a) Increase by >1.5 if INR was normal at baseline
    b) OR Increase by >0.4 if INR was abnormal at baseline
5. Any increase in total bilirubin and transaminases if accompanied by EITHER a symptom of clinical hepatitis (e.g., vomiting, nausea, right upper quadrant pain) OR immunological reaction (rash or >5% eosinophilia)

Then:

a) Interrupt study medication
b) Initiate drug-induced liver injury work-up for alternative etiologies
c) Repeat liver profile (AST, ALT, total bilirubin, direct bilirubin) and PT or INR within 48 to 72 hours
d) Monitor the patient using close observation found in Section 10.2.2.2
e) If a patient lives in a remote area, they may be tested locally and the results communicated to the investigator site promptly

10.2.2.2 Close Observation

- Repeat liver enzyme and serum bilirubin tests two or three times weekly. Frequency of re-testing can decrease to once a week or less if abnormalities stabilize or the trial drug has been discontinued and the patient is asymptomatic
- Obtain a more detailed history of symptoms and prior or concurrent diseases
- Obtain a history of concomitant drug use (including non-prescription medications and herbal and dietary supplement preparations), alcohol use, recreational drug use, and special diets

- Consider ruling out alternate etiology including acute viral hepatitis types A, B, C, D, and E; autoimmune or alcoholic hepatitis; non-alcoholic steatohepatitis; hypoxic/ischemic hepatopathy; and biliary tract disease
- Obtain a history of exposure to environmental chemical agents
- Obtain additional tests to evaluate liver function, as appropriate (e.g., INR, direct bilirubin)
- Consider gastroenterology or hepatology consultations.

10.2.2.3 De-challenge/Re-challenge for Liver and Clinical Hepatitis Monitoring

1. Re-challenge is not recommended:
   a) If a patient has had possible/probable drug-induced liver injury
   b) If a decompensation event has occurred (i.e., variceal hemorrhage, ascites, hepatic encephalopathy, etc.).
2. If the event is assessed as due to underlying cholestatic liver disease variability or another alternative etiology is identified AND liver tests returned to baseline, re-challenge may be considered after consultation with the sponsor Medical Monitor.
3. If the ALT/total bilirubin elevations are observed after re-challenge, then repeat re-challenge is discouraged.

If a patient is permanently discontinued, monitoring should be continued as outlined in Section 10.2.2.5.

10.2.2.4 Diarrhea

Study drug should be discontinued if a patient develops diarrhea with at least 1 of the following concomitant signs or symptoms: grossly bloody stools, vomiting, dehydration requiring treatment with oral or intravenous rehydration and/or electrolyte imbalances, fever (≥38°C) and/or the diarrhea persists for 7 or more days.

If there is gross blood in stool, INR and platelets should be measured.

Study drug will be reintroduced (re-challenge) when the symptoms have resolved. If the diarrhea re-occurs within 1 week after the re-challenge with no alternate etiology, the patient will be permanently discontinued and monitored as outlined in Section 10.2.2.5.

10.2.2.5 Monitoring after Permanent Discontinuation of Study Drug Due to Safety

Once study drug is permanently discontinued for liver, hepatitis, diarrhea, or other severe AE related to study drug, the patient will be monitored weekly until the laboratory and clinical parameters have normalized/stabilized.

10.2.3 Demographics/Medical and Surgical History (For Cohort 2 only)

Demographic information per country regulations, along with medical and surgical history, will be obtained and recorded in the eCRF at Visit S-1.

Medical and surgical history will be entered in the eCRF at Visit S-1. This includes date of diagnosis of PFIC, prior investigational medications for PFIC, historical (3 months prior to Screening) LFT values (AST, ALT, and total bilirubin), ongoing medication, any surgery performed, any other diagnosis, and historical biopsy data.

If a liver biopsy has been performed within 1 year prior to Screening, or during the study, the results will be recorded in the eCRF.

10.2.4 Clinical Genetic Testing (For Cohort 2 only)

For previous confirmatory clinical genetic testing results for PFIC Type performed prior to Screening Visit S-1, the confirmatory clinical genetic testing laboratory report will be verified to determine eligibility.

If the historical clinical genetic result is equivocal, unavailable, or unobtainable, clinical genetic analysis will be performed to confirm pathologic biallelic variations of the ATP8B1, ABCB11 ABCB4, NR1H4, TJP2, DCDC2, CLDN1, and MYO5B genes and verified to determine eligibility. No other diagnostic genetic testing will be offered.

10.2.5 Physical Examination

A physician or suitably trained qualified assistant will perform a complete physical examination, including a skin examination, at Visits S-1, 1, 3, 5, 8, and 11.

A complete physical examination will include assessment of general appearance, eyes, ears, nose, throat, head/neck/thyroid, lymph nodes, cardiovascular, lungs/chest, abdomen, genitourinary, extremities, skin, musculoskeletal, neurologic, and other. An abbreviated physical examination will include general appearance, cardiovascular, abdomen, and other assessments based on patient status.

Skin will be thoroughly examined and excoriations/scratch marks recorded.

10.2.6 Vital Signs

Evaluation of vital signs will be performed at all visits. This includes blood pressure (systolic and diastolic), pulse, respiratory rate, temperature, height (or length depending on age) and body weight (using a certified weight scale) at clinic visits. BMI will be calculated.

10.2.7 Abdominal ultrasound

Ultrasound of the liver and spleen will be performed at Visits 1, 5, 8, and 11. Liver size, echogenicity, and presence of masses/nodules as well as spleen size will be recorded. If an ultrasound is performed as standard of care during the optional extension period, the data should be recorded in the electronic case report form (eCRF).

10.2.8 Overdose

A4250 is minimally absorbed and has a very low systemic availability. Based on toxicology data, for study purposes any dose exceeding a total of >3 mg/kg body weight of A4250 taken as a single dose or as a cumulative dose within 24 hours is defined as an overdose.

The no-adverse-effect dose level in the most sensitive species (defined as 20 mg/kg/day) in the rat 1 month toxicity study gives a human equivalent dose of approximately 194 mg/day in a 60 kg person. This dose is approximately 10-fold higher than the maximum possible dose predicted in human studies (20 mg).

The investigator/investigative staff should immediately, and not later than within 24 hours of knowledge, report an overdose in the SAE Report Form. In the event of an A4250 overdose, the patient should be monitored closely.

10.2.9 Pregnancy

If a pregnancy is discovered in a female patient enrolled in the study before the end of dosing the patient will be immediately discontinued from the study and will attend the same visits as a prematurely withdrawn patient. If the pregnancy is discovered after the end of dosing the patient will continue in the study per protocol. If a pregnancy occurs in a male patient´s partner at any time during the study, the pregnancy should also be reported and followed.

Pregnancy is not considered to be an AE. However, if the patient has been dosed with the study drug, the pregnancy must be reported on the Paper Pregnancy Notification Form immediately within 24 hours after recognition to Safety Manager (please refer to Section 10.1.3). Date of exposure and as far as possible, details of the period of gestation at the time of exposure must be given.

The pregnancy should be followed up to determine outcome, including spontaneous termination, details of birth, and presence of any birth defects, congenital anomalies or newborn or maternal complication. An infant who was exposed in-utero will be followed for up to 2 years after delivery. Individual cases with an abnormal outcome in association with the study drug should be reported on an expedited basis, i.e., reported rapidly to a competent authority.

11 STATISTICAL EVALUATION

11.1 Sample Size and Power

There is no formal hypothesis testing in this open label study. Sample size for Cohort 1 was determined in Study A4250-005. For Cohort 1, patients will be enrolled following completion or participation of 12 weeks of Study A4250-005. The expected proportion of patients with at least one event, i.e., surgical bile diversion or liver transplantation or death, in untreated patients is expected to be high enough to be able to detect an improvement after A4250 treatment. For the other endpoints, mainly descriptive analyses will be performed.

The proportion of patients with at least one event (i.e., surgical bile diversion or liver transplantation or death), that can be expected in a non-treated population depends on PFIC type and age distribution of the included patients. The expected proportion of patients with events will be calculated for the study population once each patient's age and PFIC type are known, using the probability that a patient will get an event estimated from the reference population in the ongoing observational cohort study, NAtural Course and Prognosis of PFIC and Effect of Biliary Diversion (NAPPED).

As an example, if the expected proportion with at least one event is estimated to be 30% in the study population, and then if the proportion of patients with at least one event during A4250 long-term treatment are shown to be only 10% in the study, the power is approximately 89% to 96% (1-sided test, alpha=2.5%) for a confidence interval (CI) with upper boundary <30% based on a sample size of n=48 to 60. The sample size of 60 for Cohort 2 was estimated based on the availability of target patient population to evaluate the therapeutic benefit for those patients.

11.2 Statistical Methods

11.2.1 Statistical Analysis Sets

Full Analysis Set

The full analysis set will consist of all patients who received at least 1 dose of study drug in Study A4250-008. The full analysis set will be the primary analysis set for all analyses unless otherwise specified.

11.2.2 Methods of Statistical Analyses

11.2.2.1 General Principles

Descriptive statistics will mainly be used in this open-label extension study. All statistical analyses will be performed using SAS version 9.3 or higher.

The primary analysis will be performed after the last patient (from Cohort 1 or 2) completes the 72-week treatment period. Analyses during the extension period will consist of safety summaries and other evaluations on an ongoing basis per the schedule of assessment for the extension period.

Baseline 1 will be defined as the last value prior to treatment start in Study A4250-005 for Cohort 1 patients.

Baseline 2 will be defined as the last value prior to treatment start in Study A4250-008 for all patients. Baseline 2 will be used in all analyses unless otherwise specified.

Descriptive statistics will be summarized for Cohort 1, Cohort 2, and combined Cohort 1 + Cohort 2. Within Cohort 1, the following subgroups will be presented: 'A4250 40 µg/kg/day (005) to 120 µg/kg/day (008)', 'A4250 120 µg/kg/day (005) to 120 µg/kg/day (008)', 'A4250 40 µg/kg/day (005) and 120 µg/kg/day (005) to 120 µg/kg/day (008)', and 'placebo (005) to 120 µg/kg/day (008)'. Cohort 1 Placebo to A4250 120 µg/kg/day will also be combined with Cohort 2 for presentations.

11.2.2.2 Missing Data

Missing data will be reported descriptively. In general imputations of missing observations will not be made. Any additional sensitivity analysis concerning missingness and details regarding handling of missing data will be described in the SAP.

Continued collection of efficacy data for patients who discontinue treatment will be made as far as possible.

11.2.2.3 Demographic and Baseline Characteristics

For Cohort 1, descriptive summaries of demographics and other baseline characteristics (including medical and surgical history) will be presented overall using the FAS. For Cohort 2, descriptive summaries of demographics and other baseline characteristics will be presented overall for the full analysis set as well as by the underlying diagnostics subgroups.

Prior medication will be summarized overall using the full analysis set.

11.2.2.4 Subject Disposition

The following will be summarized overall by Cohort:

- Patients enrolled (who signed the informed consent)
- Patients treated
- Patients completing the study
- Patients withdrawing early (including withdrawal reason)

Additionally, patients enrolled, included in the FAS will be summarized by region by Cohort.

11.2.2.5 Evaluation of Primary Efficacy Variables

The proportion of positive pruritus assessments at the patient level over the 72-week treatment period will be summarized. A positive pruritus assessment is defined as a scratching score of ≤1 or at least a one-point drop from baseline on the Albireo ObsRO instrument. At each assessment, the AM score will be compared to the baseline AM average,

and the PM score will be compared to the baseline PM average. Both AM and PM pruritus assessments will be included in the analysis of this endpoint. Baseline will be defined as the average score from 14 days prior to treatment start in Study A4250-008 for AM and PM, respectively.

Fasting s-BA baseline will be calculated as the average of the pre-dose value in Study A4250-008 (Visit 1) and the previous visit (Visit 8 in study A4250-005) for Cohort 1. Fasting s-BA baseline will be calculated as the average of Visit S-2 and the pre-dose Visit 1 values in Study A4250-008 for Cohort 2. The end value is the average of the values at Weeks 70 and 72 in study A4250-008.

The change from baseline over the Treatment Period in s-BA will be analyzed mainly by using descriptive statistics. Change over time will also be displayed using graphical presentations (e.g., a spaghetti plot which illustrates the change in both Study A4250-005 and Study A4250-008 by treatment group in Study A4250-005). In addition, change from baseline 1 to end of treatment will be analyzed by using one-sample T-test or Wilcoxon signed rank test as appropriate.

As an exploratory analysis of pruritus and s-BA, a comparison of treatment with A4250 120 μg/kg/day during the first 24 weeks of Study 008 vs placebo patients over the 24-week treatment period from Study 005 will be carried out.

11.2.2.6 Evaluation of Secondary and Exploratory Efficacy Variables

All secondary and exploratory variables listed under Sections 9.1.2 and 9.1.3 will be analyzed descriptively for categorical and continuous data, as applicable. For continuous data, the change from baseline will be analyzed in addition to the actual visit values. For categorical data, shift tables or frequency and percentages of patients will be presented as appropriate.

Change in growth (Z-score, SD from P50 standard growth curve) will be analyzed by using one-sample T-test or Wilcoxon signed rank test as appropriate. Change in growth (height/length for age, weight for age, and BMI) will also be displayed using graphical presentations.

A 95% CI will be calculated for the proportion of patients experiencing surgical bile diversion or liver transplantation or death as well as for the proportion of patients experiencing surgical bile diversion or death or liver transplantation. The upper boundary will be compared to the expected proportion of events, using the event probability estimated from the untreated reference population in an ongoing observational cohort study.

Time to first event i.e., time to surgical bile diversion or liver transplantation or death, will be explored by descriptive statistics and Kaplan-Meier curves, which also will be used to make a comparison to the NAPPED population. The propensity score matched cohort analysis using inverse probability of treatment weights will be used to match the study patient population to the reference population based on age group and PFIC type.

11.2.2.7 Evaluation of Safety Variables

Safety data will be analyzed using descriptive statistics and summaries overall of SAEs, AEs, vital signs, clinical laboratory tests (hematology, clinical chemistry and urinalysis) and concomitant medication. Analyses will be performed using the full analysis set.

Summaries of AEs (coded according to the Medical Dictionary for Regulatory Activities (MedDRA) system organ class and MedDRA preferred term) will include:

- Overview of the incidence of TEAEs (TEAEs, Drug-related TEAEs, TEAEs leading to study discontinuation, and Treatment-emergent SAEs),
- TEAEs by system organ class (SOC) and preferred term,
- Intensity of TEAEs by SOC and preferred term,
- Drug-related TEAEs by SOC and preferred term,
- TEAEs leading to study discontinuation by SOC and preferred term, and
- Treatment-emergent SAEs by SOC and preferred term.

Concomitant medication use during the Treatment Period will be summarized by Anatomical Therapeutic Chemical (ATC) class and World Health Organization (WHO) preferred name.

Summaries of vital signs will be presented. For each visit, the actual results and the change from baseline, and the number and percentage of patients with potentially clinically significant (PCS) values observed post-baseline will be presented.

Summaries of clinical safety laboratory data will be presented. For each visit, the actual result and the change from baseline, and the number and percentage of patients with PCS values observed post-baseline will be presented.

Data listings will be provided for each patient for all safety parameters.

11.2.2.8 Compliance and Exposure

Exposure will be analyzed by calculating the number of days with exposure to study drug. Results will be presented overall using the full analysis set.

The percentage compliance will be described overall, and the number of patients with a compliance <80%, between 80% and ≤120% and >120% will be presented overall.

A summary of patients who down-titrated from 120 µg/kg/day to 40 µg/kg/day during the study will be presented as well.

11.2.3 Interim Analysis

Analyses may be performed at select time points throughout the collection of patient data for regulatory requirements and sponsor decision making purposes.

11.2.4 Data Safety Monitoring Board

A DSMB consisting of sponsor-independent clinical experts and sponsor-independent statistical expert will be established for Study A4250-005, and continue for this study until the last patient completes the initial 72-week treatment period. The DSMB will periodically (approximately on a quarterly basis) meet for the review of accumulating study data, including safety (AE and laboratory data), and liver values.

The DSMB will make recommendations for the remaining part of the study (further details will be provided in the DSMB charter). The DSMB may recommend continuing with the study as planned or stopping the study early for safety reasons. The DSMB will submit its recommendations in writing to Albireo Medical/[redacted] who are responsible for responding to the recommendations of the DSMB and taking appropriate action. The investigators will only be informed by Albireo Medical/[redacted] if the study requires a protocol amendment or is stopped. The DSMB may choose to make additional evaluations at any time if they feel this is warranted from a safety point of view.

The DSMB will act according to its own written standard operating procedure described in a charter and will prepare written minutes of its meetings. The charter of the DSMB will be stored in the Trial Master File. The DSMB will maintain records of its meetings and these will become part of the study file when the study is complete.

12 DIRECT ACCESS TO SOURCE DATA/NOTES

The investigator/institution shall provide direct access to source data/documents for study-related monitoring, audits, IEC/Institutional Review Board (IRB) review and regulatory inspection.

13 QUALITY CONTROL AND QUALITY ASSURANCE

13.1 Conduct of the Study

Albireo/[redacted] shall implement and maintain quality control and quality assurance procedures with written standard operating procedures to ensure that the study is conducted and data are generated, documented, and reported in compliance with the protocol, ICH GCP and applicable regulatory requirements.

This study shall be conducted in accordance with the provisions of the Declaration of Helsinki (October 1996) and all revisions thereof, and in accordance with United States Food and Drug Administration (FDA) regulations (Code of Federal Regulations, Sections 312.50 and 312.56) and with ICH GCP (CPMP 135/95).

The investigator may not deviate from the protocol without a formal protocol amendment having been established and approved by the competent authority, as applicable, and IEC/IRB, except when necessary to eliminate immediate hazards to the patient or when the change(s) involve(s) only logistical or administrative aspects of the study. Any deviations may result in the patient having to be withdrawn from the study and render that patient non-evaluable.

13.2 Study Monitoring

Before study initiation, at a site initiation visit or at an investigator's meeting, an Albireo representative or designee will review the protocol and eCRF with the investigators and the investigative staff. During the study, the clinical monitor (clinical research associate [CRA]) will visit the site regularly to check the completeness of patient records, the accuracy of entries on the eCRFs, the adherence to the protocol and to GCP, the progress of enrollment, and to ensure that study drug is being stored, dispensed, and accounted for according to specifications.

The investigator must ensure that eCRFs are completed within a timely period of the patient visits, as per individual site agreements, and must allow the CRA and Albireo representative or designee periodic access to patient records and all study-related materials, including relevant hospital or clinical records, to confirm their consistency with the eCRF entries. No information in these records about the identity of the patients will leave the study center. Albireo monitoring standards require full verification for the presence of the signed ICF, adherence to the inclusion/exclusion criteria, documentation of SAEs, and recording of primary efficacy and safety variables. The CRA will review source data compared with the eCRFs and will verify source data according to the study-specific monitoring plan. The design of the study, the frequency of patient visits, and the site enrollment rate will determine the frequency of monitoring visits. Upon study completion, the CRA will visit the site to conduct a study termination visit, which will include collection of any outstanding documentation.

It is recommended that the investigator/investigative staff log into the eCRF system every working day or at minimum twice weekly in order to provide a prompt response to queries.

The investigator/investigative staff should respond to queries and make any relevant changes to the study data within 3 working days.

14 ETHICS

14.1 Independent Ethics Committee/Institutional Review Board

Prior to the start of the study, the investigator is responsible for ensuring that the protocol and consent form have been reviewed and approved by a relevant IEC/IRB and competent authority, as applicable. The IEC/IRB shall be appropriately constituted and perform its functions in accordance with FDA ICH GCP and local requirements as applicable.

The IEC/IRB and regulatory authority (competent authority), as applicable, shall approve all protocol amendments (except for logistical or administrative changes), written informed consent documents and document updates, patient recruitment procedures (e.g., advertisements), written information to be provided to the patients, IB, available safety information, information about payment and compensation available to patients and caregivers, the investigator's curriculum vitae and/or other evidence of qualifications and any other documents requested by the IEC/IRB or competent authority as applicable.

14.2 Written Informed Consents and Assents

The investigator (physician) or investigative staff, as according to local regulation, will explain to each patient (or legally authorized representative) the nature of the study, its purpose, the procedures involved, the expected duration, alternative treatment, the potential risks and benefits involved, and any discomfort that may occur. Each caregiver/patient will be informed that participation in the study is voluntary and that he/she or their child may withdraw from the study at any time and that withdrawal of consent will not affect his/her subsequent medical treatment or relationship with the treating physician.

Caregivers/patients will be informed that they/their children may experience side-effects or be at risk for symptoms, illnesses, or complications that cannot be foreseen by Albireo. As with other medications, people treated with A4250 may be at risk of developing allergic reactions or anaphylaxis. Caregivers/patients will be advised that study procedures include regular blood sampling for measurement of safety parameters and biological markers and that some minor risks are associated with these procedures.

This informed consent should be given by means of a signed ICF, written in non-technical language in accordance with ICH GCP, the Declaration of Helsinki, and regulatory authorities. The caregiver(s)/patient should read and consider the statements before signing and dating them and should be given a copy of each signed document. If written consent is not possible, oral consent can be obtained if witnessed and followed by a signed statement from one or more persons not involved in the study, indicating why the patient was unable to sign the form. No patient can enter the study before his/her or caregiver(s), as required by country regulation, informed consent has been obtained.

The ICF is part of the protocol and must be submitted by the investigator/investigative staff with the protocol for IEC approval. Albireo will supply an ICF which complies with regulatory

requirements and country laws and is considered appropriate for the study. Any changes to the ICF suggested by the investigator must be agreed to by Albireo before submission to the IEC and a copy of the approved version must be provided to the clinical monitor after IEC approval.

15 DATA HANDLING AND RECORD KEEPING

15.1 Case Report Forms/Source Data Handling

The investigator shall be provided with standardized eCRFs and shall ensure that all data from patient visits are promptly entered into the eCRFs in accordance with the specific instructions given. The investigator must sign each eCRF to verify the integrity of the data recorded.

A list of the normal ranges for all laboratory tests to be undertaken forms part of the documentation to be collated prior to study start. As a central laboratory has been selected to conduct any or all tests, it is essential that all samples be analyzed at that laboratory.

The investigator must maintain source documents such as laboratory reports, consultation reports, and complete medical history and physical examination reports.

As no ethnicity data can be collected in the source document for study sites in France, the eCRF will be the Source Document for “origines ethniques”.

15.2 Retention of Essential Documents

Essential documents, as defined by ICH GCP, include: the signed protocol and any amendment(s); copies of the completed eCRFs (for site archiving, compact discs of eCRF data for specific patients will be provided); signed ICFs; hospital records and other source documents; IEC approvals and all related correspondence including approved documents; drug accountability records; study correspondence; and a list of patients’ names and addresses.

The investigator/investigative staff must retain copies of these essential documents for the period specified by ICH GCP and by applicable regulatory requirements. The investigator/investigative staff will inform Albireo of the location where the essential documents are stored and must contact Albireo for approval before disposing of any essential documents. The investigator/investigative staff should take measures to prevent accidental or premature destruction of these documents.

16 FINANCING AND INSURANCE

Financing and insurance are addressed in a separate agreement.

17 PUBLICATION POLICY

Albireo will retain the ownership of all data. When the study is complete, Albireo shall arrange the analysis, tabulation of data and preparation of a clinical study report. Albireo may also use the data for publication, presentation at scientific meetings and submission to regulatory authorities. All proposed publications based on this study must be subject to the sponsor's approval requirements.

18 REFERENCE LIST

1. A4250 Investigator’s Brochure.

2. Alissa FT, Jaffe R, Shneider BL. Update on progressive familial intrahepatic cholestasis. J Pediatr Gastroenterol Nutr. 2008;46(3):241-52.

3. Davit-Spraul A, Gonzales E, Baussan C, et al. Progressive familial intrahepatic cholestasis. Orphanet J Rare Disease 2009;4:1-12.

4. Hofmann AF. The enterohepatic circulation of bile acids in mammals: form and functions. Front Biosci (Landmark Ed). 2009 Jan 1;14:2584-98.

5. Hori T, Justin H, Nguyen JH and Uemoto S. Progressive familial intrahepatic cholestasis. Hepatobiliary pancreat Dis Int. 2010; 9(6):570-8.

6. Jacquemin E. Progressive familial intrahepatic cholestasis. Genetic basis and treatment. Clin Liver Dis. 2000 Nov;4(4):753-63.

7. Miethke AG, Zhang W, Simmons J, et al. Pharmacological inhibition of ASBT changes bile composition and blocks progression of sclerosing cholangitis in mdr2 knockout mice. Hepatology. 2016 February; 63(2):512–23.

8. Murray CS, Rees JL. Are subjective accounts of itch to be relied on? The lack of relation between visual analogue itch scores and actigraphic measures of scratch. Acta Derm Venereol. 2011;91(1):18-23.

9. Pawlikowska L, Strautnieks S, Jankowska I. et al. Differences in presentation and progression between severe FIC1 and BSEP deficiencies, J Hepatol. 2010; 53(1):170-8.

10. Suchy FJ. “Approach to the Infant With Cholestasis.” *Liver Disease in Children*. Ed. Frederick J. Suchy. New York: Cambridge University Press; 2007 179-189. Print.

11. Whitington PF, Whitington GL. Partial external diversion of bile for the treatment of intractable pruritus associated with intrahepatic cholestasis. Gastroenterology. 1988;95(1):130-6.

12. Venkat VL, Shneider BL, Magee JC, et al. Total Serum Bilirubin Predicts Fat-Soluble Vitamin Deficiency Better Than Serum Bile Acids in Infants with Biliary Atresia. J Pediatr Gastroenterol Nutr. 2014;59(6):702-7.

13. Shneider BL, Magee JC, Bezerra JA, et al. Efficacy of fat-soluble vitamin supplementation in infants with biliary atresia. Pediatrics, 2012;130(3):e607-14.

19 APPENDICES

Appendix 1 Concomitant Medication Guidelines

Appendix 1: Concomitant Medication Guidelines

From the first day of Screening to the last day of the Treatment Period, no drugs with effects on bile acid concentration in the gastrointestinal (GI) tract or drugs with known effects on GI motility are allowed. Examples of prohibited medications are listed below:

- Bile acid or lipid binding resins
    - Cholestyramine
    - Colesevelam
    - Colestipol
- Medications that slow GI motility
    - Sucralfate
    - Loperamide
    - Codeine
- Prokinetic medications
    - Erythromycin
- Other investigational products
    - 4-Phenylbutyrate

Other drugs/natural products with possible effects on GI motility (e.g., selective serotonin reuptake inhibiting drugs, tetracyclic antidepressants, fiber supplementation, yogurt variants) are allowed provided stable usage of the product at least 4 weeks before enrollment until treatment discontinuation.

Treatment with ursodeoxycholic acid, rifampicin, and/or antihistamines are also allowed provided patient was on stable dosage at least 4 weeks before enrollment. Prior to any dose change during the study, the medical monitor must be consulted.

Topical treatment is allowed without restriction.

Appendix 2 Diary Questions including Albireo Patient-Reported Outcomes/Observer-Reported Outcome Instrument

Appendix 2: Diary Questions including Albireo Patient-Reported Outcomes/Observer-Reported Outcome Instrument

Patient-Reported Outcome

**Morning Diary (to be completed shortly after waking each morning)**	
Please answer the questions on the following screens. There are no right or wrong answers. Please think about the time since you went to bed last night (beginning when you started trying to fall asleep).	
1. How bad was your worst itching since you went to bed last night?	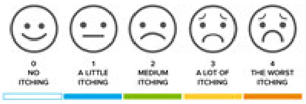
2. How hard was it to fall asleep last night because of your itching?	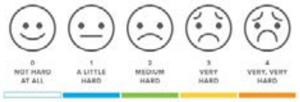
3. How hard was it to stay asleep last night because of your itching?	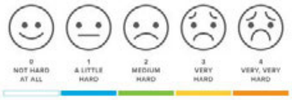
4. Did you wake up last night because of itching?	No / Yes
5. How tired do you feel this morning?	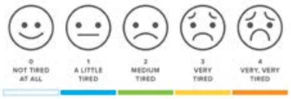

**Bedtime Diary (to be completed when going to bed each night)**	
Please answer the questions on the following screens. There are no right or wrong answers. Please think about the time since you woke up this morning.	
1. How bad was your worst itching since you woke up this morning?	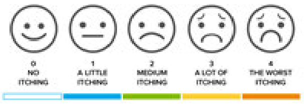
2. How tired were you since you woke up this morning?	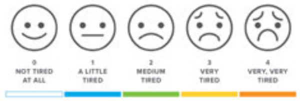

Observer Reported Outcome

**Morning Diary (to be completed shortly after child wakes up each morning)**	
Please answer the questions on the following screens. There are no right or wrong answers. Please think about the time since your child went to bed last night (beginning when your child started trying to fall asleep). As a reminder, when we use the term "scratching," we would like you to think about all of the different types of scratching behaviors that you can see.	
1. How bad was your child's worst scratching since he/she went to bed last night?	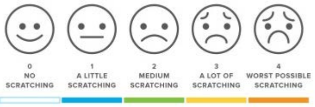
2. Since your child went to bed last night, did you see blood due to scratching?	No / Yes
3. Did your child need a caregiver to help him/her fall asleep last night due to his/her itching?	No / Yes
4. Did your child need a caregiver to soothe him/her at some time during the night last night due to his/her itching?	No / Yes
5. Did your child need a caregiver to sleep with him/her at some time during the night last night due to his/her itching?	No / Yes
6. How many times did you notice that your child woke up last night?	0-99
7. Did your child take any prescribed or over-the-counter medicines before going to bed last night that may have made him/her sleepy?	No / Yes

**Bedtime Diary (to be completed when child is going to bed each night)**	
Please answer the questions on the following screens. There are no right or wrong answers. Please think about the time since your child woke up this morning. As a reminder, when we use the term "scratching," we would like you to think about all of the different types of scratching behaviors that you can see.	
1. How bad was your child's worst scratching since he/she woke up this morning?	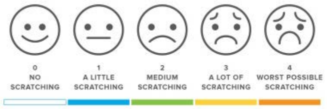
2. How tired did your child seem to be today?	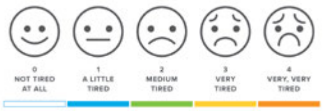

Appendix 3 Patient Global Impression of Change (PGIC) and Patient Global Impression of Symptoms (PGIS)

Appendix 3: Patient Global Impression of Change (PGIC) and Patient Global Impression of Symptoms (PGIS)

ITCH

PGIC – CHILD

Please pick the answer below that best describes the overall change in your itch since you started taking the study medicine.

- ☐ Very much better
- ☐ Much better
- ☐ A little better
- ☐ No change
- ☐ A little worse
- ☐ Much worse
- ☐ Very much worse

PGIC – PARENT

Please choose the response below that best describes the overall change in your child's scratching since he/she started taking the study medication.

- ☐ Very much better
- ☐ Moderately better
- ☐ A little better
- ☐ No change
- ☐ A little worse
- ☐ Moderately worse
- ☐ Very much worse

PGIC – CLINICIAN

Please choose the response below that best describes the overall change in your patient's scratching since he/she started taking the study medication.

- ☐ Very much better
- ☐ Moderately better
- ☐ A little better
- ☐ No change
- ☐ A little worse
- ☐ Moderately worse
- ☐ Very much worse

PGIS – CHILD

Please choose the answer below that best describes how bad your itch has been the past week.

- ☐ None
- ☐ A little
- ☐ Medium
- ☐ Bad
- ☐ Very Bad

PGIS – PARENT

Please choose the response below that best describes the severity of your child's scratching over the past week.

- ☐ None
- ☐ Mild
- ☐ Moderate
- ☐ Severe
- ☐ Very Severe

PGIS – CLINICIAN

Please choose the response below that best describes the severity of your patient's scratching over the past week.

- ☐ None
- ☐ Mild
- ☐ Moderate
- ☐ Severe
- ☐ Very Severe

SLEEP

PGIC – CHILD

Please choose the answer below that best describes the overall change in your sleep problems since you started taking the study medicine.

- ☐ Very much better
- ☐ Much better
- ☐ A little better
- ☐ No change
- ☐ A little worse
- ☐ Much worse
- ☐ Very much worse

PGIC – PARENT

Please choose the response below that best describes the overall change in your child's sleep problems since he/she started taking the study medication.

- ☐ Very much better
- ☐ Moderately better
- ☐ A little better
- ☐ No change
- ☐ A little worse
- ☐ Moderately worse
- ☐ Very much worse

PGIC – CLINICIAN

Please choose the response below that best describes the overall change in your patient's sleep problems since he/she started taking the study medication.

- ☐ Very much better
- ☐ Moderately better
- ☐ A little better
- ☐ No change
- ☐ A little worse
- ☐ Moderately worse
- ☐ Very much worse

PGIS – CHILD

Please choose the answer below that best describes how bad your sleep problems have been over the past week.

- ☐ None
- ☐ A little
- ☐ Medium
- ☐ Bad

- ☐ Very Bad

PGIS – PARENT

Please choose the response below that best describes the severity of your child's sleep problems over the past week.

- ☐ None
- ☐ Mild
- ☐ Moderate
- ☐ Severe
- ☐ Very Severe

PGIS – CLINICIAN

Please choose the response below that best describes the severity of your patient's sleep problems over the past week.

- ☐ None
- ☐ Mild
- ☐ Moderate
- ☐ Severe
- ☐ Very Severe

Appendix 4 Pediatric Quality of Life Inventory (PedQL) Questionnaire

ID# ____________________

Date:___________________

PedsQL™

Pediatric Quality of Life Inventory

Version 4.0

PARENT REPORT for **TODDLERS** (ages **2-4**)

DIRECTIONS

On the following page is a list of things that might be a problem for **your child**. Please tell us **how much of a problem** each one has been for **your child** during the **past ONE month** by circling:

0 if it is **never** a problem
1 if it is **almost never** a problem
2 if it is **sometimes** a problem
3 if it is **often** a problem
4 if it is **almost always** a problem

There are no right or wrong answers.
If you do not understand a question, please ask for help.

 Copyright © 1998 JW Varni, Ph.D. All rights reserved

*In the past **ONE month,** how much of a **problem** has your child had with …*

**PHYSICAL FUNCTIONING *(problems with…)***	Never	Almost Never	Some-times	Often	Almost Always
1. Walking	0	1	2	3	4
2. Running	0	1	2	3	4
3. Participating in active play or exercise	0	1	2	3	4
4. Lifting something heavy	0	1	2	3	4
5. Bathing	0	1	2	3	4
6. Helping to pick up his or her toys	0	1	2	3	4
7. Having hurts or aches	0	1	2	3	4
8. Low energy level	0	1	2	3	4

**EMOTIONAL FUNCTIONING *(problems with…)***	Never	Almost Never	Some-times	Often	Almost Always
1. Feeling afraid or scared	0	1	2	3	4
2. Feeling sad or blue	0	1	2	3	4
3. Feeling angry	0	1	2	3	4
4. Trouble sleeping	0	1	2	3	4
5. Worrying	0	1	2	3	4

**SOCIAL FUNCTIONING *(problems with…)***	Never	Almost Never	Some-times	Often	Almost Always
1. Playing with other children	0	1	2	3	4
2. Other kids not wanting to play with him or her	0	1	2	3	4
3. Getting teased by other children	0	1	2	3	4
4. Not able to do things that other children his or her age can do	0	1	2	3	4
5. Keeping up when playing with other children	0	1	2	3	4

***Please complete this section if your child attends school or daycare**

**SCHOOL FUNCTIONING *(problems with…)***	Never	Almost Never	Some-times	Often	Almost Always
1. Doing the same school activities as peers	0	1	2	3	4
2. Missing school/daycare because of not feeling well	0	1	2	3	4
3. Missing school/daycare to go to the doctor or hospital	0	1	2	3	4

 Not to be reproduced without permission Copyright © 1998 JW Varni, Ph.D. All rights reserved

ID# ______________________

Date:______________________

PedsQL™

Pediatric Quality of Life Inventory

Version 4.0

YOUNG CHILD REPORT (ages **5-7**)

Instructions for interviewer:

I am going to ask you some questions about things that might be a problem for some children. I want to know how much of a problem any of these things might be for you.

Show the child the template and point to the responses as you read.

If it is <u>not at all</u> a problem for you, point to the smiling face

If it is <u>sometimes</u> a problem for you, point to the middle face

If it is a problem for you <u>a lot</u>, point to the frowning face

I will read each question. Point to the pictures to show me how much of a problem it is for you. Let's try a practice one first.

	Not at all	Sometimes	A lot
**Is it hard for you to snap your fingers**	☺	😐	☹

Ask the child to demonstrate snapping his or her fingers to determine whether or not the question was answered correctly. Repeat the question if the child demonstrates a response that is different from his or her action.

 Copyright © 1998 JW Varni, Ph.D. All rights reserved

Think about how you have been doing for the last few weeks. Please listen carefully to each sentence and tell me how much of a problem this is for you.

After reading the item, gesture to the template. If the child hesitates or does not seem to understand how to answer, read the response options while pointing at the faces.

**PHYSICAL FUNCTIONING *(problems with…)***	**Not at all**	**Some-times**	**A lot**
1. Is it hard for you to walk	0	2	4
2. Is it hard for you to run	0	2	4
3. Is it hard for you to play sports or exercise	0	2	4
4. Is it hard for you to pick up big things	0	2	4
5. Is it hard for you to take a bath or shower	0	2	4
6. Is it hard for you to do chores (like pick up your toys)	0	2	4
7. Do you have hurts or aches (***Where?*___________)	0	2	4
8. Do you ever feel too tired to play	0	2	4

Remember, tell me how much of a problem this has been for you for the last few weeks.

**EMOTIONAL FUNCTIONING *(problems with…)***	**Not at all**	**Some-times**	**A lot**
1. Do you feel scared	0	2	4
2. Do you feel sad	0	2	4
3. Do you feel mad	0	2	4
4. Do you have trouble sleeping	0	2	4
5. Do you worry about what will happen to you	0	2	4

**SOCIAL FUNCTIONING *(problems with…)***	**Not at all**	**Some-times**	**A lot**
1. Is it hard for you to get along with other kids	0	2	4
2. Do other kids say they do not want to play with you	0	2	4
3. Do other kids tease you	0	2	4
4. Can other kids do things that you cannot do	0	2	4
5. Is it hard for you to keep up when you play with other kids	0	2	4

**SCHOOL FUNCTIONING *(problems with…)***	**Not at all**	**Some-times**	**A lot**
1. Is it hard for you to pay attention in school	0	2	4
2. Do you forget things	0	2	4
3. Is it hard to keep up with schoolwork	0	2	4
4. Do you miss school because of not feeling good	0	2	4
5. Do you miss school because you have to go to the doctor's or hospital	0	2	4

PedsQL 4.0 - (5-7) Not to be reproduced without permission Copyright © 1998 JW Varni, Ph.D. All rights reserved
01/00
PedsQL-4.0-Core-YC – United States/English – Original version
PedsQL-4 0-Core-YC_AU4.0_eng-USori

How much of a problem is this for you?

Not at all	Sometimes	A lot
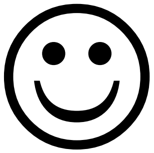	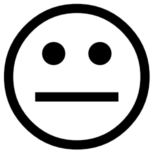	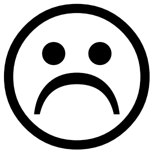

Not to be reproduced without permission
Copyright © 1998 JW Varni, Ph.D. All rights reserved.

ID# ______________________

Date: ______________________

PedsQL™

Pediatric Quality of Life Inventory

Version 4.0

PARENT REPORT for **YOUNG CHILDREN** (ages **5-7**)

DIRECTIONS

On the following page is a list of things that might be a problem for **your child**. Please tell us **how much of a problem** each one has been for **your child** during the **past ONE month** by circling:

0 if it is **never** a problem
1 if it is **almost never** a problem
2 if it is **sometimes** a problem
3 if it is **often** a problem
4 if it is **almost always** a problem

There are no right or wrong answers.
If you do not understand a question, please ask for help.

 Copyright © 1998 JW Varni, Ph.D. All rights reserved

*In the past **ONE month,** how much of a **problem** has your child had with …*

**PHYSICAL FUNCTIONING *(problems with…)***	**Never**	**Almost Never**	**Some-times**	**Often**	**Almost Always**
1. Walking more than one block	0	1	2	3	4
2. Running	0	1	2	3	4
3. Participating in sports activity or exercise	0	1	2	3	4
4. Lifting something heavy	0	1	2	3	4
5. Taking a bath or shower by him or herself	0	1	2	3	4
6. Doing chores, like picking up his or her toys	0	1	2	3	4
7. Having hurts or aches	0	1	2	3	4
8. Low energy level	0	1	2	3	4

**EMOTIONAL FUNCTIONING *(problems with…)***	**Never**	**Almost Never**	**Some-times**	**Often**	**Almost Always**
1. Feeling afraid or scared	0	1	2	3	4
2. Feeling sad or blue	0	1	2	3	4
3. Feeling angry	0	1	2	3	4
4. Trouble sleeping	0	1	2	3	4
5. Worrying about what will happen to him or her	0	1	2	3	4

**SOCIAL FUNCTIONING *(problems with…)***	**Never**	**Almost Never**	**Some-times**	**Often**	**Almost Always**
1. Getting along with other children	0	1	2	3	4
2. Other kids not wanting to be his or her friend	0	1	2	3	4
3. Getting teased by other children	0	1	2	3	4
4. Not able to do things that other children his or her age can do	0	1	2	3	4
5. Keeping up when playing with other children	0	1	2	3	4

**SCHOOL FUNCTIONING *(problems with…)***	**Never**	**Almost Never**	**Some-times**	**Often**	**Almost Always**
1. Paying attention in class	0	1	2	3	4
2. Forgetting things	0	1	2	3	4
3. Keeping up with school activities	0	1	2	3	4
4. Missing school because of not feeling well	0	1	2	3	4
5. Missing school to go to the doctor or hospital	0	1	2	3	4

 Not to be reproduced without permission Copyright © 1998 JW Varni, Ph.D. All rights reserved

ID# ___________________________

Date: ___________________________

PedsQL™

Pediatric Quality of Life Inventory

Version 4.0

CHILD REPORT (ages **8-12**)

DIRECTIONS

On the following page is a list of things that might be a problem for you. Please tell us **how much of a problem** each one has been for you during the **past ONE month** by circling:

0 if it is **never** a problem
1 if it is **almost never** a problem
2 if it is **sometimes** a problem
3 if it is **often** a problem
4 if it is **almost always** a problem

There are no right or wrong answers.
If you do not understand a question, please ask for help.

Copyright © 1998 JW Varni, Ph.D. All rights reserved

*In the past **ONE month**, how much of a **problem** has this been for you …*

**ABOUT MY HEALTH AND ACTIVITIES** ***(problems with…)***	**Never**	**Almost Never**	**Some-times**	**Often**	**Almost Always**
1. It is hard for me to walk more than one block	0	1	2	3	4
2. It is hard for me to run	0	1	2	3	4
3. It is hard for me to do sports activity or exercise	0	1	2	3	4
4. It is hard for me to lift something heavy	0	1	2	3	4
5. It is hard for me to take a bath or shower by myself	0	1	2	3	4
6. It is hard for me to do chores around the house	0	1	2	3	4
7. I hurt or ache	0	1	2	3	4
8. I have low energy	0	1	2	3	4

**ABOUT MY FEELINGS** ***(problems with…)***	**Never**	**Almost Never**	**Some-times**	**Often**	**Almost Always**
1. I feel afraid or scared	0	1	2	3	4
2. I feel sad or blue	0	1	2	3	4
3. I feel angry	0	1	2	3	4
4. I have trouble sleeping	0	1	2	3	4
5. I worry about what will happen to me	0	1	2	3	4

**HOW I GET ALONG WITH OTHERS** ***(problems with…)***	**Never**	**Almost Never**	**Some-times**	**Often**	**Almost Always**
1. I have trouble getting along with other kids	0	1	2	3	4
2. Other kids do not want to be my friend	0	1	2	3	4
3. Other kids tease me	0	1	2	3	4
4. I cannot do things that other kids my age can do	0	1	2	3	4
5. It is hard to keep up when I play with other kids	0	1	2	3	4

**ABOUT SCHOOL** ***(problems with…)***	**Never**	**Almost Never**	**Some-times**	**Often**	**Almost Always**
1. It is hard to pay attention in class	0	1	2	3	4
2. I forget things	0	1	2	3	4
3. I have trouble keeping up with my schoolwork	0	1	2	3	4
4. I miss school because of not feeling well	0	1	2	3	4
5. I miss school to go to the doctor or hospital	0	1	2	3	4

Not to be reproduced without permission

Copyright © 1998 JW Varni, Ph.D. All rights reserved

ID# ______________________

Date:_____________________

PedsQL™

Pediatric Quality of Life Inventory

Version 4.0

PARENT REPORT for **CHILDREN** (ages **8-12**)

DIRECTIONS

On the following page is a list of things that might be a problem for **your child**. Please tell us **how much of a problem** each one has been for **your child** during the **past ONE month** by circling:

0 if it is **never** a problem
1 if it is **almost never** a problem
2 if it is **sometimes** a problem
3 if it is **often** a problem
4 if it is **almost always** a problem

There are no right or wrong answers.
If you do not understand a question, please ask for help.

Not to be reproduced without permission

Copyright © 1998 JW Varni, Ph.D. All rights reserved

*In the past **ONE month,** how much of a **problem** has your child had with …*

PHYSICAL FUNCTIONING *(problems with…)*	Never	Almost Never	Some-times	Often	Almost Always
1. Walking more than one block	0	1	2	3	4
2. Running	0	1	2	3	4
3. Participating in sports activity or exercise	0	1	2	3	4
4. Lifting something heavy	0	1	2	3	4
5. Taking a bath or shower by him or herself	0	1	2	3	4
6. Doing chores around the house	0	1	2	3	4
7. Having hurts or aches	0	1	2	3	4
8. Low energy level	0	1	2	3	4

EMOTIONAL FUNCTIONING *(problems with…)*	Never	Almost Never	Some-times	Often	Almost Always
1. Feeling afraid or scared	0	1	2	3	4
2. Feeling sad or blue	0	1	2	3	4
3. Feeling angry	0	1	2	3	4
4. Trouble sleeping	0	1	2	3	4
5. Worrying about what will happen to him or her	0	1	2	3	4

SOCIAL FUNCTIONING *(problems with…)*	Never	Almost Never	Some-times	Often	Almost Always
1. Getting along with other children	0	1	2	3	4
2. Other kids not wanting to be his or her friend	0	1	2	3	4
3. Getting teased by other children	0	1	2	3	4
4. Not able to do things that other children his or her age can do	0	1	2	3	4
5. Keeping up when playing with other children	0	1	2	3	4

SCHOOL FUNCTIONING *(problems with…)*	Never	Almost Never	Some-times	Often	Almost Always
1. Paying attention in class	0	1	2	3	4
2. Forgetting things	0	1	2	3	4
3. Keeping up with schoolwork	0	1	2	3	4
4. Missing school because of not feeling well	0	1	2	3	4
5. Missing school to go to the doctor or hospital	0	1	2	3	4

 Not to be reproduced without permission Copyright © 1998 JW Varni, Ph.D. All rights reserved

ID#______________________

Date:_____________________

PedsQL™

Pediatric Quality of Life Inventory

Version 4.0

PARENT REPORT for **TEENS** (ages **13-18**)

DIRECTIONS

On the following page is a list of things that might be a problem for **your teen**. Please tell us **how much of a problem** each one has been for **your teen** during the **past ONE month** by circling:

0 if it is **never** a problem
1 if it is **almost never** a problem
2 if it is **sometimes** a problem
3 if it is **often** a problem
4 if it is **almost always** a problem

There are no right or wrong answers.
If you do not understand a question, please ask for help.

Copyright © 1998 JW Varni, Ph.D. All rights reserved

*In the past **ONE month,** how much of a **problem** has your teen had with …*

**PHYSICAL FUNCTIONING** ***(problems with…)***	**Never**	**Almost Never**	**Some-times**	**Often**	**Almost Always**
1. Walking more than one block	0	1	2	3	4
2. Running	0	1	2	3	4
3. Participating in sports activity or exercise	0	1	2	3	4
4. Lifting something heavy	0	1	2	3	4
5. Taking a bath or shower by him or herself	0	1	2	3	4
6. Doing chores around the house	0	1	2	3	4
7. Having hurts or aches	0	1	2	3	4
8. Low energy level	0	1	2	3	4

**EMOTIONAL FUNCTIONING** ***(problems with…)***	**Never**	**Almost Never**	**Some-times**	**Often**	**Almost Always**
1. Feeling afraid or scared	0	1	2	3	4
2. Feeling sad or blue	0	1	2	3	4
3. Feeling angry	0	1	2	3	4
4. Trouble sleeping	0	1	2	3	4
5. Worrying about what will happen to him or her	0	1	2	3	4

**SOCIAL FUNCTIONING** ***(problems with…)***	**Never**	**Almost Never**	**Some-times**	**Often**	**Almost Always**
1. Getting along with other teens	0	1	2	3	4
2. Other teens not wanting to be his or her friend	0	1	2	3	4
3. Getting teased by other teens	0	1	2	3	4
4. Not able to do things that other teens his or her age can do	0	1	2	3	4
5. Keeping up with other teens	0	1	2	3	4

**SCHOOL FUNCTIONING** ***(problems with…)***	**Never**	**Almost Never**	**Some-times**	**Often**	**Almost Always**
1. Paying attention in class	0	1	2	3	4
2. Forgetting things	0	1	2	3	4
3. Keeping up with schoolwork	0	1	2	3	4
4. Missing school because of not feeling well	0	1	2	3	4
5. Missing school to go to the doctor or hospital	0	1	2	3	4

Not to be reproduced without permission
Copyright © 1998 JW Varni, Ph.D. All rights reserved

ID# ______________________________
Date:______________________________

PedsQL ™

Pediatric Quality of Life Inventory

Version 4.0

TEEN REPORT (ages **13-18**)

DIRECTIONS

On the following page is a list of things that might be a problem for you. Please tell us **how much of a problem** each one has been for you during the **past ONE month** by circling:

0 if it is **never** a problem
1 if it is **almost never** a problem
2 if it is **sometimes** a problem
3 if it is **often** a problem
4 if it is **almost always** a problem

There are no right or wrong answers.
If you do not understand a question, please ask for help.

Not to be reproduced without permission

Copyright © 1998 JW Varni, Ph.D. All rights reserved

*In the past **ONE month**, how much of a **problem** has this been for you …*

**ABOUT MY HEALTH AND ACTIVITIES *(problems with…)***	Never	Almost Never	Some-times	Often	Almost Always
1. It is hard for me to walk more than one block	0	1	2	3	4
2. It is hard for me to run	0	1	2	3	4
3. It is hard for me to do sports activity or exercise	0	1	2	3	4
4. It is hard for me to lift something heavy	0	1	2	3	4
5. It is hard for me to take a bath or shower by myself	0	1	2	3	4
6. It is hard for me to do chores around the house	0	1	2	3	4
7. I hurt or ache	0	1	2	3	4
8. I have low energy	0	1	2	3	4

**ABOUT MY FEELINGS *(problems with…)***	Never	Almost Never	Some-times	Often	Almost Always
1. I feel afraid or scared	0	1	2	3	4
2. I feel sad or blue	0	1	2	3	4
3. I feel angry	0	1	2	3	4
4. I have trouble sleeping	0	1	2	3	4
5. I worry about what will happen to me	0	1	2	3	4

**HOW I GET ALONG WITH OTHERS *(problems with…)***	Never	Almost Never	Some-times	Often	Almost Always
1. I have trouble getting along with other teens	0	1	2	3	4
2. Other teens do not want to be my friend	0	1	2	3	4
3. Other teens tease me	0	1	2	3	4
4. I cannot do things that other teens my age can do	0	1	2	3	4
5. It is hard to keep up with my peers	0	1	2	3	4

**ABOUT SCHOOL *(problems with…)***	Never	Almost Never	Some-times	Often	Almost Always
1. It is hard to pay attention in class	0	1	2	3	4
2. I forget things	0	1	2	3	4
3. I have trouble keeping up with my schoolwork	0	1	2	3	4
4. I miss school because of not feeling well	0	1	2	3	4
5. I miss school to go to the doctor or hospital	0	1	2	3	4

Not to be reproduced without permission
Copyright © 1998 JW Varni, Ph.D. All rights reserved

ID# ____________________

Date: ____________________

PedsQL™

Young Adult Quality of Life Inventory

Version 4.0

YOUNG ADULT REPORT (ages **18-25**)

DIRECTIONS

On the following page is a list of things that might be a problem for you. Please tell us **how much of a problem** each one has been for you during the **past ONE month** by circling:

0 if it is **never** a problem
1 if it is **almost never** a problem
2 if it is **sometimes** a problem
3 if it is **often** a problem
4 if it is **almost always** a problem

There are no right or wrong answers.
If you do not understand a question, please ask for help.

Copyright © 1998 JW Varni, Ph.D. All rights reserved

*In the past **ONE month**, how much of a **problem** has this been for you…*

**ABOUT MY HEALTH AND ACTIVITIES *(problems with…)***	Never	Almost Never	Some-times	Often	Almost Always
1. It is hard for me to walk more than one block	0	1	2	3	4
2. It is hard for me to run	0	1	2	3	4
3. It is hard for me to do sports activity or exercise	0	1	2	3	4
4. It is hard for me to lift something heavy	0	1	2	3	4
5. It is hard for me to take a bath or shower by myself	0	1	2	3	4
6. It is hard for me to do chores around the house	0	1	2	3	4
7. I hurt or ache	0	1	2	3	4
8. I have low energy	0	1	2	3	4

**ABOUT MY FEELINGS *(problems with…)***	Never	Almost Never	Some-times	Often	Almost Always
1. I feel afraid or scared	0	1	2	3	4
2. I feel sad or blue	0	1	2	3	4
3. I feel angry	0	1	2	3	4
4. I have trouble sleeping	0	1	2	3	4
5. I worry about what will happen to me	0	1	2	3	4

**HOW I GET ALONG WITH OTHERS *(problems with…)***	Never	Almost Never	Some-times	Often	Almost Always
1. I have trouble getting along with other young adults	0	1	2	3	4
2. Other young adults do not want to be my friend	0	1	2	3	4
3. Other young adults tease me	0	1	2	3	4
4. I cannot do things that others my age can do	0	1	2	3	4
5. It is hard to keep up with my peers	0	1	2	3	4

**ABOUT MY WORK/STUDIES *(problems with…)***	Never	Almost Never	Some-times	Often	Almost Always
1. It is hard to pay attention at work or school	0	1	2	3	4
2. I forget things	0	1	2	3	4
3. I have trouble keeping up with my work or studies	0	1	2	3	4
4. I miss work or school because of not feeling well	0	1	2	3	4
5. I miss work or school to go to the doctor or hospital	0	1	2	3	4

Not to be reproduced without permission
Copyright © 1998 JW Varni, Ph.D. All rights reserved

ID#________________________

Date:________________________

PedsQL™

Family Impact Module

Version 2.0

PARENT REPORT

DIRECTIONS

Families of children sometimes have special concerns or difficulties because of the child's health. On the following page is a list of things that might be a problem for **you**. Please tell us **how much of a problem** each one has been for **you** during the **past ONE month** by circling:

0 if it is **never** a problem
1 if it is **almost never** a problem
2 if it is **sometimes** a problem
3 if it is **often** a problem
4 if it is **almost always** a problem

There are no right or wrong answers.
If you do not understand a question, please ask for help.

Not to be reproduced without permission

Copyright © 1998 JW Varni, PhD.

*In the past **ONE month**, as a result of your child's health, how much of a problem have **you** had with…*

**PHYSICAL FUNCTIONING** ***(problems with…)***	**Never**	**Almost Never**	**Some-times**	**Often**	**Almost Always**
1. I feel tired during the day	0	1	2	3	4
2. I feel tired when I wake up in the morning	0	1	2	3	4
3. I feel too tired to do the things I like to do	0	1	2	3	4
4. I get headaches	0	1	2	3	4
5. I feel physically weak	0	1	2	3	4
6. I feel sick to my stomach	0	1	2	3	4

**EMOTIONAL FUNCTIONING** ***(problems with…)***	**Never**	**Almost Never**	**Some-times**	**Often**	**Almost Always**
1. I feel anxious	0	1	2	3	4
2. I feel sad	0	1	2	3	4
3. I feel angry	0	1	2	3	4
4. I feel frustrated	0	1	2	3	4
5. I feel helpless or hopeless	0	1	2	3	4

**SOCIAL FUNCTIONING** ***(problems with…)***	**Never**	**Almost Never**	**Some-times**	**Often**	**Almost Always**
1. I feel isolated from others	0	1	2	3	4
2. I have trouble getting support from others	0	1	2	3	4
3. It is hard to find time for social activities	0	1	2	3	4
4. I do not have enough energy for social activities	0	1	2	3	4

**COGNITIVE FUNCTIONING** ***(problems with…)***	**Never**	**Almost Never**	**Some-times**	**Often**	**Almost Always**
1. It is hard for me to keep my attention on things	0	1	2	3	4
2. It is hard for me to remember what people tell me	0	1	2	3	4
3. It is hard for me to remember what I just heard	0	1	2	3	4
4. It is hard for me to think quickly	0	1	2	3	4
5. I have trouble remembering what I was just thinking	0	1	2	3	4

**COMMUNICATION** ***(problems with…)***	**Never**	**Almost Never**	**Some-times**	**Often**	**Almost Always**
1. I feel that others do not understand my family's situation	0	1	2	3	4
2. It is hard for me to talk about my child's health with others	0	1	2	3	4
3. It is hard for me to tell doctors and nurses how I feel	0	1	2	3	4

Not to be reproduced without permission
Copyright © 1998 JW Varni, PhD.

*In the past **ONE month**, as a result of your child's health, how much of a problem have **you** had with…*

**WORRY** ***(problems with…)***	**Never**	**Almost Never**	**Some-times**	**Often**	**Almost Always**
1. I worry about whether or not my child's medical treatments are working	0	1	2	3	4
2. I worry about the side effects of my child's medications/medical treatments	0	1	2	3	4
3. I worry about how others will react to my child's condition	0	1	2	3	4
4. I worry about how my child's illness is affecting other family members	0	1	2	3	4
5. I worry about my child's future	0	1	2	3	4

DIRECTIONS

Below is a list of things that might be a problem for **your family**. Please tell us **how much of a problem** each one has been for **your family** during the **past ONE month.**

*In the past **ONE month**, as a result of your child's health, how much of a problem has **your family** had with…*

**DAILY ACTIVITIES** ***(problems with…)***	**Never**	**Almost Never**	**Some-times**	**Often**	**Almost Always**
1. Family activities taking more time and effort	0	1	2	3	4
2. Difficulty finding time to finish household tasks	0	1	2	3	4
3. Feeling too tired to finish household tasks	0	1	2	3	4

**FAMILY RELATIONSHIPS** ***(problems with…)***	**Never**	**Almost Never**	**Some-times**	**Often**	**Almost Always**
1. Lack of communication between family members	0	1	2	3	4
2. Conflicts between family members	0	1	2	3	4
3. Difficulty making decisions together as a family	0	1	2	3	4
4. Difficulty solving family problems together	0	1	2	3	4
5. Stress or tension between family members	0	1	2	3	4

Not to be reproduced without permission
Copyright © 1998 JW Varni, PhD.

Appendix 5 Guideline for Fat-Soluble Vitamin Supplementation

Appendix 5: Guideline for Fat-Soluble Vitamin Supplementation

Cholestasis predisposes to fat-soluble vitamin deficiencies. Fat-soluble vitamin levels, i.e., vitamins A and E, 25-hydroxy vitamin D, and INR (surrogate for vitamin K) are measured routinely during the study. If a patient has any fat-soluble vitamin level(s) that are out of range, vitamin supplementation adjustments may be required. Below are suggested guidelines for fat-soluble vitamin deficiency treatment [Venkat 2014; Shneider 2012]. Additional patient monitoring and/or treatment strategies may be warranted at the discretion of the investigator.

Target Fat-soluble Vitamin Levels and Replacement Regimens

Vitamin	Target Range (Serum Level)	Supplementation Strategy
A (retinol)	19 to 77 µg/dL* retinol:retinol-binding protein molar ratio > 0.8	Increments of 5000 IU (up to 25,000 to 50,000 IU/day) orally or monthly intramuscular administration of 50,000 IU**
D (25-hydroxy vitamin D)	15 to 45 ng/mL*	Increments of 1200 to 8000 IU orally daily of cholecalciferol or ergocalciferol; alternatively calcitriol at 0.05 to 0.2 µg/kg/day*
E (α tocopherol)	3.8 to 20.3 µg/mL vitamin E:total serum lipids ratio >0.6 mg/g	Increments of 25 IU/kg of d-α-tocopheryl polyethylene glycol – 1000 succinate (TPGS) orally daily (to 100 IU/kg/day)
K (phytonadione)	INR ≤1.2	• 1.2 <INR ≤1.5: 2.5 mg vitamin K orally daily<br>• 1.5 <INR ≤1.8: 2.0 to 5.0 mg vitamin K intramuscular and 2.5 mg vitamin K orally daily<br>• INR >1.8: 2.0 to 5.0 mg vitamin K intramuscular and 5.0 mg vitamin K orally daily

***Clinical practice may vary. <u>This is meant as a guidance only and does not override local standard of care. Investigators should provide best case practices for management and treatment</u>**

**** For further detail regarding age related Tolerable Upper Intake (UL) for preformed vitamin A please refer to page 163 in the European Food Safety Authority guidance: *Tolerable Upper Intake Levels for Vitamins and Minerals*; Scientific Committee on food Scientific Panel on Dietetic Products, Nutrition and Allergies**
http://www.efsa.europa.eu/sites/default/files/efsa_rep/blobserver_assets/ndatolerableuil.pdf

Appendix 6 Contraceptive Requirements

Appendix 6: Contraceptive Requirements

A woman is considered of childbearing potential (WOCBP), i.e. fertile, following menarche and until becoming post-menopausal unless permanently sterile. Permanent sterilisation methods include hysterectomy, bilateral salpingectomy and bilateral oophorectomy.

Contraceptive methods, or combinations of contraceptive methods, for males and females that can achieve a failure rate of less than 1% per year when used consistently and correctly are considered as highly effective birth control methods and must be used at least to up to 90 days following the last day of treatment.

Such methods include:

- Combined (estrogen and progestogen containing) hormonal contraception associated with inhibition of ovulation:
    - oral
    - intravaginal
    - transdermal
- Progestogen-only hormonal contraception associated with inhibition of ovulation:
    - oral
    - injectable
    - implantable
- Intrauterine device (IUD)
- Intrauterine hormone-releasing system (IUS)
- Bilateral tubal occlusion
- Vasectomised partner
- Sexual abstinence

Sexual abstinence is considered a highly effective method only if defined as refraining from heterosexual intercourse during the entire study period up to at least 90 days after the last day of treatment. The reliability of sexual abstinence needs to be evaluated in relation to the duration of the clinical trial and the preferred and usual lifestyle of the

subject. Periodic abstinence (e.g., calendar, ovulation, symptothermal, post-ovulation methods) and withdrawal are not acceptable methods of contraception.

Requirements according to "Recommendations related to contraception and pregnancy testing in clinical trials", HMA CTFG (Clinical Trial Facilitation Group), 2014

Appendix 7 Blood Volumes

[redacted]

Scientific Affairs
Estimated Blood Volume Request

Version: 15.0

SPONSOR: Albireo **Protocol#: A4250-008** **Age Group 0 months to 30 years of age**

Visit^	Testing^1-9	Tube volume (mL)	Tube Type	# of Collection Tubes	# of Aliquots	Volume (mLs)	Volume/ visit (mL)	Volume/ visit (tsp)	Volume/ visit (TBS)
Visit S-1	Chemistry, hCG	1 1	SST	1	1	1 1			
Screening Period	Bile Acid[1]	1 1	SST	1	1	1 1			
Cohort 2 Only	DNA[7]	3 0	EDTA	1	0	3 0			
							5 2	1.05	0.35
Visit S-2	Coagulation (INR/PT)	1 4	NaCit	1	1	1 4			
Screening Period	Bile Acid[1]	1 1	SST	1	1	1 1			
Cohort 2 Only									
							2 5	0.51	0.17
Visit 1	Chemistry[3], AFP, hCG (hCG Cohort 1 only)	1 1	SST	1	1	1 1			
same as Visit 9/EOT in A4250-005	Hematology	1 0	EDTA	1	0	1 0			
	Bile Acid[1]	1 1	SST	1	1	1 1			
	C4[2]	1 2	LiHep	1	2	1 2			
	Autotaxin[5]	1 2	LiHep	1	2	1 2			
	Vitamins, A, E[1]	2 5	SST	1	1	2 5			
	Vitamin D,25OH	1 1	SST	1	1	1 1			
						0 0			
							9 2	1.87	0.62
Visit 2	Chemistry	1 1	SST	1	1	1 1			
4 weeks	Hematology	1 0	EDTA	1	0	1 0			
	Coagulation (INR/PT)	1 4	NaCit	1	1	1 4			
	Bile Acid[1]	1 1	SST	1	1	1 1			
	Autotaxin[5]	1 2	LiHep	1	2	1 2			
	C4[2]	1 2	LiHep	1	2	1 2			
						0 0			
							7 0	1.42	0.47
Visit 3	Chemistry	1 1	SST	1	1	1 1			
12 weeks	Hematology	1 0	EDTA	1	0	1 0			
	Coagulation (INR/PT)	1 4	NaCit	1	1	1 4			
	Bile Acid[1]	1 1	SST	1	1	1 1			
	Vitamins, A, E[1]	2 5	SST	1	1	2 5			
	Vitamin D,25OH	1 1	SST	1	1	1 1			
						0 0			
							8 2	1.66	0.55
Visit 4	Coagulation (INR/PT)	1 4	NaCit	1	1	1 4			
22 weeks	Bile Acid[1]	1 1	SST	1	1	1 1			
	Vitamins, A, E[1]	2 5	SST	1	1	2 5			
	Vitamin D,25OH	1 1	SST	1	1	1 1			
						0 0			
							6 1	1.24	0.41
Visit 5	Chemistry, AFP	1 1	SST	1	1	1 1			
24 weeks	Hematology	1 0	EDTA	1	0	1 0			
	Bile Acid[1]	1 1	SST	1	1	1 1			
	C4[2]	1 2	LiHep	1	2	1 2			
	Autotaxin[5]	1 2	LiHep	1	2	1 2			
						0 0			
							5 6	1.14	0.38
Visit 6	Chemistry	1 1	SST	1	1	1 1			
36 weeks	Hematology	1 0	EDTA	1	0	1 0			
	Coagulation (INR/PT)	1 4	NaCit	1	1	1 4			
	Bile Acid[1]	1 1	SST	1	1	1 1			
	Vitamins, A, E[1]	2 5	SST	1	1	2 5			
	Vitamin D,25OH	1 1	SST	1	1	1 1			
						0 0			
							8 2	1.66	0.55
Visit 7	Coagulation (INR/PT)	1 4	NaCit	1	1	1 4			
46 weeks	Bile Acid[1]	1 1	SST	1	1	1 1			
	Vitamins, A, E[1]	2 5	SST	1	1	2 5			

Scientific Affairs
Estimated Blood Volume Request

Version: 15.0

SPONSOR: Albireo **Protocol#: A4250-008** **Age Group 0 months to 30 years of age**

Visit^	Testing^[1-9]	Tube volume (mL)	Tube Type	# of Collection Tubes	# of Aliquots	Volume (mLs)	Volume/ visit (mL)	Volume/ visit (tsp)	Volume/ visit (TBS)
	Vitamin D,25OH	1 1	SST	1	1	1 1			
						0 0			
							6 1	1.24	0.41
Visit 8	Chemistry, AFP	1 1	SST	1	1	1 1			
48 weeks	Hematology	1 0	EDTA	1	0	1 0			
	Bile Acid[1]	1 1	SST	1	1	1 1			
	C4[2]	1 2	LiHep	1	2	1 2			
	Autotaxin[5]	1 2	LiHep	1	2	1 2			
						0 0			
							5 6	1.14	0.38
Visit 9	Chemistry	1 1	SST	1	1	1 1			
60 weeks	Hematology	1 0	EDTA	1	0	1 0			
	Coagulation (INR/PT)	1 4	NaCit	1	1	1 4			
	Bile Acid[1]	1 1	SST	1	1	1 1			
	Vitamins, A, E[1]	2 5	SST	1	1	2 5			
	Vitamin D,25OH	1 1	SST	1	1	1 1			
						0 0			
							8 2	1.66	0.55
Visit 10	Coagulation (INR/PT)	1 4	NaCit	1	1	1 4			
70 weeks	Bile Acid[1]	1 1	SST	1	1	1 1			
	Vitamins, A, E[1]	2 5	SST	1	1	2 5			
	Vitamin D,25OH	1 1	SST	1	1	1 1			
						0 0			
							6 1	1.24	0.41
Visit 11/EOT	Chemistry, AFP	1 1	SST	1	1	1 1			
72 weeks	Hematology	1 0	EDTA	1	0	1 0			
	Bile Acid[1]	1 1	SST	1	1	1 1			
	C4[2]	1 2	LiHep	1	2	1 2			
	Autotaxin[5]	1 2	LiHep	1	2	1 2			
						0 0			
							5 6	1.14	0.38
Visit 12	Chemistry	1 1	SST	1	1	1 1			
Follow up	Hematology	1 0	EDTA	1	0	1 0			
76 Weeks	Coagulation (INR/PT)	1 4	NaCit	1	1	1 4			
	Bile Acid[1]	1 1	SST	1	1	1 1			
						0 0			
							4 6	0.93	0.31
						0 0			
							0 0	0.00	0.00
**Study Total Volume***							80.5	16.3	5.4
**Study Total Volume Cohort 2***							88.2	17.9	6.0
**Extension Period (Every 16 Weeks/ Extension Period EOT)***									
	Chemistry, AFP	1 1	SST	1	1	1 1			
	Bile Acid[1]	1 1	SST	1	1	1 1			
	Vitamins, A, E[1]	2 5	SST	1	1	2 5			
	Vitamin D,25OH	1 1	SST	1	1	1 1			
	INR	1 4	NaCit	1	1	1 4			
						0 0			
**Extension Period Maximun Volume per Visit[8]**							7 2	1.46	0.49

^ Visit Schedule and testing based on protocol dated Protocol amendment 01 dated 06-Apr-2018 Added Optional DNA to Visit S-1 and Vitamin testing to Visits 6 and 9 per sponsor notification of inclusion in an updated Protocol Amendment dated 18-Jan-2019 *Note, Visit S-1 and Visit S-2 were added as part of the protocol amendment

*Total Volume reflects only the volumes required for testing by ICL and does not include any possible testing performed by 3rd party Laboratories

The blood volume for visit 1 is included although collected during A4250-005 study (Cohort 1 only)

Notes: #1 Bile Acids, Total, Vitamin A, and Vitamin E testing at ARUP Lab, USA

#2 C4 - will be performed on plasma at LGC

#3 The following 'minimum' volume does not allow for repeat testing

Chemistry, AFP requires minimum – 500uL serum

Bile Acids requires minimum – 500uL serum

Coagulation (PT/INR only) requires minimum – 900uL plasma

#4 Sites must collect Full Draw and measure exact aliquot of sample into a "False Bottom" transport tube

[redacted]

Scientific Affairs
Estimated Blood Volume Request
Version: 15.0

SPONSOR: Albireo **Protocol#: A4250-008** **Age Group 0 months to 30 years of age**

Visit^	Testing^[1-9]	Tube volume (mL)	Tube Type	# of Collection Tubes	# of Aliquots	Volume (mLs)	Volume/ visit (mL)	Volume/ visit (tsp)	Volume/ visit (TBS)

#5 Autotaxin serum samples will be tested at LGC
#6 For subjects requiring optional pregnancy testing, collect 1 1 mL SST
#7 Optional Sample Only collected if required
#8 Collection requirements for Extension Period Visit added per provision of schedule of assessments provided by sponsor for utiliztion in an upcoming revised protocol
#9 For patients who experience a hepatic adverse event and/or hepatic decompensation, collect 1 1 mL Plasma for PK

Created by: Jennifer Rodriguez, Protocol Review
Date: 19-Mar-2020
