# Supplementary material for: Interim results from an ongoing, open-label, single-arm trial of odevixibat in progressive familial intrahepatic cholestasis
Source: JHEP Rep. 2023 Apr 29;5(8):100782. doi: 10.1016/j.jhepr.2023.100782 (PMC10338319; doi:10.1016/j.jhepr.2023.100782)
Supplement: Multimedia component 4 [file mmc4.zip › Clinical Trial/A4250_008_SAP_Final v1.2_Redacted.pdf]

Statistical Analysis Plan (SAP)

**Protocol title:**	An Open-Label Extension Study to Evaluate Long-Term Efficacy and Safety of A4250 in Children with Progressive Familial Intrahepatic Cholestasis Types 1 and 2 (PEDFIC 2)
**Protocol no.:**	A4250-008
**IND no:**	130591
**EudraCT no.:**	2017-002325-38
**Protocol version no., date:**	Amendment 04, 25 Oct 2019
**Document version no., date:**	Final v1.2, 25 Aug 2020
**Prepared by:**	[redacted]

Signature Page

Prepared by:

[redacted]

Signature	Date

Reviewed by:

[redacted]

Signature	Date

Reviewed and approved by:

[redacted]

Signature	Date

[redacted]

Signature	Date

Revision History

VERSION/DATE	MODIFIED BY	REASON FOR MODIFICATIONS
Final v1.0/20Mar2020	NA	
Final v1.1/11Aug2020	[redacted]	1. To clarify missing data will not be imputed for analysis.<br>2. To update dictionary version.<br>3. To clarify the growth analysis will be based on derived growth data using the software or methods from the Centers for Disease Control and Prevention (CDC) website for patients with age ≥ 2 years old and from the WHO website for patients with age < 2 years old.<br>4. To clarify the counting of number of adverse events.<br>5. To clarify that local laboratory data will be used when central laboratory data are not available.<br>6. To clarify the calculation of change from baseline for AM&PM pruritus scores (daily, weekly, bi-weekly, monthly) for analysis purposes.<br>7. To update the analysis windows of non-eDiary assessments.<br>8. To add the analysis windows for the endpoints of eDiary assessments.
Final v1.2/25Aug2020	[redacted]	1. To clarify the baseline derivation of serum bile acid

Table of Contents

Signature Page 2
Revision History 3
List of Appendices 6
List of Tables 6
List of Figures 7
List of Abbreviations 8
1. Introduction 11
2. Study Objectives 11
2.1 Primary Objectives 11
2.2 Secondary Objectives 12
3. Study Design 12
3.1 General Study Design 12
3.2 Randomisation and Blinding 16
3.3 Study Treatments and Assessments 16
3.3.1 Serum Bile Acids 17
3.3.2 Itching, Scratching, and Sleep Score 17
3.3.3 Growth 18
3.3.4 Biomarker Sample 18
3.3.5 Change of Antipruritic Medication 18
3.3.6 Quality of Life Questionnaire (PedsQL) 19
3.3.7 PELD/MELD Score 19
3.3.8 Fibroscan® 20
3.3.9 Markers of Fibrosis 20
3.3.10 Liver Biopsy 21
3.3.11 Global Impression of Change and Global Impression of Symptom Measures 21
4. Study Endpoints 28
4.1 Primary Efficacy Endpoints 28
4.2 Secondary Efficacy Endpoints 29

4.3 Exploratory Endpoints 30
4.4 Safety Evaluation 31
5. Sample Size and Power 32
6. Interim Analysis 33
7. Analysis Sets 33
8. Statistical Considerations and Analysis 34
8.1 Handling of Missing Data and/or Invalid Data and Outliers 34
8.1.1 Missing Data Analysis Methods for Efficacy Endpoints 34
9. Statistical Methods 34
9.1 General Statistical Conventions 34
9.2 Patient Disposition 35
9.3 Protocol Deviations 36
9.4 Demographic and Baseline Characteristics 37
9.4.1 Demographic Characteristics 37
9.4.2 Baseline and Disease Characteristics 37
9.4.3 Medical and Surgical History 38
9.4.4 Prior and Concomitant Medications 38
9.5 Extent of Exposure 39
9.5.1 Treatment Duration 39
9.5.2 Treatment Compliance 39
9.6 Efficacy Analyses 40
9.6.1 Analysis Methods 40
9.6.2 Treatment by Centre Interaction Analysis (Multicentre Study) 41
9.6.3 Analyses of Primary Efficacy Endpoints 41
9.6.4 Analyses of Secondary Endpoints 42
9.6.5 Analyses of Exploratory Endpoints 43
9.7 Safety Analyses 45
9.7.1 Adverse Events 45
9.7.2 Clinical Laboratory Evaluations 51
9.7.3 Vital Sign Measurements 53

9.7.4 Physical Examinations 53
9.8 Other Analyses 54
9.8.1 Subgroup Analyses 54
9.8.2 Data Safety Monitoring Board 54
10. Changes to Planned Analysis from Study Protocol 54
11. Appendices 56

List of Appendices

Appendix A: Derived Variables 56
Appendix B: Visit Window 63
Appendix C: Definition of Region Variable 67
Appendix D: Handling of Missing or Incomplete Dates 68
Appendix E: Normal Reference Ranges of Vital Signs 70

List of Tables

Table 1: Fibroscan Scoring for Cholestatic Liver Disease (Fibrosis Score) 20
Table 2: Controlled Attenuation Parameter Score and Steatosis Grading 20
Table 3: Schedule of Study Assessments 22
Table 4: Laboratory Parameters 52
Table 5: Derived Variables for Demographic and Baseline Characteristics, Various Duration Derivations, Drug Compliance, Baseline Derivations, and Other Important Derivations 57
Table 6: Analysis Visit Window (General) 63
Table 7: Analysis Visit Windows for GIC/GIS, Physical Measurements and Selected Lab Test) 64
Table 8: Analysis Visit Window for PedsQL, Fibroscan, Abdominal Ultrasound and AFP 65
Table 9: Definition of Region Variable 67
Table 10: Heart Rate by Age (Beats/Minute) Reference 70
Table 11: Normal Respiratory Rate by Age (Breaths/Minute) Reference 70
Table 12: Normal Blood Pressure by Age (mm Hg) Reference 70
Table 13: Normal Temperature Range by Method 71

List of Figures

Figure 1: Study Design 14

List of Abbreviations

Abbreviation	Definition
AE(s)	Adverse Event(s)
AFP	Alfa-fetoprotein
ALP	Alkaline Phosphatase
ALT	Alanine Aminotransferase
APRI	Aspartate Aminotransferase to Platelet Ratio Index
AST	Aspartate Aminotransferase
ATC	Anatomical Therapeutic Chemical
BMI	Body Mass Index
CAP	Controlled Attenuation Parameter
CDC	Centers for Disease Control and Prevention
CDF	Cumulative Distribution Function
CI	Confidence Interval
COVID-19	Coronavirus Disease 2019
CPK	Creatine Phosphokinase
CSR	Clinical Study Report
DSMB	Data Safety and Monitoring Board
eCRF	Electronic Case Report Form
eDiary	Electronic Diary
EOT	End of Treatment
EU	European Union
FAS	Full Analysis Set
Fib-4	Fibrosis-4
GGT	Gamma-glutamyl Transferase
GIC	Global Impression of Change
GIS	Global Impression of Symptoms
ICF	Informed Consent Form

(Continued on next page)

Abbreviation	Definition
ICH	International Council for Harmonisation
INR	international normalised ratio
ISE	Integrated Summary of Effectiveness
ISS	Integrated Summary of Safety
LDH	Lactate Dehydrogenase
MAA	Marketing Authorisation Application
MedDRA	Medical Dictionary for Regulatory Activities
MELD	Model for End-stage Liver Disease
NA	Not Applicable
NAPPED	NAtural Course and Prognosis of PFIC and Effect of biliary Diversion
NDA	New Drug Application
ObsRO	Observer-reported Outcome
p-C4	Plasma 7α-hydroxy-4-cholesten-3-one Concentration
PedsQL	Pediatric Quality of Life Inventory
PELD	Paediatric End-stage Liver Disease
PFIC	Progressive Familial Intrahepatic Cholestasis
PRO	Patient-reported Outcome
PT	Preferred Term
QoL	Quality of Life
RoW	Rest of World
SAE(s)	Serious Adverse Event(s)
SAP	Statistical Analysis Plan
SAS®	Statistical Analysis System Software
SE	Standard Error
SI	International System of Unit
SOC	System Organ Class
StdDev	Standard Deviation

(Continued on next page)

Abbreviation	Definition
T	Telephone Contact
TEAE(s)	Treatment-emergent Adverse Event(s)
TFLs	Tables, Figures, and Listings
ULN	Upper Limit of Normal
US	United States
V	Clinic Visit
VS	Visit Screening
WHO	World Health Organization

1. Introduction

The purpose of this statistical analysis plan (SAP) is to provide detailed descriptions of the statistical methods, data derivations, and data displays for Study Protocol A4250-008, *An Open-Label Extension Study to Evaluate Long-Term Efficacy and Safety of A4250 in Children with Progressive Familial Intrahepatic Cholestasis Types 1 and 2 (PEDFIC 2)*. The table of contents and templates for the tables, figures, and listings (TFLs) will be produced in a separate document.

Any deviations from this SAP will be described and justified in the clinical study report (CSR). The preparation of this SAP has been based on International Council for Harmonisation (ICH) E3 and E9 guidelines[1],[2]. All data analyses and generation of TFLs will be performed using Statistical Analysis System (SAS®) software (Version 9.4 or higher). The SAP will be finalised and signed off prior to locking the database.

2. Study Objectives

2.1 Primary Objectives

Cohort 1

To demonstrate a sustained effect of odevixibat (A4250) on serum bile acids and pruritus in children with progressive familial intrahepatic cholestasis (PFIC) Types 1 and 2.

Cohort 2

To evaluate the effect of odevixibat on serum bile acids and pruritus in patients with PFIC who either (1) Do not meet eligibility criteria for Study A4250-005 (PEDFIC 1) or (2) Patients who do meet the eligibility criteria for Study A4250-005 after recruitment of Study A4250-005 has been completed.

---

[1] ICH Topic E3: Structure and Content of Clinical Study Reports (CPMP/ICH/137/95- adopted December 1995)

[2] ICH Topic E9: Statistical Principles for Clinical Trials (CPMP/ICH/363/96 – adopted March 1998).

2.2 Secondary Objectives

Cohorts 1 and 2:

- To evaluate the long-term safety and tolerability of repeated daily doses of odevixibat
- To evaluate the effect of odevixibat on growth
- To evaluate the effect of odevixibat on biliary diversion and/or liver transplantation
- To evaluate the effect of odevixibat on biochemical markers of cholestasis and liver disease

3. Study Design

3.1 General Study Design

This is a Phase 3, multicentre, open-label extension study to investigate the long-term efficacy and safety of a 120 µg/kg/day daily dose of odevixibat in patients with PFIC. Cohort 1 will consist of children with PFIC Types 1 and 2 who have participated in Study A4250-005. Cohort 2 will consist of approximately 60 patients with any type of PFIC who have elevated serum bile acids and cholestatic pruritus and who either (1) Do not meet eligibility criteria for Study A4250-005 (PEDFIC 1) or (2) Are eligible for enrolment in Study A4250-005 after recruitment of Study A4250-005 has been completed. Up to 40 patients post-biliary diversion surgery are allowed to participate in Cohort 2.

Study data will be reviewed periodically (approximately on a quarterly basis) by a Data Safety and Monitoring Board (DSMB) until the last patient reaches 72 weeks.

Informed consent must be obtained before any study procedures are performed. After signing the informed consent form (ICF), patients will be evaluated for study eligibility and considered to be enrolled in the study. For Cohort 1, patients are eligible who completed 24 weeks of treatment in Study A4250-005, or who withdrew from Study A4250-005 after a minimum of 12 weeks of treatment with odevixibat due to intolerable symptoms but met all inclusion criteria and no exclusion criteria. Patients who have

withdrawn from Study A4250-005 due to a study drug-related adverse event (AE) will not be eligible. For Cohort 2, patients meeting all inclusion criteria and no exclusion criteria are eligible.

Eligible patients will be enrolled into this open-label extension study and treated with a daily dose of 120 µg/kg/day of odevixibat for 72 weeks. Patients who wish to continue receiving odevixibat after 72 weeks may have the option to remain on treatment in an extension period with visits every 16 weeks until the drug is commercially available, provided continued use is supported by the risk-benefit profile and the patient has not been previously withdrawn or discontinued from the study. In that case the 4-week Follow-Up period will not occur.

Patients in Cohort 2 will go through a Screening period consisting of 2 clinic visits as follows:

- Visit S-1: Screening Visit 1 (Days -56 to -35)
- Visit S-2: Screening Visit 2 (Days -28 to -7)

Patients who do not meet eligibility criteria may be re-screened after consultation with the Medical Monitor. Patients not fulfilling inclusion/exclusion criteria after 3 attempts may not be re-screened.

All patients will have a minimum of 12 clinic visits and 6 scheduled telephone contacts (see Figure 1) as follows:

Visit 1: Screening/inclusion visit (Day 1; coincides with Visit 9/End of Treatment (EOT) in Study A4250-005 for patients in Cohort 1. Assessments that have been performed at Visit 9/EOT during Study A4250-005 will not be repeated.)

- Visit 2: Week 4
  − Telephone contact 1: Week 8
- Visit 3: Week 12
  − Telephone contact 2: Week 18
- Visit 4: Week 22
- Visit 5: Week 24
  − Telephone contact 3: Week 30

- Visit 6: Week 36
  − Telephone contact 4: Week 42
- Visit 7: Week 46
- Visit 8: Week 48
  − Telephone contact 5: Week 54
- Visit 9: Week 60
  − Telephone contact 6: Week 66
- Visit 10: Week 70
- Visit 11: Week 72/optional extension period
- Visit 12: Follow-Up visit; Week 76 (for those not participating in optional extension period)
- Optional extension period visits every 16 weeks; Week 88 and onward

Figure 1: Study Design

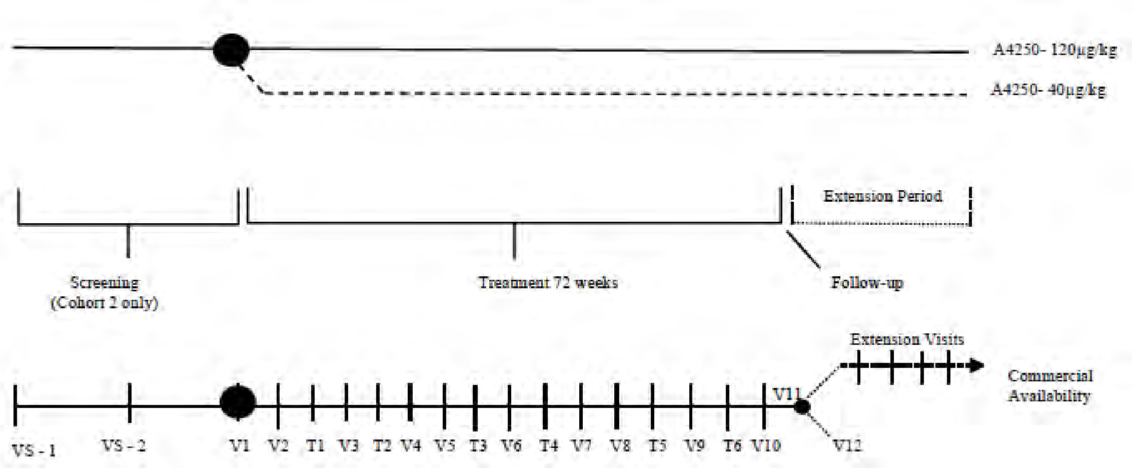

T: telephone contact; V: clinic visit; VS: visit screening

Patients will return to the clinic at Weeks 4 and 12 and thereafter every 10 to 12 weeks for follow-up measurements. Between clinic visits there will be a telephone contact with the patient and/or caregiver to report AEs. Additional clinic visits may be required for patients who need direct site assistance for AE monitoring for safety maintenance and other assessments.

At Visit 11, patients will be offered the choice to continue receiving odevixibat treatment in an optional extension period. During the extension period the patient will receive 120 µg/kg/day odevixibat, returning to the clinic every 16 weeks until the drug is commercially available, provided continued use is supported by the risk-benefit profile and the patient has not been previously withdrawn or discontinued from study.

If a patient is prematurely withdrawn from the study, all assessments scheduled for Visit 11 will be performed at the time the patient withdraws. If a patient participating in the optional extension study withdraws prior to commercial drug availability, all assessments scheduled for the optional extension period EOT visit will be performed.

If a liver biopsy is performed according to the local regulations or standard of care at any time during A4250-008, the biopsy results should be recorded in the electronic case report form (eCRF). If liver biopsy results were collected for a patient in Study A4250-005 or prior to Week 24 in Study A4250-008, the patient will be asked to consent to a follow-up liver biopsy at Visit 11, if allowed per local regulations, unless 2 prior liver biopsy results have been collected (with at least 1 year between biopsies). Liver biopsies will not be performed in Spain.

For patients not participating in the optional extension period, Visit 12 will take place 28 days after Visit 11. All patients who are prematurely withdrawn will have this visit 28 days after the last dose of study drug for AE monitoring and other follow-up assessments.

If a patient discontinues before Week 72, additional telephone contacts will be made every 3 months after the Follow-Up visit (Visit 12) up to Week 72 to inquire whether the patient has undergone biliary diversion or liver transplantation.

Patients and/or caregivers will be instructed to complete the electronic diary (eDiary) every morning and evening for the first 24 weeks (Visits 1 to 5) and for the 21 days before each clinic visit thereafter (Visits 6 to 12). In addition, Cohort 2 patients and/or caregivers will be instructed to complete the eDiary during the Screening period. Patients participating in the optional extension period will not complete eDiary entries after Visit 11. The eDiary will include patient-reported outcome (PRO) and observer-reported outcome (ObsRO) items for evaluation of itching (using PRO), scratching (using ObsRO), and sleep disturbance (using both PRO and ObsRO). ObsRO in patients of all

ages will be recorded by a caregiver. If possible, the same caregiver will complete the ObsRO items throughout the study. Additionally, caregivers will be requested to report in the diary the time that study drug was administered during the treatment period.

The study nurse will monitor eDiary compliance by routine review of the CRF Health website. If both diary entries are missing on a specific day during this time, the study nurse will call the caregiver/patient with a reminder to complete all scheduled entries. Non-compliance will be documented and explained in the source documents.

3.2 Randomisation and Blinding

Not applicable (NA)

3.3 Study Treatments and Assessments

Patients will be dosed with 120 µg/kg/day for 72 weeks. Patients not tolerating the dose after a minimum of 1 week for reasons other than new liver findings and severe diarrhoea will have the option to down-titrate to a lower dose (40 µg/kg/day). They should return to the higher dose as soon as deemed appropriate by the Investigator. More than one upward dose titration (from 40 µg/kg/day directly to 120 µg/kg/day) for the same event is not recommended. If, in the opinion of the Investigator, a dose titration should be considered prior to the 1-week minimum, the Investigator should consult with the Medical Monitor or designee. Any dose titration should be done in consultation with the Sponsor Medical Monitor or designee.

Study drug will be dispensed to the patient at defined intervals from Visit 1 through Visit 9, together with instructions on how to store and take the drug. Study drug administration data, including whether each patient took each dose or partial doses of study drug, whether there were any delayed or missed doses, and whether the capsule was opened or swallowed whole, will be documented through the diaries and transferred to the study database.

Patients participating in the optional extension period will be dosed with 120 µg/kg/day. Patients not tolerating the dose at any time throughout the extension period will have the option to down-titrate to a lower dose (40 µg/kg/day) following consultation with the Sponsor Medical Monitor or designee. Patients should return to the higher dose as soon

as deemed appropriate by the Investigator. More than one upward dose titration (from 40 µg/kg/day directly to 120 µg/kg/day) for the same event is not recommended.

Bottles with 34 capsules will be given to the patient at each visit. A patient who requires 2 or more capsules per day will be given multiple bottles; please refer to the investigational product manual. If a patient's weight changes at any time during the study, dose adjustment will be required. The number of capsules provided to the patient should be based on the body weight thresholds identified in Protocol A4250-005, Table 2.

Odevixibat should be taken in the morning together with food. On clinic visits days when laboratory assessments are conducted (Visits 1 to 10; or Visits 1 to [XX] for those participating in the optional extension period), study drug should be taken after the visit.

Efficacy assessments and daily recording of pruritus using an eDiary in this study are briefly introduced as follows.

3.3.1 Serum Bile Acids

Blood samples for analysis of fasting total serum bile acids will be drawn at all visits from Visit 1 (Visit S-1 for Cohort 2) through Visit 12. Fasting serum bile acids will also be drawn at all visits during the optional extension period. Patients will fast (water intake only) for at least 4 hours prior to the collection of samples for serum bile acids. Exceptions can be made for infants <12 months of age if unable to fast for the full 4 hours. For any visit at which a bile acid sample result is unreportable, an additional unscheduled visit for a repeat sample collection may be scheduled. Samples will be handled and transported to a central laboratory per instructions in the laboratory manual.

3.3.2 Itching, Scratching, and Sleep Score

Itching, observed scratching, and sleep disturbance will be recorded twice each day via the eDiary. Patient and/or caregivers will be instructed to fill in the eDiary every morning and evening for the first 24 weeks and the last 21 days before all the remaining visits to the clinic. There will be no interruption between Visits 11 and 12. Patients participating in the optional extension period will stop eDiary entries at Visit 11. Patients and/or

caregivers will be instructed to complete the eDiary in the morning after the patient awakens and in the evening just before the patient goes to sleep.

The eDiary includes Albireo ObsRO and PRO items. Patients <8 years of age will not be asked to complete the Albireo PRO items; the Albireo ObsRO will be completed by caregivers of patients in this age group. Older patients, 8 to 18 years of age, will complete the Albireo PRO items and the caregiver will complete the Albireo ObsRO items. The Albireo PRO items assess severity of itch, aspects of sleep disturbance (morning diary only), and tiredness. For patients 8 to 12 years of age, the caregiver will read the Albireo PRO items along with the child and record the child's response. A guide will be provided to the caregivers that provides standardised explanations of the Albireo PRO items, in case the patient is confused or requires clarification about the meaning of a question. The Albireo ObsRO items assess the severity of observed scratching, the aspects of observed sleep disturbance (morning diary only), and the observed signs of tiredness (evening diary only). The Albireo ObsRO and PRO scratching and itch severity items use 0 to 4 response scales, where each response is distinguished by a unique facial expression, verbal anchor, number, and colour code.

3.3.3 Growth

Growth will be measured as height (velocity) and weight (Z-score) using a certified weight scale. Body mass index (BMI) will be calculated by weight (kg)/height (m^2). Change will be defined as linear growth deficit (weight and BMI for age) compared to standard growth curve (Z-score, standard deviation [StdDev] from the fiftieth percentile [StdDev from P50]).

3.3.4 Biomarker Sample

Blood samples for plasma 7α-hydroxy-4-cholesten-3-one concentration (p-C4) and autotaxin will be drawn at Visits 1, 2, 5, 8, and 11 (p-C4 and autotaxin will only be drawn for patients with body weight >10 kg). Samples will be treated and transported to a central laboratory per instructions in the laboratory manual.

3.3.5 Change of Antipruritic Medication

Any change of antipruritic medication must be noted in the eCRF.

3.3.6 Quality of Life Questionnaire (PedsQL)

Patients and caregivers will be asked to complete a quality of life (QoL) questionnaire, the Pediatric Quality of Life Inventory (PedsQL), at Visits 1, 5, 8, 11 and at all visits of the optional extension period. Details of the questions included on the questionnaire are located in the protocol.

3.3.7 PELD/MELD Score

The paediatric end-stage liver disease (PELD) score will be calculated for children under 12 years of age. For children 12 years of age or older, the model for end-stage liver disease (MELD) score will be calculated.

For calculation of the PELD/MELD score[3] laboratory parameters will be converted in following units:

- Total bilirubin in mg/dL
- Albumin in g/dL
- Creatinine in mg/dL

PELD score = 4.80 * ln(total bilirubin) + 18.57 * ln(INR) - 6.87 * ln(albumin) + 4.36 (if patient <1 year: scores for patients listed for liver transplantation before the patient's first birthday continue to include the value assigned for age (<1 year) until the patient reaches the age of 24 months) + 6.67 (if the patient has growth failure [< -2 StdDev])

Laboratory values <1.0 will be set to 1.0 for the calculation of the PELD score.

MELD score = 9.57 * ln(creatinine) + 3.78 * ln(total bilirubin) + 11.2 * ln(INR) + 6.43

Laboratory values <1.0 will be set to 1.0 and serum creatinine values >4.0 mg/dL (equivalent of 353.6 µmol/L) will be set to 4.0 for calculation of the MELD score.

If a patient goes from 11 years of age to 12 between the beginning and end of study, both PELD and MELD scores will be calculated at the first visit after the 12th birthday and move to MELD score.

[3] https://www.unos.org/wp-content/uploads/unos/MELD_PELD_Calculator_Documentation.pdf; accessed on 25-Jul-2018.

3.3.8 Fibroscan®

Where available, Fibroscan® will be performed as per institution standard practice at Visits 1, 5, 8, and 11. The data collected on the Fibroscan form (liver stiffness measured in kPa and controlled attenuation parameter (CAP) measured in dB/m) will be converted to determine stage of fibrosis and grade of steatosis, respectively, using a score card[4,5] as outlined in Table 1 and Table 2.

Table 1: Fibroscan Scoring for Cholestatic Liver Disease (Fibrosis Score)

SCORE	F0 TO F1 NO SCARRING/ MILD FIBROSIS	F2 MODERATE FIBROSIS	F3 SEVERE FIBROSIS	F4 CIRRHOSIS OR ADVANCED FIBROSIS
Fibroscan result	2 to 7 kPa	>7 to 9 kPa	>9 to 17 kPa	>17 kPa

Table 2: Controlled Attenuation Parameter Score and Steatosis Grading

CONTROLLED ATTENUATION PARAMETER SCORE	AMOUNT OF LIVER WITH FATTY CHANGE	STEATOSIS GRADE
<238	–	S0
238 to 260 dB/m	11% to 33%	S1
260 to 290 dB/m	34% to 66%	S2
>290 dB/m	≥67%	S3

3.3.9 Markers of Fibrosis

Aspartate aminotransferase to platelet ratio index (APRI) score and fibrosis-4 (Fib-4) score will be calculated at Visits 1, 3, 5, 8, and 11.

APRI = [(AST in U/L)/(AST ULN in U/L)] × 100/(platelets in 10^9/L)

Fib-4 score = (age*AST in U/L)/(platelets in 10^9/L * √ (ALT in U/L)).

---

[4] Understanding your Fibroscan Results. Memorial Sloan-Kettering Cancer Center Web site. https://www.mskcc.org/cancer-care/patient-education/understanding-your-fibroscan-results Updated February 27, 2018. Accessed March 27, 2020.

[5] Jewish Hospital Fibroscan Interpretation Assessment SOP. Washington University, Louisville Kentucky. https://louisville.edu/medicine/departments/medicine/divisions/gimedicine/physician-resources/calculators-and-tools-files/fibroscan-interpretation-sheet. Undated.

3.3.10 Liver Biopsy

If a liver biopsy is performed according to the local regulations or standard of care at any time during A4250-008, the biopsy results should be recorded in the eCRF. If the patient has liver biopsy results collected in Study A4250-005 or prior to Week 24 in Study A4250-008, the patient will be asked to consent to a follow-up liver biopsy, if allowed per local regulations, at Visit 11 unless 2 prior liver biopsy results have been collected with at least one year between biopsies. Liver biopsies will not be performed in Spain.

3.3.11 Global Impression of Change and Global Impression of Symptom Measures

Patients, caregivers, and clinicians will complete the global impression of change (GIC) and the global impression of symptom (GIS) measures at Visits 1, 2, 3, 5, 8, and 11, and at all visits during the optional extension period.

The GIC items assess change in itch (patient version), scratching (caregiver and clinician versions), and sleep (all versions) since starting the study drug. The GIS items assess itch (patient version), scratching (caregiver and clinician versions), and sleep (all versions) in the past week.

Caregivers and clinicians will complete the GIC and GIS for all patients; only patients ≥8 years of age will complete the patient version

A detailed description of procedures and assessments to be conducted during this study is summarised in the schedule of study assessments (Table 3).

Table 3: Schedule of Study Assessments

Study Activity	Screening Period (Cohort 2 only)		Treatment Period											Follow-Up Period[t]
			Screening/ Inclusion Visit	4 weeks	12 weeks	22 weeks	24 weeks	36 weeks	46 weeks	48 weeks	60 weeks	70 weeks	72 weeks	76 weeks
Study Days (±Window)	-56 – (-35) ±2	-28 – (-7) ±2	1	28 (±5)	84 (±7)	154 (±7)	168 (±7)	252 (±7)	322 (±7)	336 (±7)	420 (±7)	490 (±7)	504 (±7)	532 (±7)
Clinic Visits	Visit S-1	Visit S-2	Visit 1[a]	Visit 2	Visit 3	Visit 4	Visit 5	Visit 6	Visit 7	Visit 8	Visit 9	Visit 10	Visit 11/EOT[b]/ Start of Optional Extension Period[r]	Visit 12[c]
Informed consent	X		X[d]											
Inclusion/exclusion criteria	X		X											
Demography/Medical and Surgical History	X													
Concomitant medication[e]	X	X	X	X	X	X	X	X	X	X	X	X	X	X
Physical examination[f]	X[f]		X[f]		X[f]		X[f]			X			X	
Skin examination	X		X	X	X		X			X			X	
Vital signs[g]	X	X	X	X	X	X	X	X	X	X	X	X	X	X

(Continued on next page)

Study Activity	Screening Period (Cohort 2 only)		Treatment Period											Follow-Up Period[t]
			Screening/ Inclusion Visit	4 weeks	12 weeks	22 weeks	24 weeks	36 weeks	46 weeks	48 weeks	60 weeks	70 weeks	72 weeks	76 weeks
Study Days (±Window)	-56 – (-35) ±2	-28 – (-7) ±2	1	28 (±5)	84 (±7)	154 (±7)	168 (±7)	252 (±7)	322 (±7)	336 (±7)	420 (±7)	490 (±7)	504 (±7)	532 (±7)
Clinic Visits	Visit S-1	Visit S-2	Visit 1[a]	Visit 2	Visit 3	Visit 4	Visit 5	Visit 6	Visit 7	Visit 8	Visit 9	Visit 10	Visit 11/EOT[b]/ Start of Optional Extension Period[r]	Visit 12[c]
eDiary: itching, scratching, and sleep scores[h]	Daily diary entry							For 21 days before each clinic visit for Visit 6 through Visit 12						
Clinical chemistry[i]	X		X	X	X		X	X		X	X		X	X
Hematology[i]			X	X	X		X	X		X	X		X	X
Urinalysis[i]			X				X			X			X	
International normalized ratio		X		X	X	X		X	X		X	X		X
Serum bile acids[j]	X	X	X	X	X	X	X	X	X	X	X	X	X	X
Autotaxin			X	X			X			X			X	
p-C4			X	X			X			X			X	

(Continued on next page)

Study Activity	Screening Period (Cohort 2 only)		Treatment Period											Follow-Up Period[t]
			Screening/ Inclusion Visit	4 weeks	12 weeks	22 weeks	24 weeks	36 weeks	46 weeks	48 weeks	60 weeks	70 weeks	72 weeks	76 weeks
Study Days (±Window)	-56 – (-35) ±2	-28 – (-7) ±2	1	28 (±5)	84 (±7)	154 (±7)	168 (±7)	252 (±7)	322 (±7)	336 (±7)	420 (±7)	490 (±7)	504 (±7)	532 (±7)
Clinic Visits	Visit S-1	Visit S-2	Visit 1[a]	Visit 2	Visit 3	Visit 4	Visit 5	Visit 6	Visit 7	Visit 8	Visit 9	Visit 10	Visit 11/EOT[b]/ Start of Optional Extension Period[r]	Visit 12[c]
Alfa-fetoprotein			X				X			X			X	
Vitamins A[j], E and 25-hydroxy vitamin D			X		X	X		X	X		X	X		
Abdominal ultrasound			X				X			X			X	
Fibroscan (where available)			X				X			X			X	
QoL questionnaire (PedsQL)			X				X			X			X	
Patient/Caregiver/Clinician Patient Global Impression of Change			X[q]	X	X		X			X			X	

(Continued on next page)

Study Activity	Screening Period (Cohort 2 only)		Treatment Period											Follow-Up Period[t]
			Screening/ Inclusion Visit	4 weeks	12 weeks	22 weeks	24 weeks	36 weeks	46 weeks	48 weeks	60 weeks	70 weeks	72 weeks	76 weeks
Study Days (±Window)	-56 – (-35) ±2	-28 – (-7) ±2	1	28 (±5)	84 (±7)	154 (±7)	168 (±7)	252 (±7)	322 (±7)	336 (±7)	420 (±7)	490 (±7)	504 (±7)	532 (±7)
Clinic Visits	Visit S-1	Visit S-2	Visit 1[a]	Visit 2	Visit 3	Visit 4	Visit 5	Visit 6	Visit 7	Visit 8	Visit 9	Visit 10	Visit 11/EOT[b]/ Start of Optional Extension Period[r]	Visit 12[c]
Patient/Caregiver/Clinician Patient Global Impression of Symptoms			X	X	X		X			X			X	
Pregnancy test[k]	X[k]	X	X[k]	X	X	X	X	X	X	X	X	X	X	X
Telephone contact			Patients will be contacted via telephone for adverse event monitoring at Weeks 8, 18, 30, 42, 54, and 66											
Liver biopsy[l]													X	
Study drug dispensed[m]			X	X	X		X	X		X	X		X[s]	

(Continued on next page)

Study Activity	Screening Period (Cohort 2 only)		Treatment Period											Follow-Up Period[t]
			Screening/ Inclusion Visit	4 weeks	12 weeks	22 weeks	24 weeks	36 weeks	46 weeks	48 weeks	60 weeks	70 weeks	72 weeks	76 weeks
Study Days (±Window)	-56 – (-35) ±2	-28 – (-7) ±2	1	28 (±5)	84 (±7)	154 (±7)	168 (±7)	252 (±7)	322 (±7)	336 (±7)	420 (±7)	490 (±7)	504 (±7)	532 (±7)
Clinic Visits	Visit S-1	Visit S-2	Visit 1[a]	Visit 2	Visit 3	Visit 4	Visit 5	Visit 6	Visit 7	Visit 8	Visit 9	Visit 10	Visit 11/EOT[b]/ Start of Optional Extension Period[r]	Visit 12[c]
Adverse events[no]	Continuous collection													
Study drug compliance evaluated				X	X[o]		X	X[p]		X	X		X[p]	

eCRF: electronic case report form; eDiary: electronic diary; EOT: end of treatment; p-C4: plasma 7α Hydroxy 4-cholesten-3-one; PedsQL: pediatric quality of life; QoL: quality of life.

a For patients in Cohort 1, Visit 1 coincides with Visit 9 in Study A4250-005. Assessments that have been performed at Visit 9 during Study A4250-005 will not be repeated.

b Assessments must also be performed at the time a patient is prematurely withdrawn from the study. If a patient discontinues prior to week 72, additional phone contact will be made every 3 months up to a total study participation of 72 weeks to assess if the patient has had biliary diversion or liver transplantation.

c Assessments must be performed 28 days following the final dose of study drug.

d Only for Cohort 1.

e Includes current medications.

f A complete physical exam will be performed at Visits S-1, 1, 3, 5, 8, and 11/EOT.

g Includes blood pressure, pulse, respiratory rate, temperature, height (or length depending on age) and body weight (using a certified weight scale). Body mass index will be calculated.

(Continued on next page)

h Itching, scratching, and sleep will be assessed via an eDiary (issued with any necessary training to patients/caregivers at Visit 1). Patients/caregivers will be instructed to fill in the eDiary every morning and evening for the first 24 weeks and the last 21 days before all the remaining visits to the clinic. There will be no interruption between Visits 11 and 12. Patients participating in the extension period will stop eDiary entries at Visit 11.

i See Table 4 in Protocol A4250-008 amendment 5 for detailed parameters.

j Patients will fast (only water intake is permissible) for at least 4 hours prior to the collection of samples for serum bile acids and vitamin A. Exceptions can be made for infants, less than 12 months of age, if unable to fast for the entire 4 hours.

k For girls who have reached menarche. Serum test will be performed at Visit S-1 for Cohort 2 only and Visit 1 (for Cohort 1 only); urine test will be performed at all other visits. If a urine pregnancy test is positive, a serum pregnancy test should be performed to confirm the pregnancy.

l If a liver biopsy is performed at any time during A4250-008 according to the local regulations or standard of care, the biopsy results should be recorded in the eCRF. If the patient has liver biopsy results collected in study A4250-005 or prior to Week 24 in study A4250-008, the patient will be asked to consent to a follow up liver biopsy, if allowed per local regulations, at Visit 11 unless 2 prior liver biopsy results have been collected with at least 1 year between biopsies. Liver biopsies will not be performed in Spain.

m Study drug will be taken once daily from Day 1 through Day 504 as described in Protocol A4250-008 amendment 5 Section 8.2.

n Adverse event information will be collected from the time of signing of the informed consent form to study discontinuation.

o For hepatic adverse events and/or hepatic decompensation, a PK sample should be collected as close to the onset of the event as possible

p Dosage form acceptability questions will be asked of the caregiver and/or patient at Visits 3, 6, and 11.

q Not applicable for Cohort 2.

r Patients who wish to continue receiving A4250 after 72 weeks, will have the option to remain on treatment in an extension period until the drug is commercially available, provided continued use is supported by the risk-benefit profile and the subject has not been previously withdrawn or discontinued from study.

s Dispensation only for patients moving into the optional extension period.

t Only for patients who stop treatment at 72 weeks.

4. Study Endpoints

4.1 Primary Efficacy Endpoints

The primary efficacy endpoints are as follows:

- **EU and Rest of the World (RoW):** Change from baseline in fasting serum bile acids after 72 weeks of treatment.

  Change from baseline will be calculated based on the average of the values at Weeks 70 and 72. The baseline fasting serum bile acids value is defined as follows unless otherwise specified:

  Cohort 1: The average of the last 2 values before the first dose of study drug in Study A4250-008. In general, these 2 values are the values of the last 2 assessments of Study A4250-005. If pre-dose assessments are collected in Study 008 for a patient, then the values of pre-dose assessments in Study A4250-008 will be considered first and used to calculate the baseline. These 2 values need to be taken from 2 consecutive scheduled visits or unscheduled visits. If only one value is available from 2 consecutive scheduled visits or unscheduled visits, then that value will be used as baseline.

  Cohort 2: The average of the last 2 values before the first dose of study drug in Study A4250-008.

- **United States (US):** Proportion of positive pruritus assessments at the patient level over the 72-week treatment period using the Albireo ObsRO instrument.

  A positive pruritus assessment is defined as a scratching score of ≤1 or at least a one-point decrease from baseline on the Albireo ObsRO instrument. At each assessment, the AM score will be compared to the baseline AM average, and the PM score will be compared to the baseline PM average. Both AM and PM pruritus assessments will be included in the analysis of this endpoint. AM scores from the period of 14 days before or on the first dose day in Study A4250-008 will be averaged as the AM baseline. PM scores from the period of 14 days before the first dose day in Study A4250-008 will be averaged as the PM baseline. If a patient's baseline average score is ≤ 1, then only the criterion of a one-point drop

from baseline on the Albireo ObsRO instrument will be used to determine whether a pruritus assessment is positive or not for the primary endpoint analysis.

4.2 Secondary Efficacy Endpoints

The secondary efficacy endpoints include the following:

- **EU and RoW:** Proportion of positive pruritus assessments at the patient level over the 72-week treatment period using the Albireo ObsRO instrument.
- **US:** Change from baseline in fasting serum bile acids after 72 weeks of treatment.
- **All Regions:** Change from baseline in serum bile acids at Weeks 4, 12, 22, 24, 36, 46, 48, 60, 70, 72, and 76.

  Proportion of individual assessments meeting the definition of a positive pruritus assessment at the patient level using the Albireo ObsRO instrument from Weeks 0-4, Weeks 0-12, Weeks 0-22, Weeks 0-24, Weeks 0-36, Weeks 0-46, Weeks 0-48, Weeks 0-60, and Weeks 0-70, and the proportion of positive pruritus assessments at each 4-week interval between Visit 1/Screening and Visit 5/Week 24, then by each visit between Visit 5/Week 24 and Visit 12/Week 76

  Proportion of individual AM assessments meeting the definition of a positive pruritus assessment at the patient level using the Albireo ObsRO instrument from Weeks 0-4, Weeks 0-12, Weeks 0-22, Weeks 0-24, Weeks 0-36, Weeks 0-46, Weeks 0-48, Weeks 0-60, Weeks 0-70, and Weeks 0-72, and the proportion of positive pruritus assessments at each 4-week interval between Visit 1/Screening and Visit 5/Week 24, then by each visit between Visit 5/Week 24 and Visit 12/Week 76.

  Proportion of individual PM assessments meeting the definition of a positive pruritus assessment at the patient level using the Albireo ObsRO instrument from Weeks 0-4, Weeks 0-12, Weeks 0-22, Weeks 0-24, Weeks 0-36, Weeks 0-46, Weeks 0-48, Weeks 0-60, Weeks 0-70, and Weeks 0-72, and the proportion of positive pruritus assessments at each 4-week interval between Visit 1/Screening

and Visit 5/Week 24, then by each visit between Visit 5/Week 24 and Visit 12/Week 76.

Number of patients undergoing biliary diversion surgery or liver transplantation: These parameters will be evaluated separately and together at Weeks 24, 48, and 72.

Change in growth from baseline to Weeks 24, 48, and 72 after initiation of odevixibat treatment, defined as the linear growth deficit (height/length for age, weight for age, and BMI) compared to a standard growth curve (Z-score, StdDev from the fiftieth percentile, P50).

Change in APRI score and Fib-4 score from baseline to Week 72.

Change in PELD/MELD score from baseline to Week 72

Change in use of antipruritic medication at Weeks 24, 48, and 72

4.3 Exploratory Endpoints

Exploratory efficacy endpoints include the following:

- Change in serum alanine aminotransferase (ALT), gamma-glutamyl transferase (GGT), and total bilirubin concentration from baseline to Week 72
- Proportion of individual assessments meeting the definition of a positive pruritus assessment at the patient level over the 72-week treatment period. A positive pruritus assessment includes an itch score ≤1, or at least a 1-point decrease from baseline based on the Albireo PRO instrument; only patients ≥8 years of age will complete the Albireo PRO instrument.
- Change from baseline in sleep parameters by each 4-week interval between Visit 1/Screening and Visit 5/Week 24, then by each visit between Visit 5/Week 24 and Visit 11/Week 72 over the 72-week treatment period measured with the Albireo PRO and ObsRO instruments
- Change from baseline in international normalised ratio (INR), albumin, liver enzymes, leukocytes, and platelets

- Change from baseline in measures of bile acid synthesis (autotaxin, p-C4)
- Assessment of global symptom relief at Weeks 4, 12, 24, 48, and 72 as measured by patient, caregiver, and clinician GIC items
- Change from baseline by each 4-week interval between Visit 1/Screening and Visit 5/Week 24, then by each visit between Visit 5/Week 24 and Visit 12/Week 76 in patient-reported and observer-reported night-time itching and scratching severity scores
- Change from baseline by each 4-week interval between Visit 1/Screening and Visit 5/Week 24, then by each visit between Visit 5/Week 24 and Visit 12/Week 76 in patient-reported and observer-reported morning time itching and scratching severity scores
- Change from baseline by each 4-week interval between Visit 1/Screening and Visit 5/Week 24, then by each visit between Visit 5/Week 24 and Visit 12/Week 76 in pooled pruritus score including observer-reported scratching for patients <8 years of age and patient-reported itch severity for patients ≥8 years of age
- Change from baseline in additional patient-reported and observer-reported sleep parameters (e.g. tiredness, number of awakenings) by each 4-week interval between Visit 1/Screening and Visit 5/Week 24, then by each visit between Visit 5/Week 24 and Visit 12/Week 76
- Change from baseline in PedsQL questionnaire
- Change from baseline in stage of liver fibrosis as assessed by Fibroscan® (where available)
- Change from baseline in stage of liver fibrosis, as assessed by post-treatment biopsy (when available)

4.4 Safety Evaluation

Safety criteria are as follows:

- The primary safety analysis for this study will include the incidence of total treatment-emergent adverse events (TEAEs) and TEAEs categorised by causality, severity, and seriousness assessments made by the Investigator. This includes liver-related mortality and liver decompensation events (refer to Protocol Section 10.2.2.3) and all-cause mortality.
- Trends in safety will also be evaluated for the following:
  - Physical examinations
  - Concomitant medications
  - Vital signs
  - Laboratory test results (including clinical chemistry, haematology, urinalysis, alfa-fetoprotein [AFP], vitamins A and E, 25-hydroxy vitamin D, and INR)
  - Abdominal ultrasound
  - Discontinuations due to AEs

5. Sample Size and Power

There is no formal hypothesis testing in this open-label study. The sample size for Cohort 1 was determined in Study A4250-005. For Cohort 1, patients will be enrolled after completion of Study A4250-005 or participation of 12 weeks in that study. The expected proportion of patients with at least 1 event (i.e. surgical bile diversion, liver transplantation, death) in untreated patients is expected to be high enough to detect an improvement after odevixibat treatment. For the other endpoints, mainly descriptive analyses will be performed.

The proportion of patients with at least 1 event (i.e. surgical bile diversion, liver transplantation, death) that can be expected in a non-treated population depends on PFIC type and age distribution of the included patients. The expected proportion of patients with events will be calculated for the study population once each patient's age and PFIC type are known, using the probability that a patient will get an event estimated from the reference population in the ongoing observational cohort study, NAtural Course and Prognosis of PFIC and Effect of Biliary Diversion (NAPPED).

As an example, if the expected proportion of patients with at least 1 event is estimated to be 30% in the study population, and then if the proportion of patients with at least 1 event during odevixibat long-term treatment is shown to be only 10% in the study, the power is approximately 89% to 96% (1-sided test, alpha=2.5%) for a confidence interval (CI) with upper boundary <30% based on a sample size of n=48 to 60. The sample size of 60 for Cohort 2 was estimated based on the availability of target patient population to evaluate the therapeutic benefit for those patients.

6. Interim Analysis

This study is an extension study of Study A4250-005 and will be ongoing at the time of the planned New Drug Application (NDA) and Marketing Authorisation Application (MAA) submission. For the NDA/MAA submission purpose, once the database is locked (planned lock date is 31Aug2020) for Study A4250-005, an interim analysis of Study A4250-008 (planned clean data cut of Study A4250-008 is on 15Jul2020) will be performed to accompany the final analyses of Study A4250-005.

The analyses described in this SAP will be conducted, as appropriate, for the interim analysis based on data available at the time of the data cut-off date. The interim analysis will be conducted for both safety and efficacy mainly based on descriptive statistics. The main time point will be at the end of 24-week treatment period while all data will be included for by-visit summary. These analyses will accompany the Study A4250-005 analyses and provide further information regarding the safety and efficacy of odevixibat dosing for more than 24 weeks. The effects of crossover from a dose of 40 µg/kg/day of odevixibat or placebo to 120 µg/kg/day of odevixibat will be evaluated, if appropriate, in the analyses of an integrated summary of safety (ISS) and an integrated summary of effectiveness (ISE).

7. Analysis Sets

The full analysis set (FAS) will be the only analysis set used in the analyses of this study.

The FAS will consist of all patients who received at least 1 dose of study drug in Study A4250-008. The FAS will be the primary analysis set for all analyses unless otherwise specified.

8. Statistical Considerations and Analysis

8.1 Handling of Missing Data and/or Invalid Data and Outliers

8.1.1 Missing Data Analysis Methods for Efficacy Endpoints

No imputation will be made for any missing data. The assessments after intercurrent events (death, or initiation of rescue treatments such as biliary diversion surgery or liver transplantation) or follow-up assessments (>= last dose day + 15 days) will be excluded from analysis. For eDiary data analysis, data after premature treatment discontinuation (last dose of treatment) will be excluded from analysis

9. Statistical Methods

9.1 General Statistical Conventions

Study data will be reviewed periodically (approximately on a quarterly basis) by a DSMB. For DSMB analyses, the Adjudication Process Document outlines the events that will be adjudicated and see Section 9.8.2. Descriptive statistics are mainly used in this open-label extension study unless otherwise specified. All statistical procedures will be completed using SAS®, Version 9.4 or higher.

Continuous variables will be summarised using descriptive statistics, including the number of patients with non-missing value (n), mean, median, StdDev (or standard error [SE]), minimum, and maximum. The letter “n” will be presented without a decimal point. Minimum and maximum values will be presented to the same precision as in the database. Mean and median will be presented to 1 more decimal place than the minimum and maximum. StdDev or SE will be presented to 1 more decimal place than the mean and median.

For categorical variables, summaries will include counts of patients (frequencies) and percentages. Percentages will be rounded to 1 decimal place. Descriptive summaries of change from baseline in categorical variables will be provided using shift tables, as applicable.

For summary purposes, if not otherwise specified, the baseline value of a parameter is defined as the last non-missing assessment of that parameter before the first dose of

odevixibat in Study A4250-008 (Appendix A). Derived variables used for the analyses are provided in Appendix A.

Analysis windows defined in Appendix B will be applied for all laboratory parameters, questionnaires, vital sign measurements, physical examinations, and other efficacy parameters in the study, unless otherwise specified.

All summaries will be presented for Cohort 1, Cohort 2, and combined for Cohort 1 + Cohort 2, unless otherwise specified. Within Cohort 1, the following subgroups will be presented:

- Odevixibat 40 µg/kg/day (005) to 120 µg/kg/day (008)
- Odevixibat 120 µg/kg/day (005) to 120 µg/kg/day (008)
- Odevixibat All (005) to 120 µg/kg/day (008)
- Placebo (005) to 120 µg/kg/day (008)

Summaries will also be presented for Cohort 1 Placebo (005) to 120 µg/kg/day (008) + Cohort 2. For Cohort 1 + Cohort 2, only safety summaries will be presented.

All patient data, including derived data, will be presented in individual patient data listings. All listings will be sorted by cohort/treatment subgroup, patient number, date/time, and visit. The sex and age for each patient will be stated on each listing. Data listings will be based on all enrolled patients.

9.2 Patient Disposition

All patients who provided informed consent will be included in a summary of patient accountability.

The following categories will be summarised by cohort/treatment subgroup and overall as follows:

- Patients screened
- Screening failures
- Patients eligible to receive dose

- Patients dosed
- Patients completing treatment
- Patients completing the study
- Patients discontinuing treatment (including reasons for treatment discontinuation)
- Patients withdrawing from study early (including withdrawal reason)
- Patients who have undergone biliary diversion surgery and liver transplantation
- Patients who have been listed for liver transplantation

Additionally, analysis populations will be summarised overall and by region (Appendix C).

9.3 Protocol Deviations

All protocol deviations will be reviewed at the final protocol deviations review meeting before final database lock, through clinical input provided by the Sponsor, using the following sources of information:

- Protocol deviation logs, provided by [redacted] Clinical, Pharmacovigilance, Medical, and Data Management.

Important protocol deviations that are likely impact the efficacy and safety will be identified by the study team during the manual review at the data review meeting prior to DB lock. The list of important protocol deviation will be finalized after the data review meeting. The number of patients with important protocol deviations (overall and by deviation) will be summarised by cohort/treatment subgroup and overall. A listing will be provided with all protocol deviations identified based on data recorded on the eCRF and/or protocol deviation logs from [redacted] Medical and Data Management before database lock and documented in the CSR.

9.4 Demographic and Baseline Characteristics

9.4.1 Demographic Characteristics

Age, height, weight, and BMI at baseline and other demographic variables, e.g. age category (<6 months, 6 months to 5 years, 6 to 12 years, 13 to 18 years, and >18 years), sex, race, ethnicity, country, region (Appendix C) will be summarised descriptively by cohort/treatment subgroup and overall.

Demographics data for Cohort 1 will be obtained from Study 4250-005, as appropriate.

9.4.2 Baseline and Disease Characteristics

The following disease characteristics will be summarised by cohort/treatment subgroup and overall: years since PFIC diagnosis; type of PFIC; presence of significant pruritus per Investigator report; at least 1 serum bile acid level >100 μmol/L within 6 months before Screening visit; reasons for discontinuation of historical PFIC-related investigational medications; and diagnostic genetic laboratory test as well as Child-Pugh classification and hepatic impairment classification per NCI Organ Dysfunction Working Group.

Child-Pugh classification: mild (Class A), moderate (Class B), or severe hepatic impairment (Class C) is based on the FDA Guidance for Pharmacokinetics in Patients with Impaired Hepatic Function. Determination of the classification is made based on medical review of baseline laboratory data and medical history.

Hepatic impairment will also be classified per the NCI Organ Dysfunction Working Group classification as mild, moderate or severe (Mansfield et al. 2016)[6]. Both the Child-Pugh and NCI classification will be included in the baseline characteristics and both will be applied in the subgroup analysis based on hepatic impairment status.

The following continuous parameters will also be summarized: estimated glomerular filtration rate (eGFR); baseline Z-score (weight, height, and BMI); and baseline values of alanine aminotransferase (ALT); aspartate aminotransferase (AST); gamma-glutamyl transferase (GGT); total bilirubin; alkaline phosphatase; INR; and vitamins A, E, and 25 hydroxy D.

[6] Aaron S. Mansfield, Michelle A. Rudek, Diana Vulih, Gary L. Smith, Pamela Jo Harris, and S. Percy Ivy (2016). "The effect of hepatic impairment on outcomes in phase 1 clinical trials in cancer subjects".

eGFR will be calculated based on the modified/bedside Schwartz equation. For patients <18 year of age, Bedside Schwartz Equation will be used:
GFR (mL/min/1.73 m²) = (36.2 × Height in cm) / Creatinine in µmol/L

For patients ≥ 18 years, the isotope dilution mass spectrometry (IDMS)-traceable Modification of Diet in Renal Disease (MDRD) Study equation will be used:
GFR (mL/min/1.73 m^2) = 175 × (Creatinine in µmol/L/88.4)$^{-1.154}$ × (Age)$^{-0.203}$ × (0.742 if female) × (1.212 if African American)

A listing will present demographic and baseline characteristics by patient. In addition, an individual listing of liver biopsy results, including historical liver biopsy results for patients in Cohort 2, will be produced.

The results of pathologic variants identified for ATP8B1, ABCB11, ABCB4, or other genes (such as NR1H4, TJP2, DCDC2, CLDN1, MYO5B) will be listed for all patients.

9.4.3 Medical and Surgical History

The frequencies and percentages of patients with reported medical and surgical history will be presented by system organ class (SOC) and preferred term (PT). Medical and surgical history will be coded using Medical Dictionary for Regulatory Activities (MedDRA), Version 23.0. The summary table will be sorted alphabetically by SOC and PT. Medical and surgical history will also be listed.

9.4.4 Prior and Concomitant Medications

Prior and concomitant medications used in this study will be coded using the March 2020 version of the World Health Organisation (WHO) Drug Global Dictionary.

Prior medication: A medication taken by a patient within 3 months before the first dose date of study drug with a recorded medication stop date before the first dose date of study drug is a prior medication. This will not include historical PFIC-related investigational medications collected separately on CRFs.

Concomitant medication: A medication taken by a patient the day on or after the first dose date of study drug or a medication with a start date before the first dose date of study drug without a recorded stop date before the first dose date of study drug is a concomitant medication.

As patients in Cohort 1 will be entering the study directly from Study A4250-005, the summary of prior medications will be provided only for Cohort 2. Concomitant medication use during the study will be summarised descriptively using frequency tables by Anatomical Therapeutic Chemical (ATC) Class 4 and PT by cohort/treatment subgroup and overall for the FAS. ATC class and PT will be presented alphabetically. All prior and concomitant medications will be listed. Details for imputing missing or partial start and/or stop dates of non-study medications are located in Appendix D.

9.5 Extent of Exposure

9.5.1 Treatment Duration

Exposure will be summarised with descriptive statistics (n, mean, StdDev, minimum, median, and maximum) and presented by cohort/treatment subgroup and overall. Categorical summaries of treatment duration will also be provided. Study drug exposure will be derived as follows:

Duration of exposure (in weeks) = (date of last study drug intake – date of first study drug intake + 1)/7.

Investigators are allowed to interrupt the study drug to allow for the resolution of AEs, if necessary. Drug interruptions will not be considered when calculating treatment duration.

9.5.2 Treatment Compliance

Treatment compliance will be assessed by maintaining adequate study drug dispensing records. Treatment compliance over the treatment period will be calculated using 2 sources of data.

9.5.2.1 Source Data from CRFs

Treatment compliance = 100 × [(number of capsules dispensed – number of capsules returned)/number of capsules that should have been taken].

The number of capsules that should have been taken is calculated as the number of days that patient was in the treatment period (exposure as above) multiplied by the number of prescribed capsules to be taken (based on patient's body weight, Protocol Table 2) during the treatment period. The total number of capsules actually taken is the total number of

capsules recorded as taken based on the CRF (number of capsules dispensed minus returned) summed over the treatment period. If the number of capsules returned is confirmed as missing and the study drug is confirmed has not been returned, the derivation will not be done.

9.5.2.2 Source Data from eDiary

Treatment compliance =100 × (total number of capsules taken/total number of capsules planned to be taken). The number of capsules planned to be taken will be estimated based on patient's body weight per Protocol Table 2. The compliance rate will be calculated based on eDiary data during the first 24 weeks only.

The calculated compliance rates will be summarised and the one based on eCRFs will be considered the primary compliance rate. The compliance rates reported by visit on the CRFs also will be summarised.

Descriptive summary statistics will be used to summarise study drug compliance by cohort and overall. The number and percentage of patients with a compliance <80%, between 80% and 120%, and >120% will also be presented.

9.6 Efficacy Analyses

This section addresses the analyses to be conducted on the primary, secondary, and exploratory efficacy variables.

The efficacy analysis will be carried out using the patients from the FAS. Figures for selected efficacy endpoints, such as the primary pruritus endpoint, change in serum bile acids, and change in other patient-reported and caregiver-reported outcomes, will be provided by cohort/treatment subgroup and by visit. Forest plots will be provided for the efficacy subgroup analyses.

For efficacy endpoints derived from central laboratory data, if central laboratory data are not available due to COVID-19 or other reasons, local laboratory data will be used.

9.6.1 Analysis Methods

Descriptive statistics mainly will be used in this open-label extension study unless otherwise specified.

9.6.2 Treatment by Centre Interaction Analysis (Multicentre Study)

NA

9.6.3 Analyses of Primary Efficacy Endpoints

All the primary analyses will be based on the FAS, unless otherwise specified.

For the EU and RoW, change from baseline in fasting serum bile acids will be summarised descriptively at the end of the 72-week treatment period based on the average of the values at Weeks 70 and 72.

Baseline 2, defined in the table of derived variables in Appendix A, will be used to calculate change from baseline for the summary.

For Cohort 1 patients who were on odevixibat in Study A4250-005, change from baseline to end of the 72-week treatment in Study A4250-008 will be analysed by using the 1-sample t-test or the Wilcoxon signed rank test, as appropriate. Baseline 1, defined in the table of derived variables in Appendix A, will be used for this statistical analysis. For the initial interim data cut, the analysis of change from baseline to end of the 24-week treatment in Study A4250-008 will be conducted.

For the US, the proportion of positive pruritus assessments at the patient level over the 72-week treatment period using the Albireo ObsRO instrument will be summarised descriptively. A positive pruritus assessment is defined as a scratching score of ≤1 or at least a 1-point drop from baseline on the Albireo ObsRO instrument. At each assessment, the AM score will be compared to the baseline AM average, and the PM score will be compared to the baseline PM average. Both AM and PM pruritus assessments will be included in the analysis of this endpoint. For analysis purposes, diary entries will be assigned to a study day based on the recorded date regardless recorded time. AM scores from the period of 14 days before or on the first dose day in Study A4250-008 will be averaged as AM baseline. PM scores from the period of 14 days before the first dose day in Study A4250-008 will be averaged as PM baseline. If a patient's baseline score is ≤ 1, then only the criterion of a one-point drop from baseline on the Albireo ObsRO instrument will be used to determine whether a pruritus assessment is positive or not for the primary endpoint analysis. Rounded baseline score will be used for the analysis.

A 95% CI will be provided for the proportion of positive pruritus assessments.

For Cohort 1 patients who were on odevixibat in Study A4250-005, change from baseline to end of the 72-week treatment in Study A4250-008 will be analysed by using the 1-sample t-test or the Wilcoxon signed rank test, as appropriate. Baseline 1, as defined in Study A4250-005, will be used for this statistical analysis. For the initial interim data cut, the analysis of change from baseline to end of the 24-week treatment in Study A4250-008 will be conducted.

9.6.4 Analyses of Secondary Endpoints

All secondary analyses will primarily be summarised descriptively based on the FAS, unless otherwise specified.

The change in secondary endpoints such as serum bile acids, growth, APRI score, Fib-4 score, PELD/MELD will be summarised by visit using descriptive statistics. Change in growth (height for age, weight for age, and BMI for age) will also be displayed using graphical presentations. Baseline 2 will be used to calculate change from baseline for data analysis. For patients in Cohort 1 who were on odevixibat in Study A4250-005, change in growth to Week 72 will be analysed by using a 1-sample t-test or Wilcoxon signed rank test, as appropriate. Baseline 1, defined in the table of derived variables in Appendix A, will be used for this statistical analysis. For the interim data cut, the analysis of change from baseline in growth to end of 24-week treatment in Study A4250-008 will be conducted. The analysis of growth data will be based on calculated values using the software or methods from the Centers for Disease Control and Prevention (CDC) website for patients with age ≥ 2 years old and from the WHO website for patients with age < 2 years old[7,8]. CRF collected growth data will be listed.

For serum bile acids assessments, if a patient has assessments at Weeks 70 and 72, then the average of the 2 assessment values at Weeks 70 and 72 will be used for data analysis. This handling will also be applied to Weeks 22 and 24, and Weeks 46 and 48.

The proportion of individual assessments meeting the definition of a positive pruritus assessment at the patient level on the Albireo ObsRO instrument from Weeks 0-4,

[7] https://www.cdc.gov/nccdphp/dnpao/growthcharts/resources/sas.htm following CDC's method
[8] https://www.cdc.gov/nccdphp/dnpao/growthcharts/resources/sas-who.htm following WHO's method

Weeks 0-12, Weeks 0-22, Weeks 0-24, Weeks 0-36, Weeks 0-46, Weeks 0-48, Weeks 0-60, Weeks 0-70, or the proportion of positive pruritus assessments at each 4-week interval between Visit 1/Screening and Visit 5/Week 24, then by each visit between Visit 5/Week 24 to Visit 12/Week 76 will be summarised descriptively as for the primary analysis for the US. Similar summaries will be provided based on AM scores as well as PM scores.

The number and percent of patients achieving positive pruritus assessment for more than 50% of the time will be summarised by treatment group. Both AM and PM pruritus assessments will be included in the analysis of this endpoint. A cumulative distribution function (CDF) plot showing proportions of patients achieving positive pruritus assessment for more than X% of the time, for X from 0 to 100%, by treatment group will be provided.

Number and percent of patients undergoing biliary diversion surgery and/or liver transplantation will be summarised by using descriptive statistics. A 95% CI also will be presented for the corresponding percentage. These parameters will be evaluated separately and together at Weeks 24, 48, and 72.

Kaplan-Meier curves will be used when appropriate for time-to-event data (that is, time-to-surgical bile diversion or liver transplantation or death). Median event-free times and associated 95% CIs will be calculated using Brookmeyer and Crowley methodology and a log-log transformation for constructing CIs.

Change in use of antipruritic medication at Weeks 24, 48, and 72 from baseline will be provided by a patient listing.

9.6.5 Analyses of Exploratory Endpoints

The exploratory efficacy variables are listed in Section 4.3. All exploratory analyses mainly will be summarised descriptively based on the FAS, unless otherwise specified.

Exploratory variables including ALT; GGT; total bilirubin; INR; albumin; liver enzymes; leukocytes and platelets; measures of bile acid synthesis; Albireo PRO and ObsRO itching/scratching severity scores; Albireo PRO and ObsRO sleep parameters; Global Symptom Relief; and PedsQL will be summarised descriptively. For continuous data, the change from baseline will be analysed in addition to the presentation of actual visit

values. For categorical data, shift tables or frequency and percentages of patients will be presented, as appropriate. A line graph of itching/scratching daily severity scores of Albireo PRO and ObsRO over time will be provided for each patient.

For continuous data, such as ALT; GGT; total bilirubin; INR; albumin; liver enzymes; leukocytes and platelets; measures of bile acid synthesis; and the total score and domain score of PedsQL, the change from baseline will be analysed descriptively in addition to the actual visit values. Global Symptom Relief at Weeks 4, 12, 24, 48, and 72 will be summarised as categorical variables. Time points (visit windows) are specified in Appendix B.

The proportion of individual assessments meeting the definition of a positive pruritus assessment at the patient level on the Albireo PRO instrument completed by patients ≥8 years of age only will also be explored and summarised in a similar fashion as for the secondary endpoint, the proportion of individual assessments meeting the definition of a positive pruritus assessment at the patient level on the Albireo ObsRO instrument.

Change from baseline in PRO/ObsRO itching and scratching severity scores will be summarised by each 4-week interval between Visit 1/Screening and Visit 5/Week 24, then by each visit between Visit 5/Week 24 and Visit 12/Week 76. A summary table will be provided for night-time scores and morning time scores separately.

The pooled pruritus score including observer-reported scratching for patients <8 years of age and patient-reported itch severity for patients ≥8 years of age will be summarised in a similar manner.

The change from baseline in PRO/ObsRO sleep parameters will be summarised at Week 72 as well as by each 4-week interval between Visit 1/Screening and Visit 5/Week 24, then by each visit between Visit 5/Week 24 and Visit 12/Week 76. Sleep parameters include difficulty of falling asleep and staying asleep, tiredness, and the number of awakenings.

Change from baseline in stage of liver fibrosis measured by Fibroscan® will be analysed descriptively. The frequencies of stages may be summarised by shift tables. Steatosis grade analysis will be performed in a similar fashion. Liver fibrosis as assessed by post-

treatment biopsy will be listed and change from baseline may be presented, if appropriate, upon medical review.

9.7 Safety Analyses

All definitions relative to safety endpoints are detailed in Section 4.4.

All the safety analyses will be performed on the FAS for all safety variables specified below and summarised by cohort/treatment subgroup and overall.

For each safety variable, the last value collected before the first dose of study drug will be used as baseline (i.e. Baseline 2 defined in the table of derived variables in Appendix A) for all analyses unless otherwise specified.

9.7.1 Adverse Events

All AEs will be classified by Primary SOC and PT according to MedDRA, Version 23.0.

AEs will be classified as TEAEs and defined as follows:

A TEAE is an AE occurring during the treatment period that a) Has a start date on or after the first dose date of study drug, or b) Has a start date before the date of the first dose date of study drug, but worsened in severity on or after the date of the first dose date of study drug. If an AE started in Study A4250-005 and was ongoing at the time of enrolment in Study A4250-008, the AE will not be considered a TEAE unless it worsens in severity on or after the date of the first dose date of study drug.

AEs with missing start dates, but with stop dates that either overlap with the treatment period or are missing, will be considered TEAEs. A TEAE with missing drug-relationship will be considered as related. A TEAE with missing severity will be considered as severe. Details for imputing missing or partial start dates of AEs are described in Appendix D.

For counting of the number of AEs, if 2 AE records have the same preferred term and the start date of the 2nd AE is the same as or next day after the end date of the 1st AE, then they will be counted as one AE only.

An overall summary of the incidence of TEAEs (number of patients with any events and number of events, if applicable) will include the following:

- All TEAEs
- Drug-related TEAEs (AE will be defined as drug-related if causality is either probably, possibly, or definitely related)
- Severe TEAEs
- TEAEs leading to study discontinuation
- Serious TEAEs
- Drug-related serious TEAEs
- TEAEs leading to death
- Any hepatic TEAEs
  a. Any liver-related TEAE (events that, per the Investigator, are considered related to PFIC)
  b. Any suspected drug-induced liver injury (DILI) TEAEs (as adjudicated by the Data Safety Monitoring Board [DSMB] and defined in Section 9.7.1.2)
  c. Any TEAE of liver decompensation (as defined in Section 9.7.1.2)
  d. Any TEAE in the standardised MedDRA query (SMQ) of *Drug Related Hepatic Disorders; Severe Events Only*
- Any Fat-Soluble Vitamin Deficiency TEAEs refractory to clinically recommended vitamin supplementation (as defined in Section 9.7.1.1)
- Any Clinically Significant Diarrhoea TEAEs (as defined in Section 9.7.1.1)

TEAEs (number of patients with any events and number of events, if applicable) by SOC and PT in each treatment group will be tabulated for the following:

- TEAEs by SOC and PT including fat-soluble vitamin deficiencies, diarrhoea, hepatoxicity (as defined in Section 9.7.1.1). Note that hepatotoxicity will also be included with the tabulation of hepatic events
- All TEAEs by preferred term by descending incidence in Overall column

- TEAEs leading to study discontinuation by SOC and PT
- Serious TEAE by SOC and PT
- Drug-related serious TEAE by SOC and PT
- TEAEs leading to death by SOC and PT
- Common TEAEs (≥10% in overall)
- Hepatic TEAEs
    a. Liver-related TEAEs (events that, per the Investigator, are considered related to PFIC) by SOC and preferred term
    b. Suspected DILI TEAEs (as adjudicated by the DSMB and defined in Section 9.7.1.2)
    c. TEAEs of liver decompensation (as defined in Section 9.7.1.2) by SOC and preferred term
    d. Any TEAE in the standardised MedDRA query (SMQ) of *Drug Related Hepatic Disorders; Severe Events Only*

Summary tables for the number of patients with any TEAEs by SOC and PT by severity (mild, moderate, severe) and by causality (related [possibly, probably and definitely] versus unrelated [unlikely and unrelated]), will also be provided during the treatment period. AEs with the worst severity will be used in the by-severity summaries. Similarly, AEs with the worst causality (most related to treatment) will be used in the by-causality summaries. If severity or causality is missing, data will be imputed to the worst category.

Where a patient has the same AE, based on preferred terminology, reported multiple times in the treatment period, the patient will only be counted once at the preferred terminology level in AE frequency tables. Where a patient has multiple AEs within the same SOC in the treatment period, the patient will only be counted once at the SOC level in AE frequency tables.

In the AE summaries, AEs will be sorted alphabetically by SOC and PT. In addition, the numbers of patients with liver-related mortality, liver-decompensation events, and all-cause mortality will be presented using descriptive statistics. Kaplan-Meier curves may

be used for time-to-event data (time taken to experience liver-decompensation event, all-cause mortality, and biliary diversion surgery in weeks).

All AEs (including pre-treatment and post-treatment AEs), serious adverse events (SAEs), and deaths will be listed. Separate listings for AEs leading to dose interruption, AEs listing to dose change, and AEs of Interest (Fat-Soluble Vitamin Deficiency, Diarrhoea, and Hepatic AEs as defined below) will also be provided.

9.7.1.1 Definition for TEAEs of Fat-Soluble Vitamin Deficiency, Diarrhoea, and Hepatotoxicity

The following TEAEs have been defined based on the population under study:

- New or worsening of fat-soluble vitamin deficiency refractory to clinically recommended vitamin supplementation.

  All Investigator-reported verbatim terms related to decreases in vitamin levels or vitamin deficiency (e.g. preferred term of hypovitaminosis, Vitamin A decreased, Vitamin A deficiency) with the relevant concomitant medication records reviewed by the Medical Monitor, queried as needed, and the appropriate MedDRA preferred terms reported.

- Clinically significant diarrhoea, defined as any of the following:
  - Diarrhoea that persists for 21 or more days without any other aetiology based on medical review of other concurrent AEs for possible other causes of the diarrhoea or diagnostic testing (e.g. viral infections)
  - Reported by the Investigator as severe in intensity or reported as an SAE due to the requirement for hospitalisation or as an important medical event
  - Diarrhoea with concurrent dehydration requiring treatment with oral or intravenous rehydration and/or other treatment intervention based on medical review of AEs and concomitant medications

- Hepatotoxicity: based on the SMQ of *Drug Related Hepatic Disorders; Severe Events Only*

For each of these categories, tabular summaries will be presented by MedDRA SOC and preferred term; the tables will also include the overall incidence of these AEs. Listings of patients with each of the events as defined will be provided.

9.7.1.2 Definitions and Determination of Hepatic Events

A DSMB was formed to independently assure the safety of patients enrolled in Study A4250-008, as well as to evaluate the integrity of study conduct and the data generated. The DSMB reviews the safety data at regular, pre-defined intervals and on an ad hoc basis as needed and makes recommendations regarding patient safety and study continuance (continuation, modification, or termination of the study). The DSMB is comprised of three paediatric hepatologists and an unblinded biostatistician.

As requested by the FDA, the DSMB has been chartered to review data from patients with hepatic AEs, including cases of suspected DILI and patients with liver decompensation events as defined below. The DSMB also reviews events that are reported in the SMQ of *Drug Related Hepatic Disorders; Severe Events Only*. The DSMB reviews each case and provides their expert opinion on the aetiology of the event.

For this review, Albireo prepares slides for presentation at the DSMB meeting on each patient who meets the criteria for hepatic event adjudication. Relevant liver-related laboratory values over time and a narrative summary of relevant information is provided to the DSMB. During the open session of the meeting, an Albireo physician reviews each case with the DSMB members, responds to questions, and/or obtains any additional information requested by the DSMB. Albireo's assessment of the hepatotoxicity aetiology is documented for each event. During the closed session, the DSMB independently assesses the event aetiology which is documented on a Hepatic Event Adjudication Form that is attached to the meeting minutes. If the DSMB requests follow-up information, this is provided at the next scheduled meeting. Treatment assignment remains blinded for this review. The Adjudication Process Document outlines the events that will be adjudicated.

9.7.1.2.1 Suspected Drug-Induced Liver Injury

As outlined in the protocol for Study A4250-008, patients with laboratory criteria that meet any of the following are considered suspected events of DILI and undergo review and adjudication of the event aetiology by the DSMB:

- ALT or AST ≥5 × upper limit of normal (ULN) if ALT or AST is normal at Baseline, or an absolute threshold of 800 U/L, whichever comes first
- ALT or AST ≥3 × Baseline if ALT or AST is abnormal at Baseline, or an absolute threshold of 800 U/L, whichever comes first
- ALT or AST ≥3 × ULN or 800 U/L, whichever comes first, and total bilirubin >2 × ULN
- Doubling of total bilirubin if total bilirubin was <3 mg/dL at Baseline
- Increase in total bilirubin by >3 mg/dL if total bilirubin was ≥3 mg/dL at Baseline
- INR increase >1.5 if INR was normal at Baseline and increase is refractory to Vitamin K administration
- INR increase by >0.4 if INR was abnormal at Baseline and increase is refractory to Vitamin K administration
- Any increase in total bilirubin and transaminases if accompanied by either a symptom of clinical hepatitis (vomiting, nausea, right upper quadrant pain) or immunological reaction (rash or 5% eosinophilia)

9.7.1.2.2 Liver Decompensation Adverse Events

Events either identified by Investigators or meeting the Albireo definition of liver decompensation will undergo review and adjudication by the DSMB. Patients who meet either of the following criteria undergo review and adjudication of the event aetiology by the DSMB for liver decompensation:

- INR elevation >1.5 that is refractory to vitamin K administration
- In a patient with portal hypertension and cirrhosis, transition to decompensated cirrhosis evidenced by any of the following:

- Presence of ascites
- Hepatorenal syndrome
- Portopulmonary hypertension
- Hepatopulmonary syndrome
- Variceal haemorrhage
- Hepatic encephalopathy

9.7.1.2.3 SMQ Drug-Related Hepatic Disorder AEs:

Events in the Standardised MedDRA query SMQ *Drug Related Hepatic Disorders; Severe Events Only* (SMQ No. 20000007) that are not captured in any of the above parameters will be presented to the DSMB for review.

The data of all adjudicated hepatic events provided by the DSMB will also be listed.

9.7.2 Clinical Laboratory Evaluations

Descriptive statistics for clinical laboratory values (in SI units for all tests, and in conventional units for selected tests (Appendix F)) and absolute changes from baseline at each post-baseline visit (Appendix B) will be presented. The change to the last visit will also be summarised. A shift table from baseline to the highest or lowest value for quantitative variables will be presented for the clinical laboratory parameters presented in Table 4.

Central laboratory data will be used for the summary. If central laboratory data are not available due to COVID-19 or other reasons, local laboratory data will be used.

The following figures will be prepared with ALT data:

- Mean (±StdDev) change from baseline over time by cohort and treatment subgroup; 1 line per cohort and treatment subgroup
- Individual change from baseline over time; Cohort 1; 1 line per patient

- Individual change from baseline over time; Cohort 2; 1 line per patient

Clinical laboratory test results will be listed.

Table 4: Laboratory Parameters

Clinical Chemistry	Hematology	Urinalysis	Other Labs
• Albumin<br>• Alanine aminotransferase (ALT)<br>• Alkaline phosphatase (ALP)<br>• Aspartate aminotransferase (AST)<br>• Bilirubin–total and direct<br>• Calcium<br>• Chloride<br>• Creatinine<br>• Creatine kinase (CK)<br>• Gamma-glutamyl transferase (GGT)<br>• Potassium<br>• Sodium	• Haematocrit<br>• Haemoglobin<br>• Platelet count<br>• Red blood cell count<br>• White blood cell count and differential (neutrophils, eosinophils, basophils, lymphocytes, and monocytes)	• Blood<br>• Glucose<br>• Ketones<br>• Leukocytes<br>• Nitrites<br>• pH<br>• Protein	• Vitamin A and E<br>• 25-Hydroxy vitamin D<br>• International normalised ratio (INR)<br>• Alfa-fetoprotein (AFP)<br>• Prothrombin time

Furthermore, a listing of patients meeting the following criteria for liver monitoring will be provided as follows:

- (ALT or AST ≥3× baseline or >= 800 U/L) and total bilirubin >2 ULN at the same visit
- ALT or AST ≥10 ULN or ≥ 5× baseline or ≥800 U/L in presence of normal lactate dehydrogenase (LDH) and creatine phosphokinase (CPK)
- ALT or AST ≥5 ULN (normal baseline) or ≥3× baseline (abnormal baseline) or >= 800 U/L
- INR >1.5 (normal baseline) or increased by >0.4 relative to baseline (abnormal baseline)

- Total bilirubin ≥2× baseline (baseline total bilirubin <3 mg/dL) or increased by ≥3 mg/dL relative to baseline (baseline total bilirubin ≥3 mg/dL)

9.7.3 Vital Sign Measurements

Descriptive statistics for vital signs (temperature, systolic blood pressure, diastolic blood pressure, heart rate, respiratory rate, weight, height, and BMI) and changes from baseline in vital signs at each post-baseline visit will be presented in a summary table. The change to the last visit will also be summarised. A shift table for the number of patients with changes from baseline to the highest or lowest value (Appendix E) will be provided by cohort/treatment subgroup during the treatment period.

Vital signs data will be listed.

9.7.4 Physical Examinations

All physical examination data (general appearance, eyes, ears, nose, throat, head/neck/thyroid, lymph nodes, cardiovascular, lungs/chest, abdomen, genitourinary, extremities, skin, musculoskeletal, neurological, and other body systems) will be summarised at each visit with percentages and frequencies along with abnormalities by cohort/treatment subgroup.

Abdominal ultrasound assessments for the liver, including Riedel's lobe, the portal vein, and the spleen also will be summarised descriptively for each visit.

Skin assessments on a 5-point scale (0-4 from no evidence of scratching to cutaneous bleeding, haemorrhage, scabbing) for face, right arm, left arm, right leg, left leg, and torso will be summarised at each visit with percentages and frequencies by cohort/treatment subgroup.

Physical examination and skin assessment data will be listed (including pre-treatment and post-treatment results).

9.8 Other Analyses

9.8.1 Subgroup Analyses

Subgroup analyses will also be performed for each of the 3 age groups (<6 months, 6 months to 5 years, 6 to 12 years, and 13 to 18 years, >18 years), each PFIC type, region (US, EU, and RoW), sex, race, ethnicity, baseline serum bile acid (≥250 and <250 µmol/L), Child-Pugh classification, Bile Salt Export Pump (BSEP) type of PFIC 2 patients, and the use of UDCA, rifampicin (alone or either one). Subgroup analyses may be conducted for hepatic impairment classification per NCI Organ Dysfunction Working Group (ODWG) if appropriate.

Descriptive summary statistics will be provided for the following parameters:

- Proportion of positive pruritus assessments at the patient level over the 72-week treatment period (primary endpoint)
- Serum bile acids (primary endpoint)
- Laboratory parameters serum bile acids, ALT, and growth (secondary/exploratory endpoints)

9.8.2 Data Safety Monitoring Board

The DSMB will receive data in the form of tables, figures, and listings (provided by unblinded DSMB statistician from [redacted] who is independent to the [redacted] study team). The requirement for blinded or unblinded data is defined in the DSMB charter. Data provided to the DSMB will include, but is not limited to, demographics, baseline characteristics, medical and surgical history, prior and concomitant medications, AE and SAE data (by SOC and PT and by maximum intensity), laboratory data, liver monitoring, vital sign measurements, and abdominal ultrasound and patient disposition data.

10. Changes to Planned Analysis from Study Protocol

The changes to the planned analysis in the protocol are listed below.

1. Endpoints
    a. Removed one secondary efficacy endpoint

i. All-cause mortality

b. Added one safety endpoint

ii. All-cause mortality

2. Data Analysis

a. For the number of patients with events (surgical bile diversion or liver transplantation or death) and time to event, the comparison to the NAPPED population will be not included in this SAP and will be performed separately.

b. The following analysis in the protocol “As an exploratory analysis of pruritus and serum bile acids, a comparison of treatment with A4250 120 µg/kg/day during the first 24 weeks of Study 008 versus placebo patients over the 24-week treatment period from Study 005 will be carried out.” will be conducted in A4250 ISE analysis because Study A4250-008 will not have post-baseline data of Study A4250-005.

11. Appendices

Appendix A: Derived Variables

A list of derived variables for demographic and baseline characteristics, various duration derivations, drug compliance, baseline derivations, and other important derivations applicable for this study is presented in Table 5.

Table 5: Derived Variables for Demographic and Baseline Characteristics, Various Duration Derivations, Drug Compliance, Baseline Derivations, and Other Important Derivations

VARIABLES	FORMULA
**Demographic and Baseline Characteristics**	
Age at informed consent (in years)	Cohort 1: For patients not from France and Germany, age will be calculated based on date of birth. For patients from France and Germany, only birth year was collected, and July 1 was imputed in the eDC system. The CRF collected age months and age years are based on the imputed date of birth. For analysis purpose, age will be calculated based on collected age months and age years on the Study 005 CRF or the external file (primary source).<br>Cohort 2: For patients not from France and Germany, age will be calculated based on date of birth. For patients from France and Germany, only birth year was collected, and July 1 was imputed in the eDC system. The CRF collected age months and age years are based on the imputed date of birth. For analysis purpose, age will be calculated based on collected age months and age years on the Study 008 CRF or the external file (primary source).
BMI (kg/m^2)	Weight (kg)/[height (m^2)
PFIC diagnosis (in years)	Will be derived by SAS YRDIF function<br>YRDIF (date of diagnosis of PFIC, date of informed consent, 'ACT/ACT')
**Derivation of Duration**	
Study day at any visit	Date of interest–date of first dose of study drug. One day is added if this difference is ≥0
Extent of exposure (days)	Date of last study drug intake–date of first study drug intake + 1
**Drug Compliance**	
Compliance based on CRF data	100 × [(total number of capsules dispensed – total number of capsules returned) /(total number of capsules planned to be taken)]
Compliance based on eDiary data	100 × (total number of capsules taken/total number of capsules planned to be taken). This will be considered as the primary compliance rate.<br>The number of capsules planned to be taken will be estimated based on patient's body weight per Protocol Table 2. If a patient's weight changes at any time during the study, dose adjustment will be required.

(Continued on next page)

VARIABLES	FORMULA
**Derivations for Efficacy Parameters**	
Baseline (general)	Baseline 1 is defined as the last value prior to the first dose in Study A4250-005 for patients in Cohort 1.<br>Baseline 2 is defined as the last value prior to the first dose in Study A4250-008 for all patients. For patients in Cohort 1, the pre-dose assessments of Study A4250-008 can be from Study A4250-005.<br>Baseline 2 will be used in all analyses unless otherwise specified.
Baseline for serum bile acids change from baseline	Baseline 1 is defined for patients in Cohort 1 and will be calculated as the average of last 2 values prior to the first dose in Study A4250-005. If only one non-missing value is available, it will be used as baseline.<br>Baseline 2 is defined as follows:<br>Cohort 1: the average of the last 2 values before the first dose of study drug in Study A4250 008. In general, these 2 values are the values of the last 2 assessments of Study A4250-005. If pre-dose assessments are collected in Study 008 for a patient, then the values of pre-dose assessments in Study A4250-008 will be considered first and used to calculate the baseline. These 2 values need to be taken from 2 consecutive scheduled visits or unscheduled visits. If only one value is available from 2 consecutive scheduled visits or unscheduled visits, then that value will be used as baseline.<br>Cohort 2: the average of last 2 values prior to the first dose in Study A4250-008. If only one non-missing value is available, it will be used as baseline.<br>Baseline 2 will be used in all analyses unless otherwise specified.
End value for serum bile acids change from baseline	The end value is the average of the values at Weeks 70 and 72 after the start of treatment. (Appendix B). If one value is missing, then the non-missing value will be used as the end value. If both values are missing, then the end value is missing.

(Continued on next page)

VARIABLES	FORMULA
Baseline, daily, weekly, and monthly score by AM and PM respectively (scratching, itch severity)	For both the Albireo ObsRO scratching item and the Albireo PRO itch severity score:<br>All non-missing AM scores from the period of 14 days before or on the first dose day of study medications will be averaged as baseline.<br>All non-missing PM scores from the period of 14 days before the first dose day of study medications will be averaged as baseline.<br>Baseline score will be considered missing if ≥8 out of 14 assessments in the 14 days are missing (i.e. 50% rule is applied based on the number of planned assessments).<br>A weekly AM (PM) score will be calculated by averaging all non-missing AM (PM) scores in a week. A weekly score will be considered missing if ≥4 out of 7 AM (PM) scores in a week are missing.<br>A monthly AM (PM) score will be calculated by averaging all non-missing AM (PM) scores in a month (28 days). A monthly score will be considered missing if ≥15 out of 28 AM (PM) scores a week are missing.<br>An AM (PM) average score at each visit after Week 24 will be calculated by averaging all non-missing AM (PM) scores between 2 visits with analysis windows applied (Appendix B). 50% rule is applied based on the number of planned assessments.

(Continued on next page)

VARIABLES	FORMULA
AM & PM baseline, daily, weekly, and monthly score (scratching, itch severity)	For both the Albireo ObsRO scratching item and the Albireo PRO itch severity score:<br>A daily AM & PM score will be averaged from the 2 ratings for each day. A daily score will be considered missing if both assessments are missing.<br>A weekly score will be calculated by averaging all non-missing AM and PM scores in a week. A weekly score will be considered missing if ≥8 out of 14 assessments in a week are missing.<br>A monthly score will be calculated by averaging all non-missing AM and PM scores in a month (28 days). A monthly score will be considered missing if ≥29 out of 56 assessments in a month are missing.<br>An average score of AM & PM at each visit after Week 24 will be calculated by averaging all non-missing AM and PM scores between 2 visits with analysis windows applied (Appendix B). 50% rule is applied based on the number of planned assessments.<br>All non-missing AM scores from the period of 14 days before or on the first dose day of study medications, and all non-missing PM scores from the period of 14 days before the first dose day of study medications will be averaged as the AM & PM baseline score. Baseline score will be considered missing if ≥15 out of 28 assessments in the 14 days are missing. Rounded baseline score will be used for the analysis.<br><br>One thing of note:<br>Inclusion criteria No. 4 in Study A4250-005:<br>Cohort 1: Patient must have history of significant pruritus and a caregiver-reported observed scratching in the eDiary average of ≥2 (on 0 to 4 scale) in the 2 weeks prior to randomisation in Study A4250-005.<br>Inclusion criteria No. 4 in Study A4250-008:<br>Cohort 2: Patient must have history of significant pruritus and a caregiver-reported observed scratching or patient reported itching (for patients >18 with no caregiver-reported observed scratching) in the eDiary average of ≥2 (on 0 to 4 scale) in the 2 weeks prior to the screening/inclusion visit (Visit 1) in Study A4250-008.<br>The pruritus score for purposes of establishing eligibility was calculated by taking the average of the worse of the two scores for each day. The values for week one were averaged and the values for week 2 were averaged. The two weekly averages were then averaged to provide a single score. If this score was ≥2 the pruritus eligibility requirement was met. The calculation of pruritus eligibility score is different from the calculation of the baseline pruritus score defined in the SAP.

Variables	Formula
Average of change from baseline from each AM and PM scores (scratching, itch severity)	The calculation below is based on the change from baseline in each AM and PM score. The values based on this calculation will be used for data analysis.<br>Change from baseline (daily) will be calculated by averaging all non-missing values of change from baseline (AM and PM) in a day. A daily change from baseline will be considered missing if both AM and PM change from baseline in a day are missing.<br>Change from baseline (weekly) will be calculated by averaging all non-missing values of change from baseline (AM and PM) in a week. A weekly change from baseline will be considered missing if ≥8 out of 14 change from baseline values in a week are missing.<br>Change from baseline (monthly) will be calculated by averaging all non-missing values of change from baseline (AM and PM) in a month (28 days). A monthly change from baseline will be considered missing if ≥29 out of 56 assessments in a month are missing.<br>Change from baseline at each visit after Week 24 will be calculated by averaging all non-missing values of change from baseline (AM and PM) between 2 visits with analysis windows applied (Appendix B). 50% rule is applied based on the number of planned assessments.

(Continued on next page)

VARIABLES	FORMULA
Baseline, daily, weekly, and monthly (sleep parameters, such as difficulty falling asleep and staying asleep, tiredness, the number of awakenings)	For patient- and observer-reported outcome scores of sleep parameters, the same approach above will be used as for scratching/itch severity by AM and PM, respectively, since there is just one rating per day.
Proportion of positive pruritus assessments	No imputation will be made for any missing data. The assessments after intercurrent events (premature treatment discontinuation, death, or initiation of rescue treatments such as biliary diversion surgery or liver transplantation) will be treated as missing and excluded from analysis. The proportion of positive pruritus assessments will be calculated based on reported eDiary data only. 50% rule will be applied based on the number of planned assessments (Appendix B).
**Derivations for Safety Parameters**	
AE duration (days)	AE end date–AE start date + 1
TEAEs	An AE (classified by preferred term) occurring during the treatment period that a) has a start date on or after the first dose date of study drug, or b) has a start date before the date of the first dose date of study drug, but worsened in severity on or after the date of the first dose date of study drug. If an AE started in Study A4250-005 and was ongoing at the time of enrolment in Study A4250-008, the AE will not be considered a TEAE unless it worsens in severity on or after the date of the first dose date of study drug.

AE: adverse event; BMI: body mass index; CRF: case report form; eDiary: electronic diary; ObsRO: observer-reported outcome; PFIC: progressive familial intrahepatic cholestasis; PRO: patient-reported outcome; SAP: statistical analysis plan; TEAEs: treatment-emergent adverse events

Appendix B: Visit Window

The observation closest to the target day is the measurement used in the analysis for each visit. The following visit window will apply for all laboratory parameters, questionnaires, vital signs, physical measurements and other efficacy parameters in the study, if not otherwise specified.

Table 6: Analysis Visit Window (General)

Timing of Assessment (Days Relative to Treatment)	Visit Name to Display for Analysis	Target Day	Study Day (Relative Day)
Screening (Days -56 to -1)	Baseline		≤ -1
Day 1 Week 1	Baseline		1 (Pre-dose)
Week 4 (± 5 days)	Week 4	28	Post-baseline - 56
Week 12 (± 7 days)	Week 12	84	57–119
Week 22 (± 7 days)	Week 22	154	120–161
Week 24 (± 7 days)	Week 24	168	162–210
Week 36 (± 7 days)	Week 36	252	211–287
Week 46 (± 7 days)	Week 46	322	288–329
Week 48 (± 7 days)	Week 48	336	330–378
Week 60 (± 7 days)	Week 60	420	379–455
Week 70 (± 7 days)	Week 70	490	456–497
Week 72/EOT (± 7 days)	Week 72	504	498–(last dose day + 14)
4 weeks post last dose of study drug (±7 days)	Follow-up	28 post last dose	≥ Last dose day + 15
**For patients who enter optional extension period:**			
Week 72/EOT (± 7 days)	Week 72	504	498– Min(518, start day of optional extension period - 1)
Week 88	Week 88	616	Min(519, start day of optional extension period)–672
Week 104, 120, … (continue every 16 weeks)	Week 104, 120, … (continue every 16 weeks)	7*week #	(7*(week # - 8) + 1) – 7*(week # + 8)

Table 7: Analysis Visit Windows for GIC/GIS, Physical Measurements and Selected Lab Test)

TIMING OF ASSESSMENT (DAYS RELATIVE TO TREATMENT)	VISIT NAME TO DISPLAY FOR ANALYSIS	TARGET DAY	STUDY DAY (RELATIVE DAY)
Screening (Days -56 to -1)	Baseline		≤ -1
Day 1 Week 1	Baseline		1 (Pre-dose)
Week 4 (± 5 days)	Week 4	28	Post-baseline - 56
Week 12 (± 7 days)	Week 12	84	57–126
Week 24 (± 7 days)	Week 24	168	127–252
Week 48 (± 7 days)	Week 48	336	253–420
Week 72/EOT (± 7 days)	Week 72	504	421–(last dose day + 14)
4 weeks post last dose of study drug (±7 days)	Follow-up	28 post last dose	≥ Last dose day + 15
**For patients who enter optional extension period:**			
Week 72/EOT (± 7 days)	Week 72	504	421– Min(518, start day of optional extension period - 1)
Week 88	Week 88	616	Min(519, start day of optional extension period)–672
Week 104, 120, … (continue every 16 weeks)	Week 104, 120, … (continue every 16 weeks)	7*week #	(7*(week # - 8) + 1) – 7*(week # + 8)

Note: Physical measurements include physical examination, voluntary photography and skin examination. Selected lab tests include urinalysis and autotaxin, p-C4.

Table 8: Analysis Visit Window for PedsQL, Fibroscan, Abdominal Ultrasound and AFP

Timing of Assessment (Days Relative to Treatment)	Visit Name to Display for Analysis	Target Day	Study Day (Relative Day)
Screening (Days -56 to -1)	Baseline		≤ -1
Day 1 Week 1	Baseline		1 (Pre-dose)
Week 24 (± 7 days)	Week 24	168	71 –252
Week 48 (± 7 days)	Week 48	336	253–420
Week 72/EOT (± 7 days)	Week 72	504	421–(last dose day + 14)
4 weeks post last dose of study drug (±7 days)	Follow-up	28 post last dose	≥ Last dose day + 15
**For patients who enter optional extension period:**			
Week 72/EOT (± 7 days)	Week 72	504	421– Min(518, start day of optional extension period - 1)
Week 88	Week 88	616	Min(519, start day of optional extension period)–672
Week 104, 120, … (continue every 16 weeks)	Week 104, 120, … (continue every 16 weeks)	7*week #	(7*(week # - 8) + 1) – 7*(week # + 8)

For laboratory and non-laboratory parameters, if a patient has more than one measurement included within a window, the assessment closest to the target day will be used. In case of ties between observations located on different sides of the target day, the earlier assessment will be used. For laboratory parameters, central laboratory results will be used over local laboratory results if both are in a same analysis window.

Derivations for pruritus and other itching, scratching, sleep parameters measured by Albireo ObsRO and PRO instruments will be derived based on the following analysis window. For analysis purposes, diary entries will be assigned to a study day based on the recorded date regardless of recorded time. Please refer to the derivations in the table of derived variables in Appendix A for details.

4-Week/by-visit interval	Intervals to include eDiary data (Days)[a]	Intervals with >=50% of expected data to include patients[b] (# of expected days to report eDiary)
Weeks 1 – 4	1 - 28	Weeks 1 – 4 (28 days)
Weeks 5 – 8	29 – 56	Weeks 5 – 8 (28 days)
Weeks 9 – 12	57 – 84	Weeks 9 – 12 (28 days)
Weeks 13 – 16	85 – 112	Weeks 13 – 16 (28 days)
Weeks 17 – 20	113 – 140	Weeks 17 – 20 (28 days)
Weeks 21 – 24	141 – 168	Weeks 21 – 24 (28 days)
Weeks 34 – 36	169 – 287	Weeks 31 – 41 (21 days)
Weeks 44 – 46	288 – 322	Weeks 42 – 46 (21 days)
Weeks 47 – 48	323 – 378	Weeks 47 – 54 (14 days)
Weeks 58 – 60	379 – 455	Weeks 55 – 65 (21 days)
Weeks 68 – 70	456 – 490	Weeks 66 – 70 (21 days)
Weeks 71 – 72	491 – 504	Weeks 71 – 72 (14 days)
Weeks 72 – 76	>=505	>=505 (28 days)

[a] The proportion of positive pruritus assessments is calculated based on reported data only. The assessments after intercurrent events (premature treatment discontinuation, death, or initiation of rescue treatments such as biliary diversion surgery or liver transplantation) will be excluded from the calculation.
[b] To have an appropriate analysis, only include patients who reported at least 50% of expected data during the interval. Of notes, for Weeks 34 – 36, while eDiary data during weeks 25 to 41 will be included for analysis, 50% rule is applied to Weeks 31 – 41 only to make sure than a patient is on treatment long enough for the analysis of Weeks 34 – 36.

Cumulative interval	Intervals to include ediary data (Days)[a] (# of expected days to report eDiary)	Intervals with >=50% of expected data to include patients[b] (# of expected days to report eDiary)
Weeks 0 – 4	1 – 28 (28 days)	Weeks 1 – 4 (28 days)
Weeks 0 – 12	1 – 84 (84 days)	Weeks 9 – 12 (28 days)
Weeks 0 – 22	1 – 154 (154 days)	Weeks 19 – 22 (28 days)
Weeks 0 – 24	1 – 168 (168 days)	Weeks 21 – 24 (28 days)
Weeks 0 – 36	1 – 287 (189 days)	Weeks 31 – 41 (21 days)
Weeks 0 – 46	1 – 322 (210 days)	Weeks 42 – 46 (21 days)
Weeks 0 – 48	1 – 378 (224 days)	Weeks 47 – 54 (14 days)
Weeks 0 – 60	1 – 455 (245 days)	Weeks 55 – 65 (21 days)
Weeks 0 – 70	1 – 490 (266 days)	Weeks 66 – 70 (21 days)
Weeks 0 – 72	1 – 504 (280 days)	Weeks 71 – 72 (14 days)

[a] The proportion of positive pruritus assessments is calculated based on reported data only when >=50% of expected ediary data is reported. The assessments after intercurrent events (premature treatment discontinuation, death, or initiation of rescue treatments such as biliary diversion surgery or liver transplantation) will be excluded from the calculation.
[b] To have an appropriate analysis, only include patients who reported at least 50% of expected data during the interval. Of notes, for Weeks 0 – 36, while eDiary data during weeks 0 to 41 will be included for analysis, 50% rule is applied to Weeks 31 – 41 only to make sure than a patient is on treatment long enough for the analysis of Weeks 0 – 36.

Appendix C: Definition of Region Variable

The list of country, country code, and region variable are provided in Table 9.

Table 9: Definition of Region Variable

COUNTRY	COUNTRY CODE	REGION VARIABLE
Australia	AUS	RoW
Belgium	BEL	EU
Canada	CAN	RoW
France	FRA	EU
Germany	DEU	EU
Israel	ISR	RoW
Italy	ITA	EU
Netherlands	NLD	EU
Poland	POL	EU
Saudi Arabia	SAU	RoW
Spain	ESP	EU
Sweden	SWE	EU
Turkey	TUR	RoW
United Kingdom	GBR	EU
United States	USA	US

EU: European Union; RoW: rest of world; US: United States

Appendix D: Handling of Missing or Incomplete Dates

Global Statement:

If the imputed date is prior to the date of birth, then impute the missing date as date of birth.

Imputation rules for missing or partial AE start date are defined below:

If only Day of AE start date is missing:

If the AE start year and month are the same as that for the first dose date, then:

- If the full (or partial) AE end date is NOT before the first dose date or AE end date is missing, then impute the AE start day as the day of first dose date
- If AE end date is prior to first dose date, then impute the AE start day as 1

If Day and Month of AE start date are missing:

If AE start year = first dose year, then:

- If the full (or partial) AE end date is NOT before the first dose date or AE end date is missing, then impute the AE start month and day as the month and day of first dose date
- If AE end date is prior to first dose date, then impute the AE start month as January and the day as 1

If Year of AE start date is missing:

If the year of AE start is missing or AE start date is completely missing, then query the site with no imputation. Also compare the full (or partial) AE end date to the first dose date. If the AE end date is before the first dose date, then the AE should be considered as a pre-treatment AE. Otherwise, the AE will be considered as TEAE.

Imputation rules for missing or partial non-study medication start/stop dates are defined below:

Missing or partial non-study medication start date:

- If only day is missing, use the first day of the month
- If day and month are both missing, use the first day of the year
- If day, month, and year are all missing, use the date of the day before the first dose date

Missing or partial non-study medication stop date:

- If only day is missing, use the last day of the month
- If day and month are both missing, use the last day of the year
- If day, month, and year are all missing, assign "continuing" status to stop date

Appendix E: Normal Reference Ranges of Vital Signs[9]

Table 10: Heart Rate by Age (Beats/Minute) Reference

AGE	AWAKE RATE
Infant (<1 year)	100-190
Toddler (1-2 years)	98-140
Preschool (3-5 years)	80-120
School-age (6-11 years)	75-118
Adolescent (12-15 years)	60-100
> 15 years	60-100

Table 11: Normal Respiratory Rate by Age (Breaths/Minute) Reference

AGE	RESPIRATORY RATE
Infants (<1 year)	30-53
Toddler (1-2 years)	22-37
Preschool (3-5 years)	20-28
School-age (6-11 years)	18-25
Adolescent (12-15 years)	12-20
>15 years	12-20

Table 12: Normal Blood Pressure by Age (mm Hg) Reference

AGE	SYSTOLIC PRESSURE	DIASTOLIC PRESSURE
Infant (<1 year)	72-104	37-56
Toddler (1-2 years)	86-106	42-63
Preschooler (3-5 years)	89-112	46-72
School-age (6-9 years)	97-115	57-76
Preadolescent (10-11 years)	102-120	61-80
Adolescent (12-15 years)	110-131	64-83
>15 years	90-120	50-80

[9] Pediatric Vital Signs Reference Chart. Peds cases - Pediatrics for Medical Students - Developed by Chris Novak and Peter Gill for PedsCases.com April 21, 2016 (http://www.pedscases.com/pediatric-vital-signsreference-Chart).

Table 13: Normal Temperature Range by Method

METHOD	TEMPERATURE (°C)
Rectal	36.6-38
Ear	35.8-38
Oral	35.5-37.5
Axillary	36.5-37.5
Temporal/core[a]	35.8-38

[a] In the eCRF core and temporal temperature measurements can be ticked as methods. Rectal and Tympanic (ear) fall in this group. Additionally, temporal temperature measurements (using a temperature scanner on the forehead)–approximate closely to core temperature measurements and therefore can be classified in this category. For that reason, the normal temperature range for core and temporal temperature measurements will be defined as described.

Appendix F: SI and US Conventional Units of Clinical Laboratory Values

SERUM CHEMISTRY	SI UNIT	CONVENTIONAL UNIT
**Analyte**		
Alpha Fetoprotein	IU/mL	ng/mL
Direct bilirubin	µmol/L	mg/dL
Calcium	mmol/L	mg/dL
Chloride	mmol/L	mEq/L
Creatinine	µmol/L	mg/dL
Potassium	mmol/L	mEq/L
Sodium	mmol/L	mEq/L
Serum bile acid	µmol/L	mg/dL
Total bilirubin	µmol/L	mg/dL
**Haematology**	**SI Unit**	**Conventional Unit**
**Analyte**		
Haematocrit	ratio (L/L)	%
Haemoglobin	g/L	g/dL
Red blood count (RBC)	x10ˆ12/L	x10ˆ6/µL
Platelet count	x10ˆ9/L	x10ˆ3/µL
White blood cell count	x10ˆ9/L	x10ˆ3/µL
**Fat Soluble Vitamins**	**SI Unit**	**Conventional Unit**
Vitamin A	µmol/L	ug/dL
Vitamin E	µmol/L	mg/L
Vitamin D (25-dihydroxy)	nmol/L	ng/mL
Vitamin K	nmol/L	ng/mL
**Urinalysis**	**SI Unit**	**Conventional Unit**
**Analyte**		
Glucose	mmol/L	mg/dL
Ketones	mmol/L	mg/dL
Protein	mmol/L	mg/dL
