# Supplementary material for: Interim results from an ongoing, open-label, single-arm trial of odevixibat in progressive familial intrahepatic cholestasis
Source: JHEP Rep. 2023 Apr 29;5(8):100782. doi: 10.1016/j.jhepr.2023.100782 (PMC10338319; doi:10.1016/j.jhepr.2023.100782)
Supplement: Multimedia component 4 [file mmc4.zip › Clinical Trial/CONSORT 2010 Checklist_for Resubmission.pdf]

CONSORT 2010 checklist of information to include when reporting a randomised trial*

Section/Topic	Item No	Checklist item	Reported on page No
**Title and abstract**			
	1a	Identification as a randomised trial in the title	N/A; PEDFIC 2 is not a randomized trial
	1b	Structured summary of trial design, methods, results, and conclusions (for specific guidance see CONSORT for abstracts)	Please see p6 of main text
**Introduction**			
Background and objectives	2a	Scientific background and explanation of rationale	Please see p8 of main text
	2b	Specific objectives or hypotheses	Please see p8 of main text
**Methods**			
Trial design	3a	Description of trial design (such as parallel, factorial) including allocation ratio	Please see p8 to p10 of main text (please note that in the open-label PEDFIC 2 study, all patients receive odevixibat 120 µg/kg/day)
	3b	Important changes to methods after trial commencement (such as eligibility criteria), with reasons	Please see p9 of main text
Participants	4a	Eligibility criteria for participants	Please see p10 of main text
	4b	Settings and locations where the data were collected	Please see p2 of supplement
Interventions	5	The interventions for each group with sufficient details to allow replication, including how and when they were actually administered	Please see p8 to p10 of main text (please note that in the open-label PEDFIC 2 study, all patients receive odevixibat 120 µg/kg/day)
Outcomes	6a	Completely defined pre-specified primary and secondary outcome measures, including how and when they were assessed	Please see p10 to p12 of main text
	6b	Any changes to trial outcomes after the trial commenced, with reasons	N/A
Sample size	7a	How sample size was determined	Please see p4 of supplement
	7b	When applicable, explanation of any interim analyses and stopping guidelines	Please see p10 of main text
Randomisation:			Please note, PEDFIC 2 was NOT a randomized study, so items 8a to 11a are not applicable; rather, PEDFIC 2 is an open-label study and all participants received odevixibat 120 µg/kg/day
Sequence generation	8a	Method used to generate the random allocation sequence	N/A
	8b	Type of randomisation; details of any restriction (such as blocking and block size)	N/A
Allocation concealment mechanism	9	Mechanism used to implement the random allocation sequence (such as sequentially numbered containers), describing any steps taken to conceal the sequence until interventions were assigned	N/A
Implementation	10	Who generated the random allocation sequence, who enrolled participants, and who assigned participants to interventions	N/A
Blinding	11a	If done, who was blinded after assignment to interventions (for example, participants, care providers, those	N/A

		assessing outcomes) and how	
	11b	If relevant, description of the similarity of interventions	N/A
Statistical methods	12a	Statistical methods used to compare groups for primary and secondary outcomes	Please see p12 to p13 of main text
	12b	Methods for additional analyses, such as subgroup analyses and adjusted analyses	Please see p9 of supplement
**Results**			
Participant flow (a diagram is strongly recommended)	13a	For each group, the numbers of participants who were randomly assigned, received intended treatment, and were analysed for the primary outcome	N/A; please note, PEDFIC 2 is an open-label study, and a CONSORT diagram is not appropriate in this instance. However, some elements (eg, number of patients who received treatment, patients who were left the study or were not analyzed, with reasons) are depicted in Figure 1A.
	13b	For each group, losses and exclusions after randomisation, together with reasons	
Recruitment	14a	Dates defining the periods of recruitment and follow-up	Please see p2 of supplement
	14b	Why the trial ended or was stopped	N/A; PEDFIC 2 is ongoing; data included are from the prespecified interim analysis
Baseline data	15	A table showing baseline demographic and clinical characteristics for each group	Please see p30 of main text
Numbers analysed	16	For each group, number of participants (denominator) included in each analysis and whether the analysis was by original assigned groups	Please see p14 to p18 of main text
Outcomes and estimation	17a	For each primary and secondary outcome, results for each group, and the estimated effect size and its precision (such as 95% confidence interval)	Please see p14 to p18 of main text
	17b	For binary outcomes, presentation of both absolute and relative effect sizes is recommended	Please see p13 of supplement
Ancillary analyses	18	Results of any other analyses performed, including subgroup analyses and adjusted analyses, distinguishing pre-specified from exploratory	Please see p5 to p7 and p9 of supplement
Harms	19	All important harms or unintended effects in each group (for specific guidance see CONSORT for harms)	Please see p18 to p20 of main text
**Discussion**			
Limitations	20	Trial limitations, addressing sources of potential bias, imprecision, and, if relevant, multiplicity of analyses	Please see p23 of main text
Generalisability	21	Generalisability (external validity, applicability) of the trial findings	Please see p20 of main text
Interpretation	22	Interpretation consistent with results, balancing benefits and harms, and considering other relevant evidence	Please see p20 to p23 of main text
**Other information**			
Registration	23	Registration number and name of trial registry	Please see p5 and p9 of main text
Protocol	24	Where the full trial protocol can be accessed, if available	N/A
Funding	25	Sources of funding and other support (such as supply of drugs), role of funders	Please see p4 of main text

*We strongly recommend reading this statement in conjunction with the CONSORT 2010 Explanation and Elaboration for important clarifications on all the items. If relevant, we also recommend reading CONSORT extensions for cluster randomised trials, non-inferiority and equivalence trials, non-pharmacological treatments, herbal interventions, and pragmatic trials. Additional extensions are forthcoming: for those and for up to date references relevant to this checklist, see www.consort-statement.org.
